# Trends in knowledge of HIV status and efficiency of HIV testing services in sub-Saharan Africa, 2000–20: a modelling study using survey and HIV testing programme data

**DOI:** 10.1016/S2352-3018(20)30315-5

**Published:** 2021-03-02

**Authors:** Katia Giguère, Jeffrey W Eaton, Kimberly Marsh, Leigh F Johnson, Cheryl C Johnson, Eboi Ehui, Andreas Jahn, Ian Wanyeki, Francisco Mbofana, Fidèle Bakiono, Mary Mahy, Mathieu Maheu-Giroux

**Affiliations:** a Department of Epidemiology, Biostatistics, and Occupational Health, School of Population and Global Health, McGill University, Montréal, Canada; b MRC Centre for Global Infectious Disease Analysis, School of Public Health, Imperial College London, London, UK; c Strategic Information Department, The Joint United Nations Program on HIV/AIDS (UNAIDS), Geneva, Switzerland; d Centre for Infectious Disease Epidemiology and Research, University of Cape Town, Cape Town, South Africa; e Global HIV, Hepatitis and Sexually Transmitted Infections Programme, WHO, Geneva, Switzerland; f Department of Clinical Research, London School of Hygiene & Tropical Medicine, London, UK; g Programme National de Lutte contre le Sida, Abidjan, Côte d'Ivoire; h Department for HIV and AIDS, Ministry of Health and Population, Lilongwe, Malawi; i Ministry of Health, Lilongwe, Malawi and I-TECH, Department of Global Health, University of Washington, Seattle, USA; j Conselho Nacional de Combate ao HIV/SIDA, Maputo, Mozambique; k Conseil National de Lutte contre le Sida et les Infections Sexuellement Transmissibles (CNLS-IST), Ouagadougou, Burkina Faso

## Abstract

**Background:**

Monitoring knowledge of HIV status among people living with HIV is essential for an effective national HIV response. This study estimates progress and gaps in reaching the UNAIDS 2020 target of 90% knowledge of status, and the efficiency of HIV testing services in sub-Saharan Africa, where two thirds of all people living with HIV reside.

**Methods:**

For this modelling study, we used data from 183 population-based surveys (including more than 2·7 million participants) and national HIV testing programme reports (315 country-years) from 40 countries in sub-Saharan Africa as inputs into a mathematical model to examine trends in knowledge of status among people living with HIV, median time from HIV infection to diagnosis, HIV testing positivity, and proportion of new diagnoses among all positive tests, adjusting for retesting. We included data from 2000 to 2019, and projected results to 2020.

**Findings:**

Across sub-Saharan Africa, knowledge of status steadily increased from 5·7% (95% credible interval [CrI] 4·6–7·0) in 2000 to 84% (82–86) in 2020. 12 countries and one region, southern Africa, reached the 90% target. In 2020, knowledge of status was lower among men (79%, 95% CrI 76–81) than women (87%, 85–89) across sub-Saharan Africa. People living with HIV aged 15–24 years were the least likely to know their status (65%, 62–69), but the largest gap in terms of absolute numbers was among men aged 35–49 years, with 701 000 (95% CrI 611 000–788 000) remaining undiagnosed. As knowledge of status increased from 2000 to 2020, the median time to diagnosis decreased from 9·6 years (9·1–10) to 2·6 years (1·8–3·5), HIV testing positivity declined from 9·0% (7·7–10) to 2·8% (2·1–3·9), and the proportion of first-time diagnoses among all positive tests dropped from 89% (77–96) to 42% (30–55).

**Interpretation:**

On the path towards the next UNAIDS target of 95% diagnostic coverage by 2025, and in a context of declining positivity and yield of first-time diagnoses, disparities in knowledge of status must be addressed. Increasing knowledge of status and treatment coverage among older men could be crucial to reducing HIV incidence among women in sub-Saharan Africa, and by extension, reducing mother-to-child transmission.

**Funding:**

Steinberg Fund for Interdisciplinary Global Health Research (McGill University); Canadian Institutes of Health Research; Bill & Melinda Gates Foundation; Fonds the recherche du Québec—Santé; UNAIDS; UK Medical Research Council; MRC Centre for Global Infectious Disease Analysis; UK Foreign, Commonwealth & Development Office.

## Introduction

Efficient and effective HIV testing services are a key component of efforts to end the AIDS epidemic. A positive diagnosis enables people living with HIV to receive life-saving antiretroviral therapy (ART)^1^ and, for pregnant women living with HIV, risk of mother-to-child HIV transmission can be almost entirely prevented.^2^ At the population level, early diagnosis and treatment could reduce incidence by dramatically lowering viraemia such that those with a suppressed viral load are unable to contribute to onward transmission.^3^ HIV testing services also help to identify people who are vulnerable to HIV acquisition and link them to effective HIV prevention services.^4^

In sub-Saharan Africa, where more than two thirds of people living with HIV reside, HIV testing services were initially provided through voluntary counselling and testing upon request in stand-alone sites.^5^ As ART became more widely available, provider-initiated HIV testing and counselling emerged, expanding HIV testing to all patients in health facilities. HIV testing services were also integrated into antenatal care, which greatly increased testing coverage among pregnant and post-partum women.^5^ Such facility-based services were gradually expanded and implementation of community-based services enabled underserved rural and marginalised key populations to be reached by HIV testing and treatment services.^6^, ^7^, ^8^ The development of new testing technologies and strategies—including point-of-care rapid diagnostic tests, self-testing, partner testing, and home-based testing—provided opportunities to accelerate delivery of results and linkage to care.^9^

**Research in context**

**Evidence before this study**

One of the major health policy objectives of the past decade in the global HIV response has been the adoption of targets to end the AIDS epidemic by 2030. UNAIDS and its partners put forth in 2014 the 90-90-90 objectives to increase HIV diagnosis, treatment, and viral load suppression by 2020. There is clear evidence of increases in treatment coverage in sub-Saharan Africa, but little attention has been devoted to the so-called first 90 and trends in knowledge of status have not been systematically reviewed and compared.

We searched PubMed from inception to March 31, 2020, without language restriction with the terms “HIV”[Title/Abstract] AND (“test*”[Title/Abstract] OR “diagnos*”[Title/Abstract] OR “knowledge”[Title/Abstract]) AND (“Africa”[MeSH] OR “Africa”[Title/Abstract]), as well as the UNAIDS and WHO websites for HIV testing reports and guidelines. Several studies and reports present knowledge of status estimates for selected countries, but none comprehensively examined knowledge of status trends by country, age, and sex, or provided estimates of the efficiency of HIV testing services.

**Added value of this study**

Due to incomplete HIV surveillance data, and to non-disclosure of HIV-positive status in most population-based surveys, assessment of knowledge of status is challenging and not uniform in sub-Saharan Africa. By triangulating household survey data about the proportion of adults ever tested for HIV and HIV testing programme data on the total annual number of HIV tests done among adults using a mathematical model of testing behaviours, this study is the first to systematically and comprehensively assess how knowledge of status and efficiency of HIV testing services have evolved in sub-Saharan Africa over 20 years, with stratification by sex, age, and region.

**Implications of the available evidence**

The past two decades have witnessed remarkable increases in knowledge of status across sub-Saharan Africa, but stark sex, age, and regional disparities remain, even in countries that have met the 90% target overall. Concomitant decreases in median time to diagnosis, HIV testing positivity, and proportion of new diagnoses among all positive tests highlight one of the major challenges faced by testing programmes: targeting of HIV testing services to achieve greatest yield of new diagnoses as the undiagnosed population shrinks and diagnosis delays are reduced. With national HIV control programmes now contemplating how to reach the next UNAIDS target of 95% diagnostic coverage by 2025, there is a need to focus on addressing disparities in knowledge of status and to better understand retesting patterns.

Recognising the individual and population benefits of HIV testing and treatment, in 2014, UNAIDS proposed ambitious targets to strengthen the HIV treatment and care cascade such that, by 2020, 90% of people living with HIV would know their status, 90% of those diagnosed would receive ART, and 90% of those treated would have a suppressed viral load, with each target increasing to 95% by 2025.^10^ These targets have been widely adopted globally, and have motivated shifts in the delivery of HIV testing services, especially in sub-Saharan African countries with the greatest epidemic burden. Countries monitor and report annually to UNAIDS their progress towards these targets.

However, the proportion of people living with HIV who know their status is particularly challenging to monitor in sub-Saharan Africa because neither the number of people living with HIV, nor the number who are diagnosed, are directly counted. Estimates for people living with HIV typically come from mathematical models synthesising HIV serosurvey and antenatal testing data—eg, the UNAIDS-supported Spectrum model.^11^ Aggregate HIV testing services data including the number of HIV tests done and number of HIV diagnoses are routinely collected, but reports are often not deduplicated and rates of retesting and re-diagnosis can be high.^12^, ^13^ Household surveys provide cross-sectional data about testing history by HIV status at roughly 5-year intervals in most countries, but only a few surveys directly ask respondents if they are aware of their HIV status, a sensitive question that has high potential for non-disclosure.^14^ These challenges are compounded by imprecise estimates for the number of new infections by age, sex, and geographical area, and by incomplete ascertainment of mortality among the previously diagnosed and undiagnosed population.

Having recently passed the deadline for UNAIDS' interim 2020 target, we sought to evaluate progress towards the so-called “first 90” HIV diagnosis target in sub-Saharan Africa, describe the impact of HIV testing services programmes on knowledge of HIV-positive status and timeliness of HIV diagnosis over the 2000–20 period, and identify remaining gaps in who is being reached by HIV testing services. We synthesised data from 40 sub-Saharan African countries about HIV testing history from population-based surveys, HIV testing programme data, and HIV epidemic indicators using a mathematical model.^12^ In addition to trends in knowledge of status and diagnosis gaps, we estimated time from HIV infection to diagnosis, probability of being tested before reaching a given time since infection or CD4 cell count, percentage of tests found positive, diagnosis yield, and proportion of new diagnoses among positive tests.

## Methods

### Mathematical model

We previously developed and validated a compartmental deterministic mathematical model, Shiny90*,*^12^ to synthesise multiple data sources into a coherent framework to estimate knowledge of status. Briefly, Shiny90 models the transition of individuals aged 15 years or older between six stages: (1) HIV-susceptible individuals who have never been tested, (2) HIV-susceptible individuals who have been tested, (3) people living with HIV who have never been tested, (4) people living with HIV who are unaware of their status and have ever been tested, (5) people living with HIV who are aware of their status and not on ART, and (6) people living with HIV who are on ART. Household surveys and HIV testing programme data are used to estimate the rates of HIV testing among adults not living with HIV and those living with HIV, where HIV testing rates vary with calendar time, sex, age, previous HIV testing status, awareness of status, and, for people living with HIV, CD4 cell count category (as a marker of risk of AIDS-related symptoms motivating care seeking and HIV testing).^12^ In this way, the proportion of people living with HIV who know their status, as estimated by Shiny90, is bounded by ART coverage (minimum) and the proportion of people living with HIV who have ever been tested and received the results (maximum).

### Data sources and model calibration

Shiny90 uses inputs for HIV incidence, mortality, and ART coverage estimated and reported by national governments from the UNAIDS-supported Spectrum modelling software and its Estimation and Projection Package.^11^ Spectrum calculates epidemic statistics stratified by age, sex, CD4 cell count category, and ART status. Parameter estimates for HIV disease progression and mortality, as well as demographic rates, are also informed by Spectrum.

Two main data sources were used for estimation of HIV testing rates during model calibration for each country. The first source was the proportion of individuals aged 15 years or older who self-report having ever been tested for HIV and received the result of their last HIV test, obtained from national household surveys conducted between 2000 and 2019. Estimates were stratified by sex, age (15–24, 25–34, and 35–49 years), and, if available, HIV serostatus. The model was calibrated to data on the proportion ever tested for HIV. We did not calibrate to self-reported awareness of status data, due to evidence of non-disclosure.^14^ The second source was data on the total annual number of HIV tests done among individuals aged 15 years or older and, where available, the total number of positive HIV tests done between 2000 and 2019 reported by national HIV testing programmes.

Countries with at least one available survey that measured HIV seroprevalence, or countries with surveys that did not collect HIV biomarkers but had at least one HIV testing programme dataset including the total number of positive tests between 2000 and 2019, were included in our analyses. These were the minimal set of survey and HIV testing programme data that were required to calibrate the model for a given country. Countries with a population of fewer than 250 000 people, without available survey data, or with only survey data without HIV biomarkers and no HIV testing programme data between 2000 and 2019, were excluded from analyses.

For each country, rates of HIV testing by sex, age, HIV status, and testing and treatment history were estimated by the model using a Bayesian framework. The mode of the posterior distribution was estimated via optimisation with the Broyden-Fletcher-Goldfarb-Shanno algorithm and the posterior density was approximated via a Laplace approximation around the posterior mode.^12^ Conceptually, the HIV testing programme data inform rates of HIV testing in the population, while changes in the proportion ever tested by HIV status, sex, and age, alongside estimates of HIV incidence and mortality, inform the proportion of tests done that are among those being HIV tested or diagnosed for the first time versus repeat testing (ie, adjusting for retesting).^12^

### Statistical analysis

Using Shiny90, we calculated annual (2000–20) proportions of people living with HIV with knowledge of status (percentage of all people living with HIV who have ever tested HIV-positive and are thus aware of their HIV status), positivity (percentage of all HIV tests that are positive), yield of new diagnoses (percentage of new diagnoses out of all HIV tests), and the proportion of new diagnoses out of all positive tests. For post-2019 model predictions, rates of HIV testing were assumed to remain constant at their 2019 values, but with amplified uncertainty guided by variation in historical testing rates. These projections were also guided by historical increases in ART coverage, with coverage achieved in 2020 extrapolated from the rates of ART initiation in 2016–19. No adjustments were made for the possible impact of COVID-19 disruptions in 2020. All indicators can be stratified by sex and age group, and aggregated to the regional level by weighting each country's yearly indicator by the number of people living with HIV. Countries were classified by region using UN definitions.

From the annual testing rates by sex, age, HIV testing history, and CD4 cell count, we calculated several cross-sectional indicators using period life-table methods^15^ that account for the competing risk of AIDS-related death. These include time from HIV infection to diagnosis, probability of getting tested within 1 year after infection, and probability of getting tested before reaching a CD4 count threshold of less than 350 cells per μL. These indicators were calculated annually by constructing period life tables for each of the 16 baseline strata of sex (men *vs* women), age groups (15–24, 25–34, 35–49, and ≥50 years), and HIV testing history (never tested *vs* ever tested). Because the estimates are from period life tables, they reflect the distribution of time to diagnosis if a person who seroconverted in a given year was to experience that year's HIV testing rates by age and CD4 category for their remaining lifetime. Details of the calculations are shown in the appendix (p 1).

We obtained uncertainty intervals by drawing 1000 samples from the posterior distribution of the testing rates estimated by Shiny90. We summarised all indicators using the median and 95% credible intervals (CrIs; 2·5th and 97·5th percentiles) of their posterior distribution. We did all analyses using R (version 3.5.1) and the Rcpp packages, and Shiny90's code is publicly available. We followed the Guidelines for Accurate and Transparent Health Estimates Reporting (appendix p 9).

All analyses were done on anonymised and deidentified data. All survey protocols have been approved by the Internal Review Board of ICF International in Calverton (MD, USA) or by the relevant country authorities. Ethics approval for secondary data analyses was obtained from McGill University's Faculty of Medicine Institutional Review Board (A10-E72-17B).

### Role of the funding source

The funders of the study played no role in study design, data collection, data analysis, data interpretation, or writing of the report.

## Results

**Figure 1:**
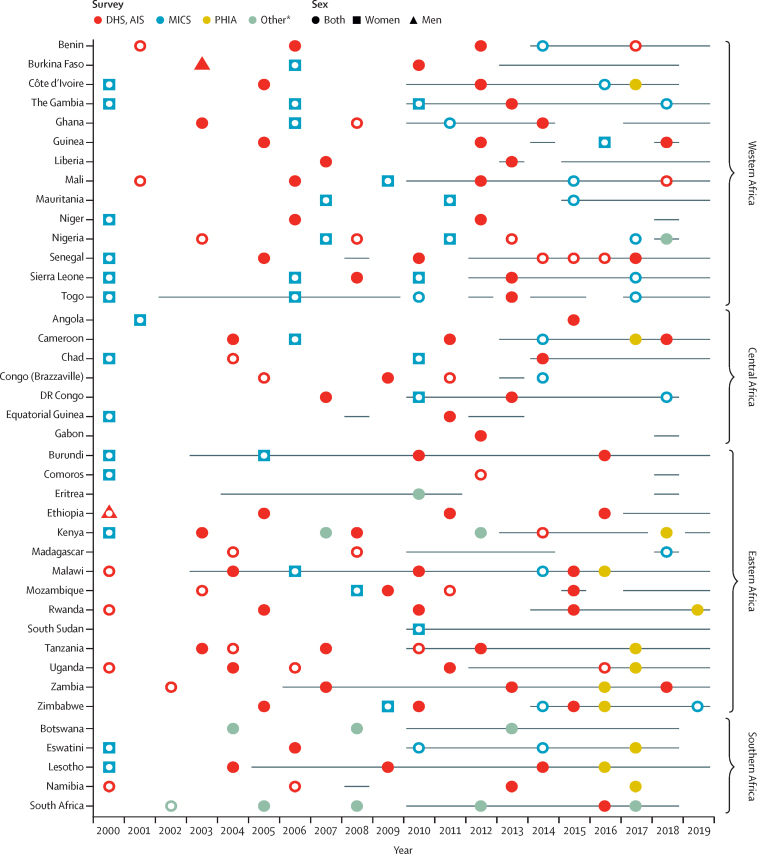
Summary of included surveys and HIV testing services programme data by country and year, 2000–19 Surveys with white dots are those where results on the proportion of individuals who self-report having ever been tested for HIV are not available by HIV status. Horizontal lines represent HIV testing services programme data. DHS=Demographic Health Survey. AIS=AIDS Indicator Survey. MICS=Multiple Indicator Cluster Survey. PHIA=Population-based HIV Impact Assessment Survey. *Other types of surveys include Population Health Survey from Eritrea; South African National HIV Prevalence, Incidence, Behaviour and Communication Surveys; and Botswanan, Kenyan, and Nigerian AIDS Indicator Surveys.

40 countries, 183 population-based surveys (>2·7 millions individuals), and 315 country-years of HIV testing programme data reports informed our model (figure 1). Four sub-Saharan African countries (Cape Verde, Central African Republic, Guinea-Bissau, and Mauritius) were excluded from the analyses due to insufficient data inputs for model calibration, and one (São Tomé and Príncipe), because of high uncertainty in epidemic statistics for small population sizes (<250 000 people). Results of the Shiny90 model calibration are presented in the appendix (pp 16–57).

Across sub-Saharan Africa, the proportion of adults aged 15 years or older estimated to have been tested for HIV, regardless of HIV status, increased by 48 percentage points from 2000 to 2020 (table). Testing coverage was highest in southern Africa, with 85% (95% CrI 83–88) of adults projected to have ever been tested in the region in 2020 (table).

**Table:** Regional progress in HIV testing-related outcomes among adults aged 15 years or older in sub-Saharan Africa, by year

	**Sub-Saharan Africa**	**Western Africa**	**Central Africa**	**Eastern Africa**	**Southern Africa**
**Proportion of individuals ever tested for HIV among overall population aged 15 years or older**					
2000	3·6% (3·0–4·4)	3·1% (2·6–3·8)	1·9% (1·3–2·6)	3·3% (2·8–3·9)	10% (8·3–13)
2005	11% (10–12)	7·2% (6·6–7·8)	7·8% (6·6–9·2)	13% (12–14)	30% (29–32)
2010	30% (29–32)	19% (18–19)	19% (17–22)	41% (39–42)	59% (58–61)
2015	41% (40–42)	28% (27–29)	29% (28–30)	53% (52–54)	75% (74–75)
2020	51% (49–54)	36% (34–39)	42% (39–48)	64% (62–66)	85% (83–88)
**Proportion of people living with HIV who know their HIV status**					
2000	5·7% (4·6–7·0)	4·0% (2·8–5·2)	3·2% (2·0–4·6)	4·8% (4·0–5·8)	9·3% (7·5–12)
2005	20% (18–22)	10% (7·7–12)	15% (12–18)	19% (17–21)	27% (25–30)
2010	53% (50–55)	33% (28–35)	37% (32–41)	56% (53–58)	60% (58–63)
2015	71% (69–73)	52% (48–55)	53% (47–56)	74% (72–75)	79% (77–80)
2020	84% (82–86)	67% (65–69)	70% (64–76)	86% (85–88)	90% (88–92)
**Median time to diagnosis or AIDS-related death, years**					
2000	9·6 (9·1–10)	11 (10–11)	11 (10–12)	11 (10–11)	7·7 (7·0–8·4)
2005	7·2 (6·3–8·0)	10 (9·4–11)	8·2 (7·1–9·3)	7·3 (6·3–8·2)	5·9 (5·1–6·6)
2010	3·6 (3·2–4·1)	5·5 (4·9–6·5)	6·1 (5·2–7·3)	3·2 (2·8–3·6)	2·8 (2·5–3·1)
2015	3·0 (2·7–3·4)	5·2 (4·5–6·0)	4·7 (3·9–6·0)	2·6 (2·4–2·9)	2·2 (1·9–2·4)
2020	2·6 (1·8–3·5)	5·4 (4·1–6·5)	3·9 (2·2–6·0)	2·0 (1·5–2·7)	1·5 (0·9–2·3)
**Probability of getting tested within 1 year after infection**					
2000	2·6% (2·1–3·3)	1·6% (1·1–2·1)	1·5% (1·0–2·1)	1·7% (1·4–2·1)	4·2% (3·3–5·3)
2005	5·9% (4·5–7·7)	1·8% (1·1–2·6)	5·1% (3·6–7·1)	5·8% (4·4–7·6)	7·5% (5·8–9·7)
2010	21% (18–25)	11% (7·7–14)	9·3% (6·5–13)	25% (21–29)	23% (20–26)
2015	26% (23–30)	13% (9·3–17)	12% (7·8–18)	31% (28–35)	28% (25–32)
2020	33% (23–46)	12% (7·0–19)	16% (7·6–34)	40% (29–52)	40% (26–60)
**Probability of getting tested before reaching a CD4 count lower than 350 cells per μL**					
2000	19% (16–23)	13% (9·3–16)	12% (8·3–17)	14% (11–16)	30% (25–35)
2005	35% (29–42)	14% (9·2–19)	30% (22–38)	35% (29–42)	43% (37–50)
2010	63% (58–66)	45% (36–51)	43% (34–51)	66% (62–70)	69% (66–72)
2015	67% (63–70)	47% (38–53)	52% (41–60)	71% (68–74)	75% (72–77)
2020	71% (62–79)	44% (32–56)	59% (39–75)	77% (70–83)	81% (72–89)
**HIV testing positivity**					
2000	9·0% (7·7–10)	3·0% (2·1–3·5)	4·6% (3·5–5·2)	11% (9·6–12)	15% (13–19)
2005	11% (9·2–14)	4·0% (3·3–4·5)	5·4% (4·2–6·7)	10% (9·1–12)	20% (16–26)
2010	5·9% (4·3–8·3)	2·6% (2·0–3·2)	5·0% (3·6–6·9)	5·6% (4·2–7·5)	13% (9·0–22)
2015	4·3% (3·5–5·2)	2·2% (1·7–2·7)	3·4% (2·4–4·7)	4·0% (3·5–4·6)	9·2% (7·1–13)
2020	2·8% (2·1–3·9)	1·9% (1·3–2·7)	2·2% (1·4–3·3)	2·5% (1·9–3·3)	5·5% (3·8–8·4)
**Diagnosis yield**					
2000	7·9% (7·0–8·6)	2·6% (1·8–2·9)	4·2% (3·2–4·7)	9·7% (8·8–11)	13% (12–14)
2005	7·8% (7·0–8·4)	3·2% (2·7–3·6)	3·8% (3·1–4·3)	7·6% (7·0–8·1)	14% (12–15)
2010	2·8% (2·4–3·3)	1·5% (1·2–1·7)	2·6% (2·1–3·0)	2·4% (2·1–2·9)	6·9% (6·2–7·5)
2015	1·9% (1·7–2·1)	1·1% (0·9–1·3)	1·6% (1·2–1·8)	1·7% (1·6–1·8)	4·4% (4·1–4·6)
2020	1·2% (0·9–1·5)	1·0% (0·7–1·5)	0·9% (0·6–1·3)	1·0% (0·8–1·3)	2·2% (1·6–2·9)
**Proportion of new HIV diagnoses among all positive tests**					
2000	89% (77–96)	86% (79–94)	93% (85–97)	91% (85–95)	87% (70–97)
2005	72% (55–86)	79% (71–90)	71% (55–86)	74% (61–84)	70% (48–87)
2010	48% (33–65)	56% (46–73)	52% (36–71)	44% (32–59)	53% (31–76)
2015	44% (37–53)	48% (38–61)	46% (33–59)	42% (37–48)	47% (34–60)
2020	42% (30–55)	52% (38–68)	42% (24–57)	41% (30–52)	39% (25–55)

Numbers in parentheses are 95% credible intervals.

**Figure 2:**
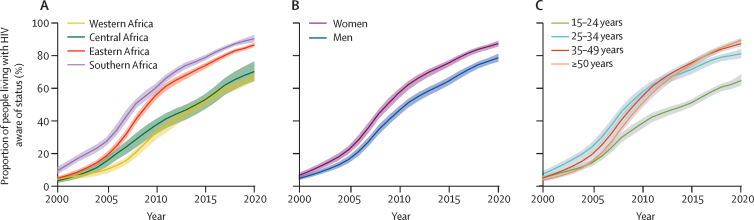
Progress and disparities in knowledge of HIV status in sub-Saharan Africa, 2000–20 Figure shows trends in proportion of people living with HIV who are aware of their HIV status in sub-Saharan Africa by region (A), sex (B), or age group (C). Shaded areas correspond to 95% credible intervals.

**Figure 3:**
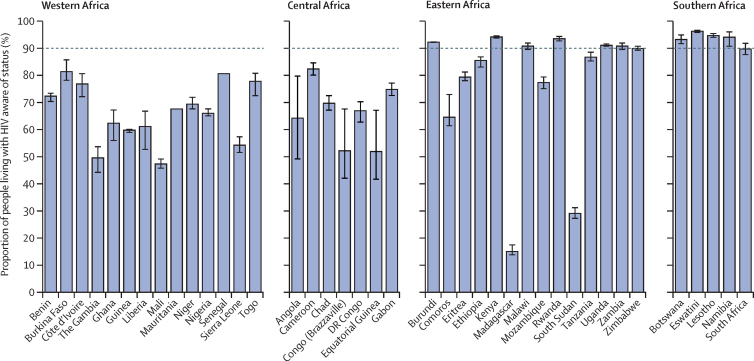
National estimates of knowledge of HIV status in sub-Saharan Africa, 2020 Bars show the proportion of people living with HIV who know their HIV status, with 95% credible intervals represented with vertical lines. The horizontal dashed line represents a threshold of 90% knowledge of status.

The proportion of people living with HIV with knowledge of status increased steadily from 5·7% (95% CrI 4·6–7·0) in 2000 to 84% (82–86) in 2020 in sub-Saharan Africa (table). While knowledge of status increased dramatically in all four sub-Saharan African regions, knowledge of status was consistently lower in western and central Africa than in eastern and southern Africa (figure 2A; appendix p 2). Within the regions, national estimates were also highly heterogeneous, especially in eastern Africa, with a 77 percentage-point difference between the countries with the lowest (Madagascar) and highest (Kenya) knowledge-of-status estimates in 2020. Overall, we projected that 12 countries and one region, southern Africa, reached at least 90% knowledge of status in 2020 (figure 3). Countries with higher knowledge of status tended to be those in which the annual number of tests relative to the total population aged 15 years or older was highest (appendix p 3).

Across sub-Saharan Africa in 2020, men had lower knowledge of status (79%, 95% CrI 76–81) than did women (87%, 85–89), and 15–24-year-olds were the least likely to know their status (65%, 62–69; figure 2; appendix p 11). Such disparities were also observed among the 12 countries projected to achieve at least 90% of knowledge of status overall in 2020. Of these countries, only six (Botswana, Eswatini, Kenya, Lesotho, Namibia, and Rwanda) were projected to achieve 90% of knowledge of status among men, and none were projected to do so among 15–24-year-olds.

**Figure 4:**
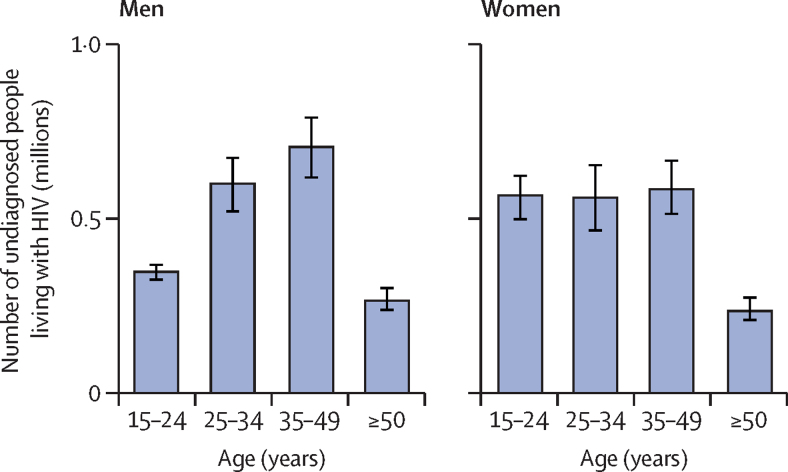
Absolute diagnosis gaps in sub-Saharan Africa, 2020 Vertical lines represent 95% credible intervals.

While the proportion of people living with HIV who are aware of their status was lower among younger adults, the absolute number of people living with HIV was also lower. Consequently, in absolute numbers, the largest group of undiagnosed people living with HIV in sub-Saharan Africa were men aged 35–49 years, with 701 000 (95% CrI 611 000–788 000) left undiagnosed (figure 4; appendix p 10).

**Figure 5:**
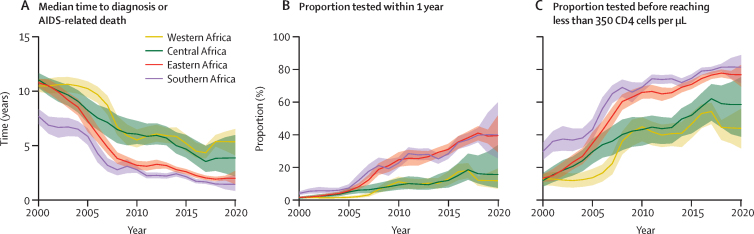
Progress in timeliness of HIV diagnosis in sub-Saharan Africa, 2000–20 Regional trends in median time to diagnosis or AIDS-related death (A) and in the probability of being tested within 1 year of infection (B) or before reaching a CD4 count threshold lower than 350 cells per μL (C) were assessed through period life-table analyses. Shaded areas correspond to 95% credible intervals.

The median time from HIV infection to diagnosis (or death) decreased by 7 years from 2000 to 2020 for all of sub-Saharan Africa (table; figure 5A). That is, if projected HIV testing rates in 2020 persisted into the future, 50% of people infected in 2020 would be diagnosed (or, with small probability, die from AIDS-related causes) within 2·6 years of seroconverting. National trends are presented in the appendix (p 4).

Consistent with the estimated decreases in median time to diagnosis, the probability of receiving an HIV test within 1 year after infection increased by 31 percentage points from 2000 to 2020 in sub-Saharan Africa, and the probability of receiving a test before reaching a CD4 count threshold lower than 350 cells per μL increased by 52 percentage points over the same period (table; figure 5).

The proportion of all HIV tests that are positive (positivity) decreased by 6 percentage points from 2000 to 2020, and the proportion of new diagnoses among all tests (diagnosis yield) decreased by 7 percentage points (table; appendix p 5). Concommitantly, the proportion of new diagnoses among positive tests decreased by 47 percentage points over the study period (table). We project that 58% of people living with HIV undergoing testing in 2020 will have been previously diagnosed with HIV. Quinquennial estimates are presented by sex and age stratification and by region in the appendix (pp 11–15).

## Discussion

Across sub-Saharan Africa, impressive gains were achieved in knowledge of status with 84% (95% CrI 82–86) of people living with HIV being aware of their HIV positive status, and 12 countries and the region of southern Africa projected to have reached the 90% knowledge-of-status target in 2020. Concomitant with these improvements, we estimated that median time from HIV acquisition to diagnosis would have been reduced to 2·6 years (1·8–3·5) by 2020.

Despite this progress, our results highlight substantial regional, national, sex, and age disparities in knowledge of status. Knowledge of status was consistently lower in western and central Africa than in eastern and southern Africa. In those regions, HIV prevalence is lower but key populations—including sex workers, men who have sex with men, and people who inject drugs—account for a higher HIV burden; for example, they generally represent a small fraction of the population but accounted for 42% of all new HIV infections in 2019 in western and central Africa.^16^ Stigma and discrimination towards key populations are common in many health facilities, which might lead to delayed HIV testing or poor uptake of HIV services.^17^ A systematic review and meta-analysis has shown that, among men who have sex with men in Africa, lower testing and knowledge of status were associated with more hostile legislation, and that knowledge of status remained low in the region.^18^ To improve coverage of HIV health services in western and central Africa, antidiscrimination and protective laws to eliminate stigma and discrimination among key populations should be implemented and enforced, health workers trained and sensitised, and key population-friendly services provided.^17^ Eastern Africa, despite having high knowledge of status across the region, includes the two countries with the lowest knowledge of status—South Sudan and Madagascar. While new HIV infections declined overall in eastern African countries between 2010 and 2019, new infections are estimated to have increased by 17% in South Sudan and 191% in Madagascar.^16^ This underscores that reducing new HIV infections, and thus reducing the number of undiagnosed people living with HIV, is key to reaching knowledge-of-status targets.

In all four sub-Saharan African regions, and consistent with previous studies, men are less likely to know their HIV status compared with women.^19^ Large differences in knowledge of status are also observed between age groups, with the lowest proportion diagnosed in those aged 15–24 years. Importantly, all countries have yet to reach 90% knowledge of status in this younger group. This gap between age groups is the natural consequence of HIV transmission dynamics. HIV incidence is highest and average time since infection is short—and thus cumulative exposure to testing is lower—in this age group compared with older ones.^11^ To achieve 90% knowledge of status among 15–24-year-olds would require a simultaneous increase in testing with greater investment in HIV prevention to increase coverage of high-impact prevention interventions.

While we found that knowledge of status was proportionally the lowest among men aged 15–24 years, the largest group of undiagnosed people living with HIV was men aged 35–49 years, with more than 700 000 estimated to be undiagnosed in 2020. Lower uptake of HIV testing among men could be explained by fewer opportunities for testing as well as other social and system-wide barriers such as harmful gender norms^6^, ^20^ and inaccessible or unfriendly services.^21^ Engaging men in HIV prevention efforts is crucially important, not only for their own needs but also for their sexual partners. An increase in knowledge of status and treatment coverage among older men could be crucial in decreasing HIV acquisition rates among women, and by extension, reducing mother-to-child transmission. Among different testing modalities, community-based testing, door-to-door HIV testing services, home-based couples testing, workplace programmes, mobile testing services, social network interventions, incentives to test, self-testing, and partner notification have shown success in increasing diagnostic coverage among men.^22^ As part of these efforts, facilitating linkage to and retaining men in HIV care remains a key challenge for further progress towards HIV testing and treatment targets.

Despite improvements in the median time to diagnosis (or AIDS-related death), if testing levels were maintained across sub-Saharan Africa in 2020, a projected 50% of people living with HIV would not be diagnosed within 3 years following their infection, and 29% would not be tested before reaching a CD4 count threshold lower than 350 cells per μL. These diagnostic delays impede rapid ART initiation, contributing to increased HIV morbidity and onwards HIV transmission.^1^, ^3^ Earlier diagnosis should be accompanied by rapid ART linkage and long-term adherence to ART; these are crucial to minimising morbidity and reducing HIV incidence.^1^, ^3^

As the undiagnosed population shrinks and diagnosis delays are reduced, prioritisation of HIV testing services to achieve greatest yield of new diagnoses is one of the major challenges faced by testing programmes.^23^ Although we noted an ecological correlation between a country's testing volume with respect to its population of reproductive age and knowledge of status, we also estimated a decline in positivity and in yield of new diagnoses. Such declining yields are an inevitable consequence of reaching saturation in testing programmes: as long as testing rates are lower in previously diagnosed individuals than in undiagnosed individuals, we can expect yields to decline as knowledge of status increases. Our analyses also highlight substantial retesting of people living with HIV who are already aware of their status. We projected that 58% of positive tests will have been done on previously diagnosed people living with HIV in sub-Saharan Africa in 2020. In previous studies done in sub-Saharan Africa between 2004 and 2018, retesting among people living with HIV with known HIV status was also common, ranging from 13% to 68%.^13^, ^24^, ^25^, ^26^, ^27^, ^28^ Retesting can be motivated by multiple factors, one of them being the ability to confirm the accuracy of the initial test result.^28^, ^29^, ^30^ Another important driver of retesting might be avoiding disclosing one's HIV-positive status due to societal stigma or denial. A recent study done among people undergoing HIV testing at a health facility in South Africa found that 50% of patients testing HIV-positive had previously been in HIV care (and hence previously diagnosed). Among these, half did not disclose existing knowledge of HIV status to their health-care provider.^13^ Further research is needed to assess the potential benefits of retesting for re-engaging people living with HIV in care.

This analysis has some limitations. First, Shiny90 does not provide estimates of diagnosis coverage among individuals younger than 15 years of age, nor can it disaggregate metrics by key population groups. Second, we could have overestimated knowledge of status in some low HIV-prevalence countries where key populations are disproportionately affected by HIV if these groups are under-represented in population-based surveys. Third, uncertainty in the denominator of people living with HIV, HIV incidence estimates, and ART coverage are not accounted for. This does not affect the validity of point estimates, but their precision could be overestimated. Fourth, we assumed that HIV testing does not result in false-negative or false-positive results. The assumption of no false-negative results might have slightly overestimated knowledge of status and testing probabilities, and underestimated median time to diagnosis. The number of HIV diagnoses reported in HIV testing programme data could be inflated if WHO-recommended retesting to verify HIV diagnosis before ART initiation was incorrectly counted as separate HIV diagnoses, which our model would not be able to identify from routinely reported data. Fifth, we also assumed that self-reporting of HIV testing histories was accurate, but social desirability and recall biases could result in underestimation of the proportion ever tested and, ultimately, of knowledge of status. However, cross-validation of self-reported HIV testing histories with antiretroviral biomarker data from Eswatini, Malawi, Tanzania, and Zambia suggest that self-reported HIV testing history resulted in few false negatives.^31^ Sixth, earlier estimates of diagnosis delays are informed by relatively few population-based survey estimates and HIV testing programme data. Given the cross-sectional nature of these metrics, they could be more sensitive to the elicited model's prior distributions in early years. Finally, the impact of measures taken to prevent the spread of COVID-19 in some countries could have affected both HIV incidence and HIV testing services.^32^ Such unaccounted factors could potentially lead to slightly lower knowledge-of-status estimates than those projected for 2020; however, a notable decrease would be unlikely since people living with HIV who had already been diagnosed would remain so.

Although previous studies have examined HIV testing uptake or self-reported knowledge of status at the community or country level, our analysis systematically and comprehensively assesses how the efficiency of HIV testing services evolved in sub-Saharan Africa over two decades. By using a unified framework to compare HIV testing services metrics, consistency and comparability of results between the different outcomes, countries, and regions is improved. A second strength is the large number of surveys and programme data used for triangulation, improving the precision and robustness of our results. Third, in assessing time to diagnosis and other related metrics, we provide valuable information to help programmes to optimise the efficiency of HIV testing services.^33^ With clear individual and population-health benefits of early treatment initiation, reducing diagnostic delays and improving linkage to care will contribute towards the ultimate goal to end the AIDS epidemics by 2030.

In 2014, the world adopted the goal of achieving 90% HIV diagnosis coverage by 2020. Sub-Saharan Africa, the most affected region globally, is close to reaching this target, with a dozen countries projected to have reached that goal in 2020. However, reaching high diagnosis coverage remains challenging and our results shed light on stark sex and age gaps in knowledge of HIV status. National HIV control programmes are now contemplating how to reach the next UNAIDS target of 95% diagnostic coverage by 2025 in a context of declining positivity, declining yields of true new diagnoses, and COVID-19 disruptions. Reaching this objective will require a better understanding of retesting patterns and a focus on addressing disparities among older men and young people in knowledge of status.

## Data sharing

Data used in this study are those from the Shiny90 country files that were submitted to UNAIDS in 2020. These files, that include Spectrum, surveys, and programme data, can be accessed online. Additional programme data sources are listed in appendix (pp 6–8). The code for the Shiny90 model can be accessed online.
