# Supplementary material for: Trends in knowledge of HIV status and efficiency of HIV testing services in sub-Saharan Africa, 2000–20: a modelling study using survey and HIV testing programme data
Source: Lancet HIV. 2021 Mar 2;8(5):e284–93. doi: 10.1016/S2352-3018(20)30315-5 (PMC8097636; doi:10.1016/S2352-3018(20)30315-5)
Supplement: Supplementary appendix [file mmc1.pdf]

THE LANCET
HIV

Supplementary appendix

This appendix formed part of the original submission and has been peer reviewed. We post it as supplied by the authors.

Supplement to: Giguère K, Eaton JW, Marsh K, et al. Trends in knowledge of HIV status and efficiency of HIV testing services in sub-Saharan Africa, 2000–20: a modelling study using survey and HIV testing programme data. *Lancet HIV* 2021; published online March 2. http://dx.doi.org/10.1016/S2352-3018(20)30315-5.

Supplemental Materials

Table of contents

Text S1. Estimating diagnosis delays. 1

Figure S1. National progress in knowledge of HIV status in sub-Saharan Africa, 2000-2020. 2

Figure S2. Correlation between knowledge of HIV status and ratio of total number of tests relatives to the population aged ≥15 years in sub-Saharan Africa, 2020. 3

Figure S3. National progress in median time to diagnosis or AIDS-related death in sub-Saharan Africa, 2000-2020. 4

Figure S4. Regional trends in positivity and diagnosis yield, 2000-2020. 5

Table S1. Supplemental HIV testing services programme data sources. 6

Table S2. GATHER checklist of information that should be included in new reports of global health estimates. 9

Table S3. Number of undiagnosed people living with HIV by sex and age stratification and by sub-Saharan African region in 2020 (in 1,000s). 10

Table S4. Progress in HIV testing related outcomes in sub-Saharan Africa stratified by sex and age group, 2000-2020. 11

Table S5. Progress in HIV testing related outcomes in Western Africa stratified by sex and age group, 2000-2020. 12

Table S6. Progress in HIV testing related outcomes in Central Africa stratified by sex and age group, 2000-2020. 13

Table S7. Progress in HIV testing related outcomes in Eastern Africa stratified by sex and age group, 2000-2020. 14

Table S8. Progress in HIV testing related outcomes in Southern Africa stratified by sex and age group, 2000-2020. 15

Text S2. Comparisons of calibrated *Shiny90* model fits with programmatic and survey data for each of the 40 included sub-Saharan countries, 2000-2020. 16

***Text S1*. Estimating diagnosis delays.**

From the annual sex- , age- , HIV testing history- , and CD4 cell count-specific testing rates, we calculated several cross-sectional indicators using period life table methods[17] that account for the competing risk of AIDS-related death. For each stratum, we proceed by initializing the life table at infection (time 0: t_0) using the age-specific CD4 cell count distribution at infection (either ≥500 or 350-499 CD4 cells per µL) as is used in Spectrum. We then calculate the number of individuals that will either receive a positive test result or die of AIDS-related cause at each time step ($\Delta t = 0.1$ year), accounting for CD4 cell count progression. As these are period life tables, estimates reflect the distribution of time to diagnosis if a person who seroconverted in year y experienced the HIV testing rates by age and CD4 category estimated for the that year for their remaining lifetime. Specifically, the life tables are constructed using the following equations (for $t \geq t_0$):

$$I_{y(t+\Delta t)}^{kisu} = \begin{cases} I_{yt}^{kisu} - \left(\left(\tau_y^{ki_{[t]}s_{[t]}u} + \epsilon_{is_{[t]}} + \gamma^{is}\right) I_{yt}^{kisu}\right)\Delta t; \quad if\ s = 1 \\ I_{yt}^{kisu} + \left(\gamma^{i(s-1)} I_{yt}^{ki(s-1)u} - \left(\tau_y^{ki_{[t]}s_{[t]}u} + \epsilon_{is_{[t]}} + \gamma^{is}\right) I_{yt}^{kisu}\right)\Delta t; \quad if\ 1 < s < 7 \\ I_{yt}^{kisu} + \left(\gamma^{i(s-1)} I_{yt}^{ki(s-1)u} - \left(\tau_y^{ki_{[t]}s_{[t]}u} + \epsilon_{is_{[t]}}\right) I_{yt}^{kisu}\right)\Delta t; \quad if\ s = 7 \end{cases}$$

where I_{yt}^{kisu} is the number of individuals who seroconverted during calendar year y, from sex k, age i, CD4 cell count s (where 1 indexes CD4 counts of ≥500 cells per µL, 2 for 350-499, 3 for 250-349, 4 for 200-249, 5 for 100-199, 6 for 50-99, and 7 for <50 CD4 cells per µL), and testing history u, at time t since infection; γ^{is} is the CD4 cell count progression rates; $\tau_y^{ki_{[t]}s_{[t]}u}$ is the HIV testing rate specific to sex k, age ($i_{[t]}$, accounting for aging as a function of time t), CD4 cell count ($s_{[t]}$, as CD4 cell count decreases as a function of time t), and testing history (u) that were estimated for the calendar year y in which an individual seroconverted; and $\epsilon_{is_{[t]}}$ is the age- (at infection) and CD4 cell count-specific AIDS-related mortality rate.

After constructing the life table, we calculated the median time between HIV infection and diagnosis or AIDS-related death (m_y^{kiu}) by finding the time t after which more than half of the newly seroconverted individuals had either been diagnosed or died of HIV ($d_y^{kiu}(t)$):

$$d_y^{kiu}(t) = \frac{\sum_{t_0}^{t} \sum_s \left(\left(\left(\tau_y^{ki_{[t]}s_{[t]}u} + \epsilon_{is_{[t]}}\right) I_{yt}^{kisu}\right)\Delta t\right)}{\sum_s I_{yt_0}^{kisu}}$$

$$m_y^{kiu} = \min_{t>0}\left\{\left|d_y^{kiu}(t) - 0.5\right|\right\}$$

Finally, the probability of getting tested within one year of infection (p_y^{kiu}), and of getting tested before reaching a CD4 cell count threshold lower than 350 cells per µL (s=3) (c_y^{kiu}) were estimated by accounting for the competing risk of AIDS-related death using, respectively:

$$p_y^{kiu} = \frac{\sum_{t_0}^{1} \sum_s \left(\tau_y^{ki_{[t]}s_{[t]}u} I_{yt}^{kisu} \Delta t\right)}{\sum_s I_{yt_0}^{kisu}}$$

$$c_y^{kiu} = \frac{\sum_s^3 \sum_t \left(\tau_y^{ki_{[t]}s_{[t]}u} I_{yt}^{kisu} \Delta t\right)}{\sum_s I_{yt_0}^{kisu}}$$

For each calendar year between 2000 and 2020, we estimated these indicators for the 16 age/sex/testing history strata separately. They were then aggregated to the desired level of aggregation (e.g., age, sex, country, region) by weighting each stratum by the estimated number of new HIV infections in that stratum for that year (obtained from Spectrum).

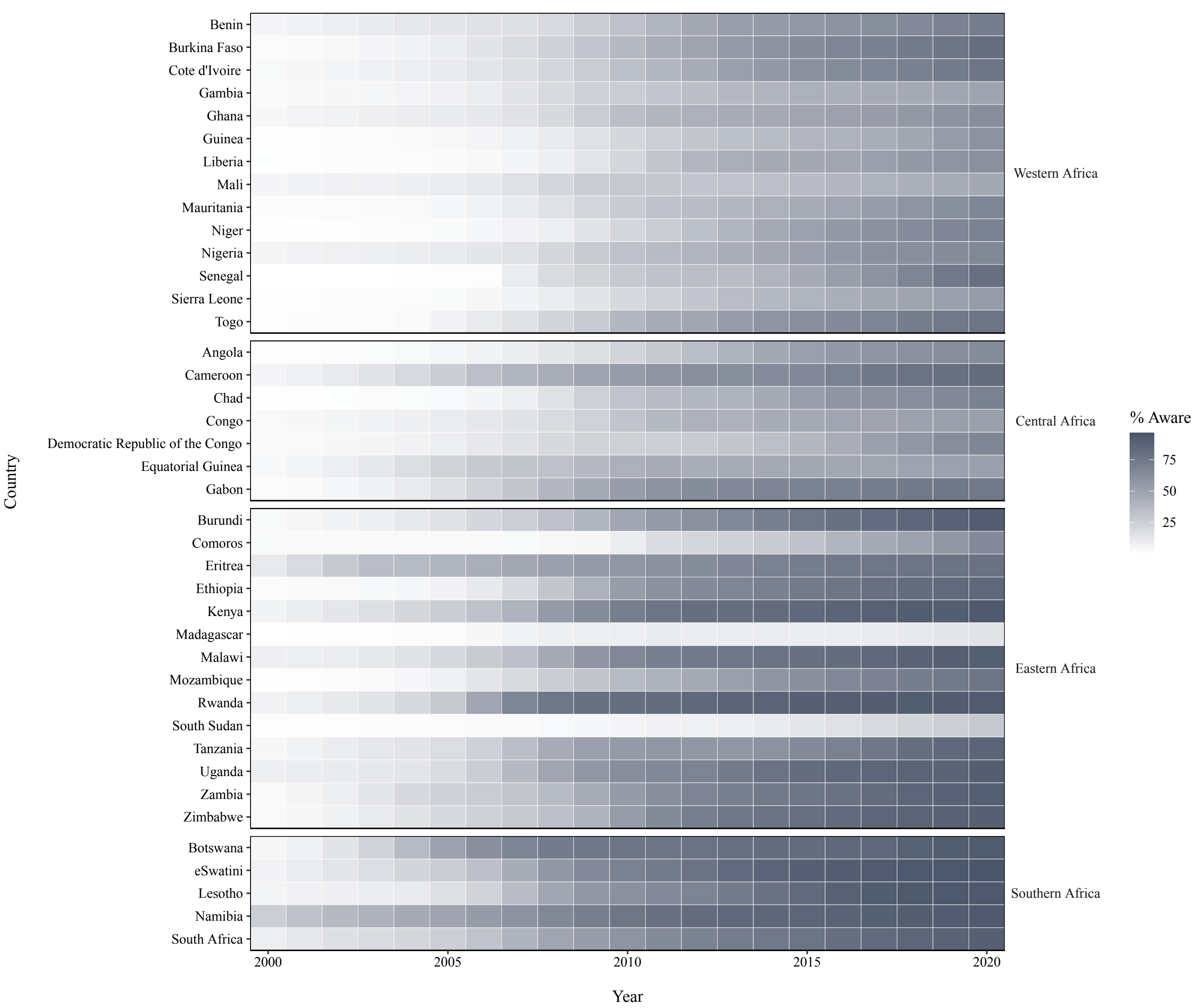

***Figure S1*. National progress in knowledge of HIV status in sub-Saharan Africa, 2000-2020.**

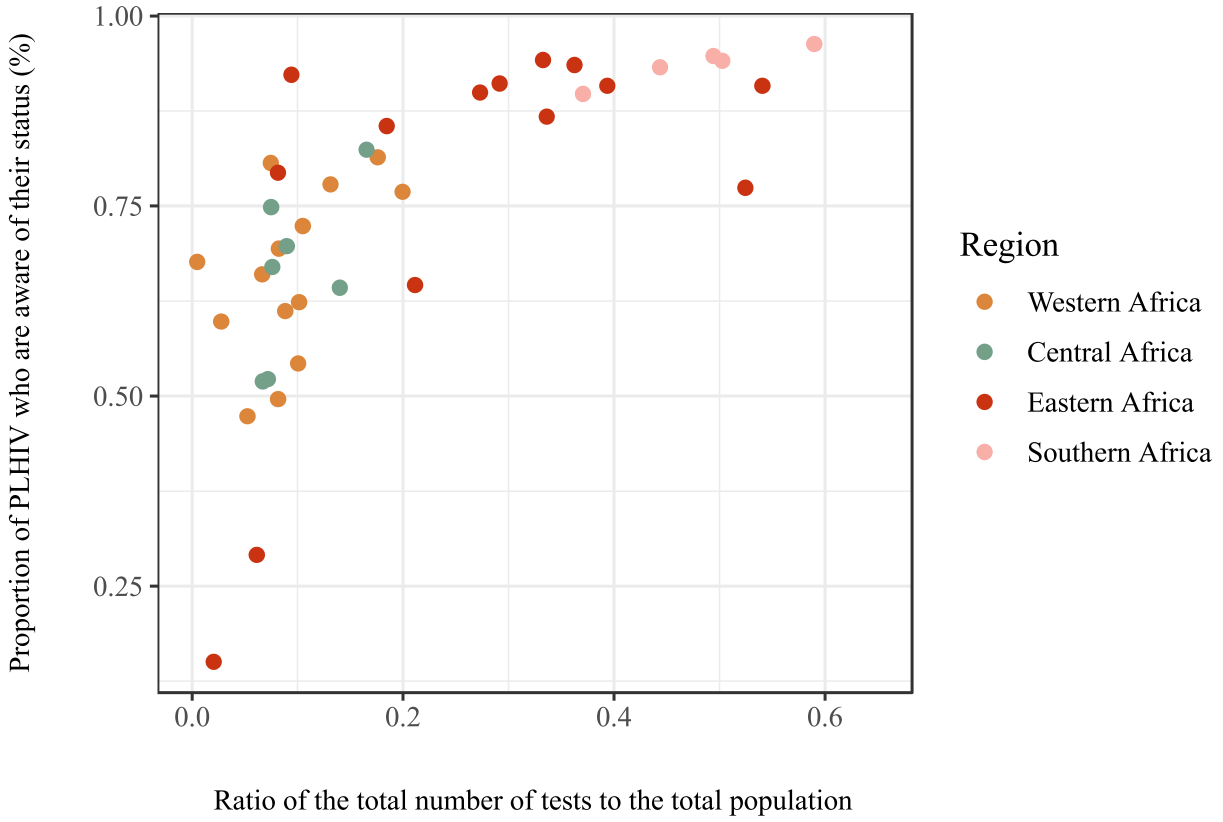

***Figure S2*. Correlation between knowledge of HIV status and ratio of total number of tests relatives to the population aged ≥15 years in sub-Saharan Africa, 2020.**
Each point represents a different country (n = 40).

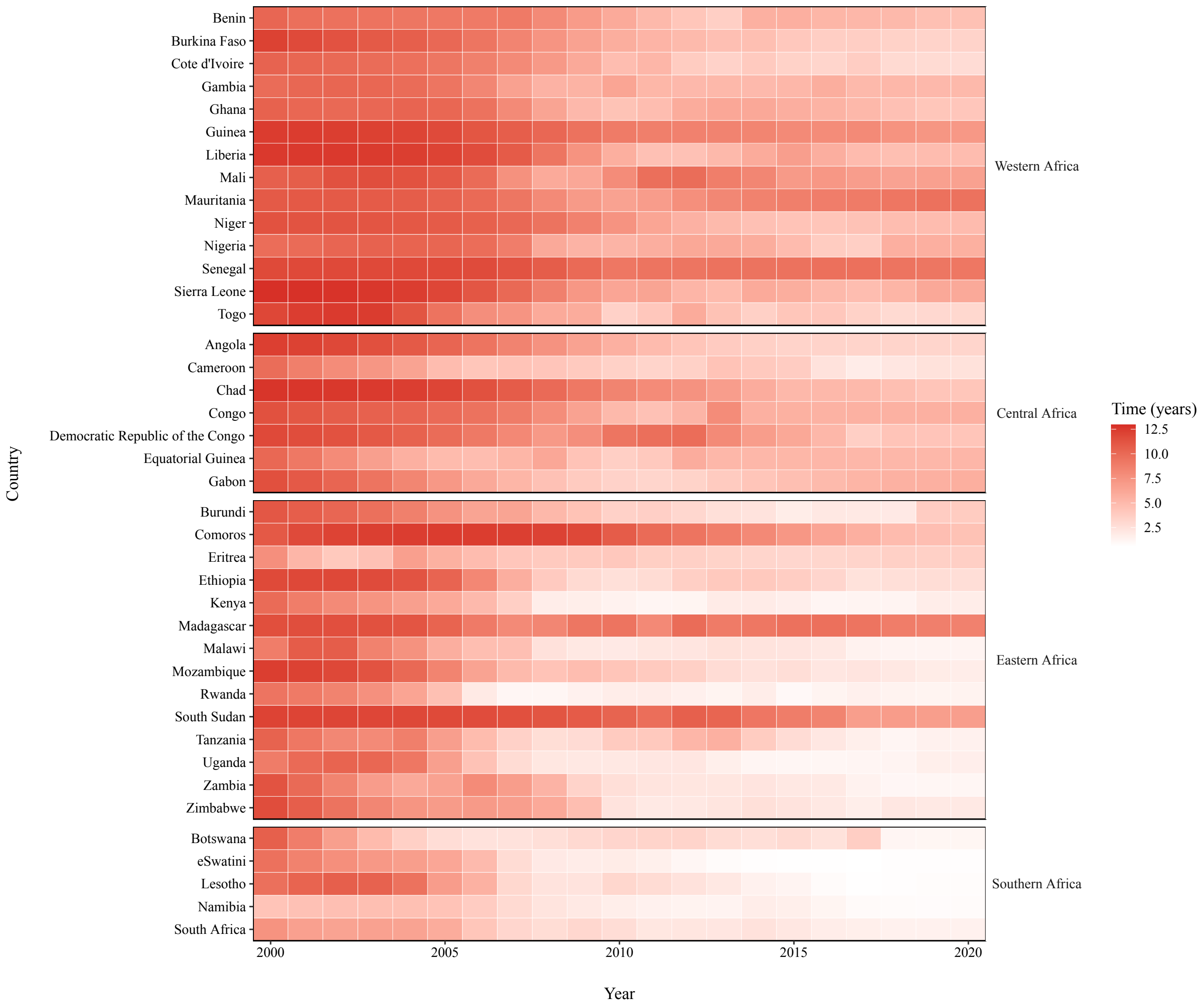

***Figure S3*. National progress in median time to diagnosis or AIDS-related death in sub-Saharan Africa, 2000-2020.**

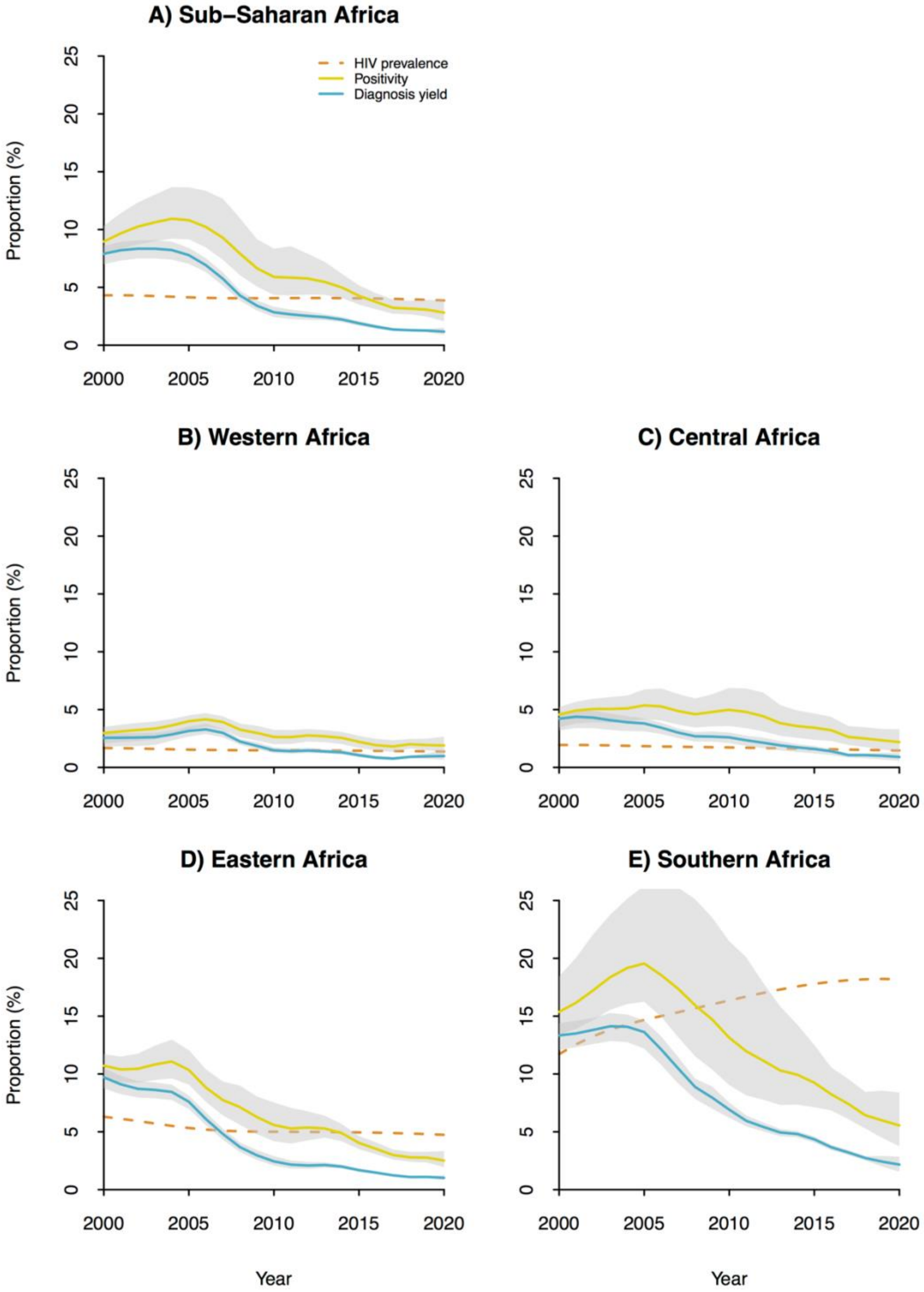

***Figure S4*. Regional trends in positivity and diagnosis yield, 2000-2020.**

The positivity corresponds to the proportion of positive tests among all tests, while diagnosis yield corresponds to the proportion of new diagnoses among all tests. HIV prevalence is expressed among the total population aged 15+ years. The shaded areas correspond to the 95% credible intervals.

Table S1. **Supplemental HIV testing services programme data sources.**

Country	Year	Source
Burundi	2003	Conseil National de Lutte contre le SIDA, Rapport d'activités sur la lutte contre le SIDA et rapport sur les progrès enregistrés vers un accès universel (Burundi), 2015. Available from: https://www.unaids.org/en/dataanalysis/knowyourresponse/countryprogressreports/2015countries - B
	2004	Conseil National de Lutte contre le SIDA, Rapport d'activités sur la lutte contre le SIDA et rapport sur les progrès enregistrés vers un accès universel (Burundi), 2015. Available from: https://www.unaids.org/en/dataanalysis/knowyourresponse/countryprogressreports/2015countries - B
	2005	Conseil National de Lutte contre le SIDA, Rapport d'activités sur la lutte contre le SIDA et rapport sur les progrès enregistrés vers un accès universel (Burundi), 2015. Available from: https://www.unaids.org/en/dataanalysis/knowyourresponse/countryprogressreports/2015countries - B
	2006	Conseil National de Lutte contre le SIDA, Rapport d'activités sur la lutte contre le SIDA et rapport sur les progrès enregistrés vers un accès universel (Burundi), 2015. Available from: https://www.unaids.org/en/dataanalysis/knowyourresponse/countryprogressreports/2015countries - B
	2007	Conseil National de Lutte contre le SIDA, Rapport d'activités sur la lutte contre le SIDA et rapport sur les progrès enregistrés vers un accès universel (Burundi), 2015. Available from: https://www.unaids.org/en/dataanalysis/knowyourresponse/countryprogressreports/2015countries - B
	2008	Conseil National de Lutte contre le SIDA, Rapport d'activités sur la lutte contre le SIDA et rapport sur les progrès enregistrés vers un accès universel (Burundi), 2015. Available from: https://www.unaids.org/en/dataanalysis/knowyourresponse/countryprogressreports/2015countries - B
	2009	Conseil National de Lutte contre le SIDA, Rapport d'activités sur la lutte contre le SIDA et rapport sur les progrès enregistrés vers un accès universel (Burundi), 2015. Available from: https://www.unaids.org/en/dataanalysis/knowyourresponse/countryprogressreports/2015countries - B
Cameroon	2013	Rapport national de suivi de la déclaration politique sur le VIH/SIDA Cameroun – Global AIDS Response Progress (GARP), 2014. Available from: https://www.unaids.org/en/dataanalysis/knowyourresponse/countryprogressreports/2014countries - C
	2014	Ministry of Public Health, Plan d'accélération de la thérapie ARV au Cameroun 2016-2018, 2015.
Comoros	2018	UNAIDS/WHO/UNICEF. Global AIDS Monitoring, 2020. Accessed at: https://aidsinfo.unaids.org/
Congo	2013	Conseil National de Lutte contre le SIDA, Rapport d'activités de la riposte au VIH/SIDA – 2013, 2014. Available from: https://www.unaids.org/en/dataanalysis/knowyourresponse/countryprogressreports/2014countries - C
Equatorial Guinea	2008	Programa nacional de lucha contra el SIDA, Declaracion de compromiso sobre VIH-SIDA, UNGASS, 2010. Available from: https://www.unaids.org/en/dataanalysis/knowyourresponse/countryprogressreports/2010countries - E
	2012	Programa nacional de lucha contra el SIDA, informe national sobre los progresos realizados en la lucha contra el VIH/SIDA Guinea Ecuatorial, 2014. Available from: https://www.unaids.org/en/dataanalysis/knowyourresponse/countryprogressreports/2014countries - E
	2013	Programa nacional de lucha contra el SIDA, informe national sobre los progresos realizados en la lucha contra el VIH/SIDA Guinea Ecuatorial, 2014. Available from: https://www.unaids.org/en/dataanalysis/knowyourresponse/countryprogressreports/2014countries - E
Eritrea	2004	National AIDS and Tuberculosis Control Division (NATCoD), 2012. Available from: https://www.unaids.org/en/dataanalysis/knowyourresponse/countryprogressreports/2012countries - E
	2005	National AIDS and Tuberculosis Control Division (NATCoD), 2012. Available from: https://www.unaids.org/en/dataanalysis/knowyourresponse/countryprogressreports/2012countries - E
	2006	National AIDS and Tuberculosis Control Division (NATCoD), 2012. Available from: https://www.unaids.org/en/dataanalysis/knowyourresponse/countryprogressreports/2012countries - E
	2007	National AIDS and Tuberculosis Control Division (NATCoD), 2012. Available from: https://www.unaids.org/en/dataanalysis/knowyourresponse/countryprogressreports/2012countries - E
	2008	National AIDS and Tuberculosis Control Division (NATCoD), 2012. Available from: https://www.unaids.org/en/dataanalysis/knowyourresponse/countryprogressreports/2012countries - E
	2009	National AIDS and Tuberculosis Control Division (NATCoD), 2012. Available from: https://www.unaids.org/en/dataanalysis/knowyourresponse/countryprogressreports/2012countries - E
	2010	National AIDS and Tuberculosis Control Division (NATCoD), 2012. Available from: https://www.unaids.org/en/dataanalysis/knowyourresponse/countryprogressreports/2012countries - E
	2011	National AIDS and Tuberculosis Control Division (NATCoD), 2012. Available from: https://www.unaids.org/en/dataanalysis/knowyourresponse/countryprogressreports/2012countries - E
	2018	UNAIDS/WHO/UNICEF. Global AIDS Monitoring, 2020. Accessed at: https://aidsinfo.unaids.org/
Gabon	2018	UNAIDS/WHO/UNICEF. Global AIDS Monitoring, 2020. Accessed at: https://aidsinfo.unaids.org/
Gambia	2010	National AIDS Secretariat Office of the President, Country progress report – The Gambia, 2012. Available from: https://www.unaids.org/en/dataanalysis/knowyourresponse/countryprogressreports/2012countries#G
	2011	National AIDS Secretariat Office of the President, Country progress report – The Gambia, 2012. Available from: https://www.unaids.org/en/dataanalysis/knowyourresponse/countryprogressreports/2012countries - G
	2013	National AIDS Secretariat Office of the President, The Gambia Global AIDS Response Progress Report, 2015. Available from: https://www.unaids.org/en/dataanalysis/knowyourresponse/countryprogressreports/2015countries - G

	2014	National AIDS Secretariat Office of the President, The Gambia Global AIDS Response Progress Report, 2015. Available from: https://www.unaids.org/en/dataanalysis/knowyourresponse/countryprogressreports/2015countries - G
Ghana	2011	Ghana AIDS Commission, Country AIDS Response Progress Report – Ghana, 2015. Available from: https://www.unaids.org/en/dataanalysis/knowyourresponse/countryprogressreports/2015countries - G
Guinea	2014	Comité National de Lutte contre le SIDA, Rapport national de la riposte VIH/SIDA 2014 - Progrès 2010-2014, 2015. Available from: https://www.unaids.org/en/dataanalysis/knowyourresponse/countryprogressreports/2015countries - G
	2018	UNAIDS/WHO/UNICEF. Global AIDS Monitoring, 2020. Accessed at: https://aidsinfo.unaids.org/
Lesotho	2005	Lesotho UNGASS Country Report - Status of the national response to the 2001 declaration of commitment on HIV and AIDS, 2010. Available from: https://www.unaids.org/en/dataanalysis/knowyourresponse/countryprogressreports/2010countries - L
	2006	Lesotho UNGASS Country Report - Status of the national response to the 2001 declaration of commitment on HIV and AIDS, 2010. Available from: https://www.unaids.org/en/dataanalysis/knowyourresponse/countryprogressreports/2010countries - L
	2007	Lesotho UNGASS Country Report - Status of the national response to the 2001 declaration of commitment on HIV and AIDS, 2010. Available from: https://www.unaids.org/en/dataanalysis/knowyourresponse/countryprogressreports/2010countries - L
	2008	Lesotho UNGASS Country Report - Status of the national response to the 2001 declaration of commitment on HIV and AIDS, 2010. Available from: https://www.unaids.org/en/dataanalysis/knowyourresponse/countryprogressreports/2010countries - L
	2009	Lesotho UNGASS Country Report - Status of the national response to the 2001 declaration of commitment on HIV and AIDS, 2010. Available from: https://www.unaids.org/en/dataanalysis/knowyourresponse/countryprogressreports/2010countries - L
Liberia	2013	Jacobs GP et al. 2017. Did the 2014 Ebola outbreak in Liberia affect HIV testing, linkage to care and ART initiation?, Public Health Action, 7(S1):S70-S75)
Madagascar	2010	Comité National de Lutte contre le SIDA, Rapport sur la réponse face au VIH et au SIDA à Madagascar 2014, 2015. Available from: https://www.unaids.org/en/dataanalysis/knowyourresponse/countryprogressreports/2015countries - M
	2011	Comité National de Lutte contre le SIDA, Rapport sur la réponse face au VIH et au SIDA à Madagascar 2014, 2015. Available from: https://www.unaids.org/en/dataanalysis/knowyourresponse/countryprogressreports/2015countries - M
	2012	Comité National de Lutte contre le SIDA, Rapport sur la réponse face au VIH et au SIDA à Madagascar 2014, 2015. Available from: https://www.unaids.org/en/dataanalysis/knowyourresponse/countryprogressreports/2015countries - M
	2013	Comité National de Lutte contre le SIDA, Rapport sur la réponse face au VIH et au SIDA à Madagascar 2014, 2015. Available from: https://www.unaids.org/en/dataanalysis/knowyourresponse/countryprogressreports/2015countries - M
	2014	Comité National de Lutte contre le SIDA, Rapport sur la réponse face au VIH et au SIDA à Madagascar 2014, 2015. Available from: https://www.unaids.org/en/dataanalysis/knowyourresponse/countryprogressreports/2015countries - M
	2018	UNAIDS/WHO/UNICEF. Global AIDS Monitoring, 2020. Accessed at: https://aidsinfo.unaids.org/
Mozambique	2013	Maheu-Giroux M, Marsh K, Doyle C, et al. National HIV testing and diagnosis coverage in sub-Saharan Africa: a new modeling tool for estimating the "first 90" from program and survey data. *AIDS* 2019; **33**: S255–S69.
	2014	Maheu-Giroux M, Marsh K, Doyle C, et al. National HIV testing and diagnosis coverage in sub-Saharan Africa: a new modeling tool for estimating the "first 90" from program and survey data. *AIDS* 2019; **33**: S255–S69.
	2016	Maheu-Giroux M, Marsh K, Doyle C, et al. National HIV testing and diagnosis coverage in sub-Saharan Africa: a new modeling tool for estimating the "first 90" from program and survey data. *AIDS* 2019; **33**: S255–S69.
Namibia	2008	Republic of Namibia Ministry of Health and Social Services, United Nations General Assembly Special Session (UNGASS) Country Report - Reporting Period 2008-2009, 2010. Available from: https://www.unaids.org/en/dataanalysis/knowyourresponse/countryprogressreports/2010countries - L
Niger	2018	UNAIDS/WHO/UNICEF. Global AIDS Monitoring, 2020. Accessed at: https://aidsinfo.unaids.org/
Nigeria	2018	UNAIDS/WHO/UNICEF. Global AIDS Monitoring, 2020. Accessed at: https://aidsinfo.unaids.org/
Senegal	2008	Conseil National de Lutte contre le SIDA, Rapport de situation sur la riposte nationale à l'épidémie de VIH/SIDA – Sénégal: 2008-2009, 2010. Available from: https://www.unaids.org/en/dataanalysis/knowyourresponse/countryprogressreports/2010countries - S
	2012	Conseil National de Lutte contre le SIDA, Rapport de situation sur la riposte nationale à l'épidémie de VIH/SIDA – Sénégal: 2012-2013, 2014. Availble from: https://www.unaids.org/en/dataanalysis/knowyourresponse/countryprogressreports/2014countries - S
	2013	Conseil National de Lutte contre le SIDA, Rapport de situation sur la riposte nationale à l'épidémie de VIH/SIDA – Sénégal: 2012-2013, 2014. Available from: https://www.unaids.org/en/dataanalysis/knowyourresponse/countryprogressreports/2014countries - S
Sierra Leone	2012	Sierra Leone National AIDS Response Progress Report 2014, 2014. Available from: https://www.unaids.org/en/dataanalysis/knowyourresponse/countryprogressreports/2014countries - S
South Africa	2010	Johnson L and Dorrington R. 2020. Thembisa version 4.3: A model for evaluating the impact of HIV/AIDS in South Africa. Available at: https://www.thembisa.org/content/filedl/Thembisa4_3report
	2011	Johnson L and Dorrington R. 2020. Thembisa version 4.3: A model for evaluating the impact of HIV/AIDS in South Africa. Available at: https://www.thembisa.org/content/filedl/Thembisa4_3report
	2012	Johnson L and Dorrington R. 2020. Thembisa version 4.3: A model for evaluating the impact of HIV/AIDS in South Africa. Available at: https://www.thembisa.org/content/filedl/Thembisa4_3report

	2013	Johnson L and Dorrington R. 2020. Thembisa version 4.3: A model for evaluating the impact of HIV/AIDS in South Africa. Available at: https://www.thembisa.org/content/filedl/Thembisa4_3report
	2014	Johnson L and Dorrington R. 2020. Thembisa version 4.3: A model for evaluating the impact of HIV/AIDS in South Africa. Available at: https://www.thembisa.org/content/filedl/Thembisa4_3report
	2015	Johnson L and Dorrington R. 2020. Thembisa version 4.3: A model for evaluating the impact of HIV/AIDS in South Africa. Available at: https://www.thembisa.org/content/filedl/Thembisa4_3report
	2016	Johnson L and Dorrington R. 2020. Thembisa version 4.3: A model for evaluating the impact of HIV/AIDS in South Africa. Available at: https://www.thembisa.org/content/filedl/Thembisa4_3report
	2017	Johnson L and Dorrington R. 2020. Thembisa version 4.3: A model for evaluating the impact of HIV/AIDS in South Africa. Available at: https://www.thembisa.org/content/filedl/Thembisa4_3report
	2018	UNAIDS/WHO/UNICEF. Global AIDS Monitoring, 2020. Accessed at: https://aidsinfo.unaids.org/
Togo	2002	Conseil National de Lutte contre le SIDA et les Infections Sexuellement Transmissibles, Suivi de la déclaration d'engagement sur le VIH et le SIDA - Rapport 2010, 2010. Available from: https://www.unaids.org/en/dataanalysis/knowyourresponse/countryprogressreports/2010countries - T
	2003	Conseil National de Lutte contre le SIDA et les Infections Sexuellement Transmissibles, Suivi de la déclaration d'engagement sur le VIH et le SIDA - Rapport 2010, 2010. Available from: https://www.unaids.org/en/dataanalysis/knowyourresponse/countryprogressreports/2010countries - T
	2004	Conseil National de Lutte contre le SIDA et les Infections Sexuellement Transmissibles, Suivi de la déclaration d'engagement sur le VIH et le SIDA - Rapport 2010, 2010. Available from: https://www.unaids.org/en/dataanalysis/knowyourresponse/countryprogressreports/2010countries - T
	2005	Conseil National de Lutte contre le SIDA et les Infections Sexuellement Transmissibles, Suivi de la déclaration d'engagement sur le VIH et le SIDA - Rapport 2010, 2010. Available from: https://www.unaids.org/en/dataanalysis/knowyourresponse/countryprogressreports/2010countries - T
	2006	Conseil National de Lutte contre le SIDA et les Infections Sexuellement Transmissibles, Suivi de la déclaration d'engagement sur le VIH et le SIDA - Rapport 2010, 2010. Available from: https://www.unaids.org/en/dataanalysis/knowyourresponse/countryprogressreports/2010countries - T
	2007	Conseil National de Lutte contre le SIDA et les Infections Sexuellement Transmissibles, Suivi de la déclaration d'engagement sur le VIH et le SIDA - Rapport 2010, 2010. Available from: https://www.unaids.org/en/dataanalysis/knowyourresponse/countryprogressreports/2010countries - T
	2008	Conseil National de Lutte contre le SIDA et les Infections Sexuellement Transmissibles, Suivi de la déclaration d'engagement sur le VIH et le SIDA - Rapport 2010, 2010. Available from: https://www.unaids.org/en/dataanalysis/knowyourresponse/countryprogressreports/2010countries - T
	2009	Conseil National de Lutte contre le SIDA et les Infections Sexuellement Transmissibles, Suivi de la déclaration d'engagement sur le VIH et le SIDA - Rapport 2010, 2010. Available from: https://www.unaids.org/en/dataanalysis/knowyourresponse/countryprogressreports/2010countries - T
	2012	Conseil National de Lutte contre le SIDA et les Infections Sexuellement Transmissibles, Rapport annuel des activités de lutte contre le VIH/SIDA au Togo en 2012, 2013.
	2014	Conseil National de Lutte contre le SIDA et les Infections Sexuellement Transmissibles, Rapport d'activité sur la riposte au VIH/SIDA au Togo, 2015. Available from: https://www.unaids.org/en/dataanalysis/knowyourresponse/countryprogressreports/2015countries - T
	2015	Programme national de lutte contre le SIDA et les IST, Rapport annuel 2015 des activités du PNLS-IST, 2015. Available from: http://www.pnls.tg/rapports/RAPPORT ANNUEL PNLS 2015.pdf
Zambia	2006	National AIDS Council, Zambia country report - Monitoring the declaration of commitment on HIV and AIDS and the universal access, 2010. Available from: https://www.unaids.org/en/dataanalysis/knowyourresponse/countryprogressreports/2010countries - T
	2007	National AIDS Council, Zambia country report - Monitoring the declaration of commitment on HIV and AIDS and the universal access, 2010. Available from: https://www.unaids.org/en/dataanalysis/knowyourresponse/countryprogressreports/2010countries - T
	2008	National AIDS Council, Zambia country report - Monitoring the declaration of commitment on HIV and AIDS and the universal access, 2010. Available from: https://www.unaids.org/en/dataanalysis/knowyourresponse/countryprogressreports/2010countries - T
	2009	National AIDS Council, Zambia country report - Monitoring the declaration of commitment on HIV and AIDS and the universal access, 2010. Available from: https://www.unaids.org/en/dataanalysis/knowyourresponse/countryprogressreports/2010countries - T

Table S2. **GATHER checklist of information that should be included in new reports of global health estimates.**

Item #	Checklist item	Reported on page #
**Objectives and funding**		
1	Define the indicator(s), populations (including age, sex, and geographic entities), and time period(s) for which estimates were made.	3-5
2	List the funding sources for the work.	1
**Data Inputs**		
For all data inputs from multiple sources that are synthesized as part of the study:		
3	Describe how the data were identified and how the data were accessed.	3
4	Specify the inclusion and exclusion criteria. Identify all ad-hoc exclusions.	3
5	Provide information on all included data sources and their main characteristics. For each data source used, report reference information or contact name/institution, population represented, data collection method, year(s) of data collection, sex and age range, diagnostic criteria or measurement method, and sample size, as relevant.	3, Figure 1, Table S1 (appendix pp 6-8)
6	Identify and describe any categories of input data that have potentially important biases (e.g., based on characteristics listed in item 5).	3, 8-9
For data inputs that contribute to the analysis but were not synthesized as part of the study:		
7	Describe and give sources for any other data inputs.	3, Figure 1, Table S1 (appendix pp 6-8)
For all data inputs:		
8	Provide all data inputs in a file format from which data can be efficiently extracted (e.g., a spreadsheet rather than a PDF), including all relevant meta-data listed in item 5. For any data inputs that cannot be shared because of ethical or legal reasons, such as third-party ownership, provide a contact name or the name of the institution that retains the right to the data.	9 (links provided in the Data sharing section)
**Data analysis**		
9	Provide a conceptual overview of the data analysis method. A diagram may be helpful.	3 (see also Maheu-Giroux et al. 2019)
10	Provide a detailed description of all steps of the analysis, including mathematical formulae. This description should cover, as relevant, data cleaning, data pre-processing, data adjustments and weighting of data sources, and mathematical or statistical model(s).	3-5, appendix p 1 (see also Maheu-Giroux et al. 2019)
11	Describe how candidate models were evaluated and how the final model(s) were selected.	3 (see also Maheu-Giroux et al. 2019)
12	Provide the results of an evaluation of model performance, if done, as well as the results of any relevant sensitivity analysis.	appendix pp 17-58 (see also Maheu-Giroux et al. 2019)
13	Describe methods for calculating uncertainty of the estimates. State which sources of uncertainty were, and were not, accounted for in the uncertainty analysis.	4-5 (see also Maheu-Giroux et al. 2019)
14	State how analytic or statistical source code used to generate estimates can be accessed.	4, 9
**Results and Discussion**		
15	Provide published estimates in a file format from which data can be efficiently extracted.	Table 1, Tables S3-S8 (appendix pp 11-16)
16	Report a quantitative measure of the uncertainty of the estimates (e.g. uncertainty intervals).	Tables and Figures
17	Interpret results in light of existing evidence. If updating a previous set of estimates, describe the reasons for changes in estimates.	7-9
18	Discuss limitations of the estimates. Include a discussion of any modelling assumptions or data limitations that affect interpretation of the estimates.	7-9

Maheu-Giroux M, Marsh K, Doyle C, Godin A, Lanièce Delaunay C, Jonhson LF, Jhan A, Abo K, Mbofana F, Boily MC, Buckeridge DL, Hankins C, and Eaton JW (2019). National HIV testing and diagnosis coverage in sub-Saharan Africa: a new modeling tool for estimating the "first 90" from program and survey data. AIDS 2019 33(Suppl. 3):S255-S259. doi: 10.1097/QAD.0000000000002386

***Table S3*. Number of undiagnosed people living with HIV by sex and age stratification and by sub-Saharan African region in 2020 (in 1,000s).**

Sex	Age group (years)	Sub-Saharan Africa		Western Africa		Central Africa		Eastern Africa		Southern Africa	
Men	15-24	346	(318 to 366)	89	(85 to 91)	33	(30 to 35)	163	(150 to 172)	61	(51 to 69)
	25-34	597	(514 to 673)	156	(143 to 169)	53	(40 to 62)	258	(227 to 287)	130	(100 to 156)
	35-49	701	(611 to 788)	211	(191 to 230)	57	(45 to 68)	296	(265 to 327)	136	(104 to 169)
	50+	264	(231 to 299)	91	(81 to 97)	18	(14 to 21)	109	(96 to 125)	46	(36 to 58)
Women	15-24	563	(492 to 621)	124	(119 to 129)	79	(63 to 91)	222	(200 to 240)	138	(108 to 164)
	25-34	557	(459 to 652)	173	(154 to 188)	92	(66 to 123)	196	(170 to 224)	94	(65 to 125)
	35-49	581	(507 to 664)	166	(156 to 179)	100	(78 to 123)	199	(179 to 221)	115	(87 to 145)
	50+	234	(202 to 272)	54	(51 to 59)	39	(31 to 46)	77	(71 to 87)	64	(48 to 83)

Numbers in parentheses correspond to 95% credible intervals.
Some small discrepancies could be observed between sub-Saharan Africa estimate and the sum of Western, Central, Eastern, and Southern regions estimates because each region-specific estimate is based on independent draws of posterior estimates.

Table S4. **Progress in HIV testing related outcomes in sub-Saharan Africa stratified by sex and age group, 2000-2020.**

Outcome	Year	Overall	Men	Women	15-24 years-old	25-34 years-old	35-49 years-old	50+ years-old
Proportion of individuals having ever been tested for HIV among overall population (%)	2000	3·6 (3·0 to 4·4)	3·3 (2·6 to 4·0)	4·0 (3·3 to 4·9)	3·2 (2·6 to 3·9)	5·1 (4·2 to 6·2)	3·5 (2·9 to 4·3)	2·6 (2·2 to 3·3)
	2005	11 (10 to 12)	9·9 (9·0 to 11)	13 (12 to 14)	8·8 (7·9 to 9·9)	15 (14 to 17)	12 (11 to 13)	9·1 (8·4 to 10)
	2010	30 (29 to 32)	27 (26 to 29)	33 (32 to 35)	25 (23 to 26)	39 (37 to 41)	33 (31 to 34)	26 (25 to 28)
	2015	41 (40 to 42)	37 (36 to 38)	46 (45 to 47)	29 (28 to 31)	52 (51 to 53)	49 (48 to 50)	40 (38 to 42)
	2020	51 (49 to 54)	46 (43 to 49)	56 (54 to 60)	35 (32 to 39)	61 (59 to 64)	62 (61 to 65)	54 (51 to 57)
Proportion of PLHIV who know their HIV status (%)	2000	5·7 (4·6 to 7·0)	4·6 (3·7 to 5·8)	6·4 (5·1 to 7·9)	4·9 (3·8 to 6·1)	7·2 (5·7 to 8·8)	4·9 (4·0 to 6·1)	3·5 (2·8 to 4·3)
	2005	20 (18 to 22)	16 (14 to 18)	22 (20 to 25)	14 (12 to 16)	24 (21 to 27)	19 (17 to 22)	15 (14 to 17)
	2010	53 (50 to 55)	46 (43 to 48)	57 (54 to 59)	38 (35 to 41)	58 (54 to 60)	55 (52 to 57)	50 (48 to 53)
	2015	71 (69 to 73)	64 (62 to 66)	75 (73 to 77)	51 (49 to 53)	72 (69 to 74)	76 (74 to 78)	75 (73 to 77)
	2020	84 (82 to 86)	79 (76 to 81)	87 (85 to 89)	65 (62 to 69)	81 (79 to 84)	88 (86 to 89)	90 (88 to 91)
Time to diagnosis or AIDS-related death, median (year)	2000	9·6 (9·1 to 10)	9·7 (9·2 to 10)	9·5 (8·9 to 10)	11 (10 to 12)	9·1 (8·5 to 9·5)	8·2 (7·9 to 8·5)	7·6 (7·5 to 7·8)
	2005	7·2 (6·3 to 8·0)	7·7 (6·8 to 8·4)	6·9 (5·9 to 7·8)	7·7 (6·6 to 8·7)	6·8 (5·9 to 7·6)	6·8 (6·3 to 7·3)	6·6 (6·2 to 6·9)
	2010	3·6 (3·2 to 4·1)	4·3 (3·8 to 4·7)	3·2 (2·8 to 3·7)	3·7 (3·2 to 4·3)	3·1 (2·7 to 3·6)	4·0 (3·6 to 4·4)	4·2 (3·9 to 4·6)
	2015	3·0 (2·7 to 3·4)	3·7 (3·4 to 4·2)	2·6 (2·2 to 2·9)	3·2 (2·8 to 3·6)	2·5 (2·2 to 2·9)	3·3 (3·0 to 3·7)	3·5 (3·3 to 3·8)
	2020	2·6 (1·8 to 3·5)	3·3 (2·4 to 4·2)	2·2 (1·5 to 3·0)	2·8 (2·0 to 3·8)	2·2 (1·5 to 3·0)	2·8 (2·0 to 3·6)	2·9 (2·2 to 3·6)
Probability of getting tested within 1 year following infection (%)	2000	2·6 (2·1 to 3·3)	2·1 (1·6 to 2·6)	3·0 (2·3 to 3·8)	2·6 (2·0 to 3·3)	3·2 (2·5 to 4·0)	2·1 (1·6 to 2·6)	1·4 (1·1 to 1·8)
	2005	5·9 (4·5 to 7·7)	4·6 (3·5 to 6·1)	6·7 (5·0 to 8·8)	5·9 (4·5 to 7·8)	6·9 (5·3 to 9·1)	4·5 (3·4 to 5·9)	3·5 (2·7 to 4·6)
	2010	21 (18 to 25)	16 (13 to 19)	25 (21 to 29)	21 (18 to 25)	26 (22 to 31)	16 (13 to 19)	12 (9·9 to 15)
	2015	26 (23 to 30)	19 (16 to 22)	31 (27 to 35)	26 (23 to 29)	32 (28 to 37)	20 (17 to 23)	16 (14 to 19)
	2020	33 (23 to 46)	25 (17 to 36)	39 (27 to 53)	32 (23 to 45)	40 (28 to 54)	27 (17 to 39)	23 (15 to 33)
Probability of getting tested before reaching a CD4 count lower than 350 cells per µL (%)	2000	19 (16 to 23)	16 (13 to 19)	22 (18 to 26)	24 (20 to 28)	20 (16 to 24)	12 (10 to 15)	7·2 (5·8 to 8·9)
	2005	35 (29 to 42)	30 (24 to 36)	38 (32 to 45)	42 (35 to 49)	36 (30 to 43)	24 (19 to 29)	16 (13 to 20)
	2010	63 (58 to 66)	56 (51 to 60)	67 (63 to 71)	68 (64 to 72)	67 (63 to 71)	50 (45 to 55)	38 (33 to 42)
	2015	67 (63 to 70)	60 (55 to 63)	72 (68 to 75)	71 (67 to 75)	72 (68 to 76)	56 (51 to 60)	44 (40 to 48)
	2020	71 (62 to 79)	64 (54 to 73)	76 (67 to 83)	74 (66 to 82)	76 (67 to 83)	62 (51 to 71)	52 (41 to 62)
Positivity (% of positive test among all tests)	2000	9·0 (7·7 to 10)	6·6 (5·7 to 7·5)	11 (9·3 to 13)	5·8 (5·1 to 6·6)	13 (11 to 15)	10 (9·0 to 12)	3·4 (2·9 to 3·9)
	2005	11 (9·2 to 14)	7·9 (6·9 to 9·3)	13 (11 to 17)	5·6 (4·8 to 6·8)	16 (13 to 21)	15 (13 to 18)	5·4 (4·8 to 6·2)
	2010	5·9 (4·3 to 8·3)	4·3 (3·3 to 5·8)	6·9 (5·0 to 10)	3·1 (2·4 to 4·2)	7·5 (5·2 to 11)	9·5 (7·3 to 13)	3·8 (3·0 to 4·8)
	2015	4·3 (3·5 to 5·2)	3·6 (3·0 to 4·3)	4·6 (3·8 to 5·8)	2·5 (2·1 to 2·9)	4·8 (3·7 to 6·1)	6·9 (5·7 to 8·5)	3·4 (2·7 to 4·2)
	2020	2·8 (2·1 to 3·9)	2·6 (1·9 to 3·4)	3·0 (2·2 to 4·3)	1·6 (1·3 to 2·0)	3·0 (2·2 to 4·1)	4·6 (3·3 to 6·7)	2·8 (1·9 to 4·3)
Diagnosis yield (% of new diagnoses among all tests)	2000	7·9 (7·0 to 8·6)	6·0 (5·2 to 6·6)	9·4 (8·3 to 10)	5·3 (4·7 to 5·8)	11 (10 to 12)	9·4 (8·2 to 10)	3·1 (2·7 to 3·5)
	2005	7·8 (7·0 to 8·4)	6·2 (5·6 to 6·7)	8·9 (8·0 to 9·7)	4·4 (4·0 to 4·8)	11 (9·4 to 12)	11 (10 to 12)	4·5 (4·0 to 5·0)
	2010	2·8 (2·4 to 3·3)	2·5 (2·2 to 3·0)	3·0 (2·6 to 3·6)	1·8 (1·6 to 2·1)	3·0 (2·5 to 3·5)	4·9 (4·2 to 5·7)	2·5 (2·1 to 2·9)
	2015	1·9 (1·7 to 2·1)	1·9 (1·6 to 2·1)	1·9 (1·7 to 2·1)	1·5 (1·3 to 1·6)	1·8 (1·6 to 2·0)	2·9 (2·5 to 3·3)	1·8 (1·6 to 2·1)
	2020	1·2 (0·9 to 1·5)	1·1 (0·9 to 1·5)	1·2 (0·9 to 1·6)	1·0 (0·8 to 1·2)	1·1 (0·8 to 1·4)	1·7 (1·2 to 2·3)	1·2 (0·8 to 1·7)
Proportion of new HIV diagnoses among all positive tests (%)	2000	89 (77 to 96)	91 (83 to 97)	88 (74 to 96)	92 (82 to 97)	87 (73 to 95)	91 (80 to 97)	92 (83 to 97)
	2005	72 (55 to 86)	79 (65 to 89)	69 (51 to 84)	80 (65 to 91)	66 (48 to 82)	76 (60 to 88)	84 (72 to 92)
	2010	48 (33 to 65)	59 (44 to 73)	44 (29 to 62)	59 (43 to 75)	39 (25 to 58)	52 (38 to 67)	66 (51 to 79)
	2015	44 (37 to 53)	52 (44 to 60)	41 (33 to 50)	60 (52 to 68)	37 (29 to 47)	43 (34 to 51)	55 (43 to 65)
	2020	42 (30 to 55)	45 (34 to 56)	40 (28 to 55)	61 (50 to 73)	36 (25 to 49)	37 (25 to 50)	42 (28 to 59)

PLHIV: people living with HIV; HTS: HIV testing services.
Numbers in parentheses correspond to 95% credible intervals.

***Table S5*. Progress in HIV testing related outcomes in Western Africa stratified by sex and age group, 2000-2020.**

Outcome	Year	Overall	Men	Women	15-24 years-old	25-34 years-old	35-49 years-old	50+ years-old
Proportion of individuals having ever been tested for HIV among overall population (%)	2000	3·1 (2·6 to 3·8)	3·1 (2·5 to 3·7)	3·2 (2·6 to 3·8)	2·7 (2·2 to 3·2)	4·7 (3·9 to 5·8)	2·8 (2·3 to 3·4)	2·1 (1·7 to 2·6)
	2005	7·2 (6·6 to 7·8)	6·9 (6·2 to 7·6)	7·4 (6·8 to 8·1)	5·0 (4·4 to 5·6)	11 (9·7 to 12)	8·1 (7·5 to 8·7)	5·5 (5·0 to 6·0)
	2010	19 (18 to 19)	17 (16 to 18)	20 (19 to 21)	13 (12 to 14)	26 (25 to 27)	21 (20 to 22)	15 (14 to 16)
	2015	28 (27 to 29)	24 (23 to 25)	33 (31 to 34)	18 (17 to 20)	37 (36 to 39)	35 (34 to 36)	26 (24 to 27)
	2020	36 (34 to 39)	30 (28 to 32)	42 (40 to 45)	21 (18 to 24)	45 (43 to 48)	47 (46 to 49)	37 (35 to 40)
Proportion of PLHIV who know their HIV status (%)	2000	4·0 (2·8 to 5·2)	3·9 (2·7 to 5·0)	4·2 (2·9 to 5·4)	3·0 (2·0 to 3·8)	5·4 (3·7 to 6·9)	3·5 (2·5 to 4·5)	2·7 (1·9 to 3·5)
	2005	10 (7·7 to 12)	9·2 (6·8 to 11)	11 (8·3 to 13)	5·4 (4·0 to 6·7)	12 (8·5 to 14)	11 (8·2 to 12)	9·7 (8·1 to 11)
	2010	33 (28 to 35)	28 (24 to 31)	36 (31 to 39)	19 (16 to 22)	35 (28 to 38)	34 (31 to 37)	37 (34 to 38)
	2015	52 (48 to 55)	43 (39 to 46)	58 (54 to 60)	32 (29 to 34)	48 (42 to 52)	57 (54 to 59)	61 (59 to 63)
	2020	67 (65 to 69)	55 (52 to 59)	74 (72 to 76)	42 (40 to 44)	58 (55 to 62)	72 (70 to 74)	79 (77 to 81)
Time to diagnosis or AIDS-related death, median (year)	2000	11 (10 to 11)	10 (9·7 to 11)	11 (10 to 11)	12 (12 to 13)	10 (9·5 to 11)	9·0 (8·8 to 9·3)	7·9 (7·8 to 8·1)
	2005	10 (9·4 to 11)	10 (9·4 to 11)	10 (9·5 to 11)	12 (11 to 13)	9·9 (9·1 to 11)	8·8 (8·5 to 9·1)	7·8 (7·5 to 7·9)
	2010	5·5 (4·9 to 6·5)	6·1 (5·4 to 7·0)	5·1 (4·5 to 6·1)	6·2 (5·4 to 7·4)	4·6 (3·9 to 5·7)	5·8 (5·3 to 6·4)	5·7 (5·3 to 6·1)
	2015	5·2 (4·5 to 6·0)	6·0 (5·2 to 6·8)	4·6 (3·9 to 5·4)	6·0 (5·2 to 7·0)	4·3 (3·6 to 5·3)	5·3 (4·7 to 5·9)	5·2 (4·8 to 5·6)
	2020	5·4 (4·1 to 6·5)	6·2 (4·8 to 7·4)	4·8 (3·6 to 6·0)	6·4 (4·9 to 7·8)	4·5 (3·3 to 5·8)	5·1 (4·1 to 6·1)	5·1 (4·3 to 5·7)
Probability of getting tested within 1 year following infection (%)	2000	1·6 (1·1 to 2·1)	1·5 (1·0 to 2·0)	1·7 (1·2 to 2·2)	1·5 (1·0 to 2·0)	2·2 (1·5 to 2·9)	1·2 (0·8 to 1·5)	1·0 (0·7 to 1·3)
	2005	1·8 (1·1 to 2·6)	1·6 (1·0 to 2·3)	1·9 (1·2 to 2·8)	1·7 (1·1 to 2·4)	2·3 (1·5 to 3·3)	1·3 (0·8 to 1·9)	1·1 (0·7 to 1·6)
	2010	11 (7·7 to 14)	9·0 (6·2 to 12)	13 (8·8 to 17)	9·4 (6·3 to 12)	16 (11 to 21)	8·1 (5·6 to 11)	6·2 (4·4 to 8·0)
	2015	13 (9·3 to 17)	9·8 (6·9 to 13)	16 (11 to 20)	10 (7·3 to 14)	19 (13 to 25)	10 (7·3 to 13)	7·8 (5·8 to 10)
	2020	12 (7·0 to 19)	8·4 (4·8 to 14)	15 (8·7 to 23)	9·1 (5·3 to 15)	17 (10 to 28)	9·8 (5·7 to 16)	7·8 (4·8 to 13)
Probability of getting tested before reaching a CD4 count lower than 350 cells per µL (%)	2000	13 (9·3 to 16)	12 (8·7 to 15)	14 (9·6 to 17)	17 (12 to 21)	15 (10 to 18)	7·6 (5·4 to 9·5)	5·4 (3·8 to 6·8)
	2005	14 (9·2 to 19)	13 (8·3 to 17)	15 (9·9 to 20)	18 (12 to 24)	15 (10 to 21)	8·3 (5·5 to 12)	6·0 (3·9 to 8·5)
	2010	45 (36 to 51)	40 (31 to 46)	49 (40 to 55)	50 (41 to 56)	52 (43 to 59)	33 (25 to 38)	23 (18 to 28)
	2015	47 (38 to 53)	40 (32 to 47)	52 (43 to 59)	50 (41 to 58)	55 (45 to 62)	36 (28 to 42)	26 (20 to 31)
	2020	44 (32 to 56)	37 (25 to 49)	49 (37 to 61)	45 (33 to 58)	52 (39 to 65)	35 (24 to 47)	25 (16 to 35)
Positivity (% of positive test among all tests)	2000	3·0 (2·1 to 3·5)	2·5 (1·7 to 2·9)	3·5 (2·4 to 4·1)	1·4 (1·0 to 1·6)	4·2 (2·9 to 5·0)	4·1 (2·9 to 4·8)	1·8 (1·3 to 2·1)
	2005	4·0 (3·3 to 4·5)	2·9 (2·4 to 3·4)	4·9 (4·1 to 5·5)	1·3 (1·0 to 1·6)	4·8 (3·7 to 5·6)	7·0 (6·1 to 7·9)	4·5 (3·8 to 5·4)
	2010	2·6 (2·0 to 3·2)	2·0 (1·6 to 2·5)	3·0 (2·3 to 3·8)	1·1 (0·9 to 1·3)	2·8 (2·0 to 3·7)	4·6 (3·7 to 5·6)	2·7 (2·2 to 3·3)
	2015	2·2 (1·7 to 2·7)	1·9 (1·4 to 2·3)	2·4 (1·8 to 3·0)	1·0 (0·8 to 1·2)	2·1 (1·5 to 2·6)	4·0 (3·1 to 4·9)	2·7 (2·1 to 3·4)
	2020	1·9 (1·3 to 2·7)	1·7 (1·3 to 2·4)	2·0 (1·4 to 2·9)	0·9 (0·7 to 1·3)	1·6 (1·2 to 2·2)	3·2 (2·3 to 4·6)	2·7 (1·8 to 4·0)
Diagnosis yield (% of new diagnoses among all tests)	2000	2·6 (1·8 to 2·9)	2·1 (1·5 to 2·5)	2·9 (2·1 to 3·4)	1·2 (0·9 to 1·4)	3·5 (2·4 to 4·0)	3·6 (2·6 to 4·2)	1·7 (1·2 to 1·9)
	2005	3·2 (2·7 to 3·6)	2·3 (1·9 to 2·6)	3·9 (3·3 to 4·4)	1·1 (0·9 to 1·3)	3·5 (2·7 to 4·0)	5·6 (4·9 to 6·6)	4·0 (3·4 to 5·0)
	2010	1·5 (1·2 to 1·7)	1·3 (1·1 to 1·5)	1·6 (1·3 to 1·9)	0·7 (0·6 to 0·8)	1·3 (1·1 to 1·6)	2·8 (2·4 to 3·3)	1·9 (1·5 to 2·3)
	2015	1·1 (0·9 to 1·3)	1·0 (0·9 to 1·2)	1·1 (0·9 to 1·3)	0·6 (0·5 to 0·7)	0·9 (0·7 to 1·0)	1·9 (1·6 to 2·4)	1·4 (1·1 to 1·8)
	2020	1·0 (0·7 to 1·5)	1·0 (0·7 to 1·4)	1·0 (0·6 to 1·6)	0·6 (0·4 to 1·0)	0·7 (0·5 to 1·0)	1·7 (1·1 to 2·6)	1·5 (0·9 to 2·4)
Proportion of new HIV diagnoses among all positive tests (%)	2000	86 (79 to 94)	87 (80 to 95)	85 (78 to 94)	91 (86 to 96)	83 (74 to 93)	88 (83 to 95)	91 (87 to 96)
	2005	79 (71 to 90)	80 (72 to 91)	79 (71 to 89)	86 (79 to 93)	74 (65 to 87)	81 (73 to 90)	89 (84 to 94)
	2010	56 (46 to 73)	65 (53 to 81)	53 (43 to 69)	67 (59 to 81)	47 (38 to 67)	61 (49 to 75)	70 (57 to 82)
	2015	48 (38 to 61)	54 (44 to 68)	45 (35 to 58)	60 (51 to 71)	42 (32 to 57)	49 (38 to 62)	53 (41 to 65)
	2020	52 (38 to 68)	56 (44 to 70)	50 (35 to 68)	68 (55 to 81)	44 (31 to 60)	53 (38 to 69)	55 (39 to 72)

PLHIV: people living with HIV; HTS: HIV testing services.
Numbers in parentheses correspond to 95% credible intervals.

Table S6. Progress in HIV testing related outcomes in Central Africa stratified by sex and age group, 2000-2020.

Outcome	Year	Overall	Men	Women	15-24 years-old	25-34 years-old	35-49 years-old	50+ years-old
Proportion of individuals having ever been tested for HIV among overall population (%)	2000	1·9 (1·3 to 2·6)	1·7 (1·2 to 2·3)	2·0 (1·4 to 2·8)	1·6 (1·1 to 2·2)	2·5 (1·8 to 3·5)	1·9 (1·3 to 2·5)	1·5 (1·0 to 2·0)
	2005	7·8 (6·6 to 9·2)	7·0 (5·8 to 8·4)	8·6 (7·3 to 10)	6·0 (5·0 to 7·3)	11 (9·0 to 13)	8·5 (7·3 to 10)	6·6 (5·6 to 7·8)
	2010	19 (17 to 22)	17 (15 to 20)	21 (19 to 23)	13 (12 to 16)	26 (23 to 29)	23 (21 to 25)	18 (16 to 20)
	2015	29 (28 to 30)	26 (24 to 27)	32 (31 to 33)	18 (17 to 19)	37 (35 to 39)	37 (35 to 38)	29 (26 to 32)
	2020	42 (39 to 48)	39 (34 to 44)	47 (42 to 53)	26 (22 to 33)	52 (47 to 59)	54 (50 to 59)	46 (41 to 51)
Proportion of PLHIV who know their HIV status (%)	2000	3·2 (2·0 to 4·6)	3·1 (2·0 to 4·5)	3·3 (2·1 to 4·8)	2·7 (1·7 to 4·0)	3·9 (2·5 to 5·6)	2·9 (1·8 to 4·2)	2·4 (1·5 to 3·4)
	2005	15 (12 to 18)	15 (12 to 18)	16 (12 to 18)	11 (8·8 to 14)	18 (14 to 22)	15 (12 to 17)	13 (10 to 15)
	2010	37 (32 to 41)	36 (32 to 40)	38 (32 to 42)	21 (17 to 25)	40 (34 to 45)	41 (36 to 44)	38 (35 to 42)
	2015	53 (47 to 56)	50 (45 to 54)	54 (48 to 58)	30 (25 to 33)	53 (45 to 57)	59 (54 to 63)	59 (55 to 63)
	2020	70 (64 to 76)	68 (63 to 74)	71 (65 to 78)	45 (38 to 54)	67 (58 to 76)	76 (71 to 81)	79 (76 to 83)
Time to diagnosis or AIDS-related death, median (year)	2000	11 (10 to 12)	11 (10 to 11)	11 (10 to 12)	13 (12 to 14)	10 (9·7 to 11)	9·0 (8·7 to 9·3)	7·9 (7·7 to 8·1)
	2005	8·2 (7·1 to 9·3)	8·2 (7·0 to 9·2)	8·3 (7·1 to 9·4)	9·3 (7·9 to 11)	7·6 (6·5 to 8·7)	7·4 (6·7 to 8·0)	6·7 (6·2 to 7·2)
	2010	6·1 (5·2 to 7·3)	6·1 (5·2 to 7·2)	6·0 (5·1 to 7·3)	6·8 (5·9 to 8·3)	5·3 (4·4 to 6·6)	5·8 (5·0 to 6·6)	5·6 (5·0 to 6·2)
	2015	4·7 (3·9 to 6·0)	5·2 (4·4 to 6·5)	4·5 (3·7 to 5·8)	5·2 (4·3 to 6·6)	4·0 (3·1 to 5·4)	4·8 (4·0 to 5·9)	4·9 (4·2 to 5·6)
	2020	3·9 (2·2 to 6·0)	4·6 (2·8 to 6·5)	3·6 (1·9 to 5·7)	4·3 (2·4 to 6·6)	3·2 (1·7 to 5·5)	4·0 (2·4 to 5·8)	4·1 (2·6 to 5·4)
Probability of getting tested within 1 year following infection (%)	2000	1·5 (1·0 to 2·1)	1·3 (0·9 to 1·9)	1·5 (1·0 to 2·2)	1·5 (0·9 to 2·1)	1·8 (1·2 to 2·6)	1·2 (0·8 to 1·7)	1·0 (0·7 to 1·5)
	2005	5·1 (3·6 to 7·1)	4·6 (3·2 to 6·6)	5·3 (3·7 to 7·4)	4·9 (3·5 to 6·8)	6·2 (4·3 to 8·8)	4·1 (2·8 to 5·7)	3·6 (2·5 to 4·9)
	2010	9·3 (6·5 to 13)	8·1 (5·7 to 12)	9·9 (6·8 to 14)	8·5 (5·9 to 12)	12 (8·1 to 18)	7·5 (5·1 to 11)	6·2 (4·3 to 8·8)
	2015	12 (7·8 to 18)	9·7 (6·3 to 14)	13 (8·4 to 20)	11 (6·9 to 16)	16 (9·9 to 24)	9·7 (6·2 to 15)	8·0 (5·3 to 13)
	2020	16 (7·6 to 34)	13 (6·4 to 26)	17 (8·3 to 37)	14 (7·0 to 30)	20 (9·7 to 42)	13 (6·3 to 30)	11 (5·6 to 25)
Probability of getting tested before reaching a CD4 count lower than 350 cells per µL (%)	2000	12 (8·3 to 17)	11 (7·8 to 16)	12 (8·5 to 17)	15 (10 to 20)	13 (8·6 to 17)	7·7 (5·3 to 11)	5·6 (3·8 to 7·7)
	2005	30 (22 to 38)	29 (22 to 37)	30 (23 to 38)	34 (26 to 42)	32 (24 to 41)	21 (15 to 27)	16 (12 to 21)
	2010	43 (34 to 51)	41 (32 to 49)	44 (34 to 52)	47 (37 to 54)	48 (37 to 56)	33 (25 to 41)	24 (18 to 31)
	2015	52 (41 to 60)	47 (36 to 56)	54 (43 to 62)	56 (45 to 63)	57 (45 to 66)	40 (29 to 49)	30 (21 to 38)
	2020	59 (39 to 75)	52 (34 to 70)	62 (41 to 78)	63 (43 to 78)	64 (42 to 81)	48 (28 to 67)	37 (21 to 56)
Positivity (% of positive test among all tests)	2000	4·6 (3·5 to 5·2)	3·1 (2·3 to 3·6)	5·8 (4·4 to 6·5)	3·1 (2·4 to 3·6)	6·6 (5·0 to 7·6)	5·1 (3·8 to 5·9)	2·1 (1·6 to 2·4)
	2005	5·4 (4·2 to 6·7)	3·7 (2·9 to 4·5)	6·7 (5·2 to 8·5)	2·9 (2·3 to 3·7)	7·8 (6·1 to 10)	6·8 (5·4 to 8·2)	2·9 (2·4 to 3·4)
	2010	5·0 (3·6 to 6·9)	3·4 (2·6 to 4·4)	6·1 (4·3 to 8·6)	2·3 (1·8 to 3·1)	6·5 (4·3 to 9·6)	7·3 (5·4 to 9·6)	3·2 (2·5 to 4·1)
	2015	3·4 (2·4 to 4·7)	2·3 (1·7 to 3·1)	4·2 (2·8 to 5·9)	1·6 (1·2 to 2·1)	3·9 (2·5 to 5·9)	5·6 (3·8 to 7·4)	2·8 (2·0 to 3·7)
	2020	2·2 (1·4 to 3·3)	1·5 (1·0 to 2·2)	2·7 (1·7 to 4·1)	1·1 (0·8 to 1·4)	2·3 (1·4 to 3·7)	3·5 (2·1 to 5·3)	2·1 (1·3 to 3·3)
Diagnosis yield (% of new diagnoses among all tests)	2000	4·2 (3·2 to 4·7)	2·8 (2·1 to 3·3)	5·3 (4·1 to 5·9)	2·9 (2·3 to 3·3)	6·0 (4·6 to 6·8)	4·8 (3·6 to 5·4)	2·0 (1·5 to 2·3)
	2005	3·8 (3·1 to 4·3)	2·7 (2·2 to 3·1)	4·6 (3·8 to 5·2)	2·2 (1·8 to 2·5)	5·2 (4·2 to 5·8)	5·1 (4·2 to 5·7)	2·4 (2·0 to 2·7)
	2010	2·6 (2·1 to 3·0)	2·0 (1·7 to 2·3)	3·0 (2·4 to 3·5)	1·5 (1·2 to 1·7)	2·9 (2·2 to 3·5)	3·9 (3·2 to 4·7)	2·1 (1·7 to 2·6)
	2015	1·6 (1·2 to 1·8)	1·1 (0·9 to 1·3)	1·9 (1·4 to 2·3)	1·0 (0·8 to 1·1)	1·6 (1·1 to 1·9)	2·4 (1·8 to 3·1)	1·5 (1·1 to 1·9)
	2020	0·9 (0·6 to 1·3)	0·7 (0·4 to 1·0)	1·1 (0·7 to 1·5)	0·7 (0·5 to 0·9)	0·9 (0·5 to 1·2)	1·3 (0·8 to 2·1)	0·9 (0·5 to 1·5)
Proportion of new HIV diagnoses among all positive tests (%)	2000	93 (85 to 97)	93 (86 to 97)	92 (85 to 97)	94 (87 to 98)	91 (83 to 96)	93 (87 to 97)	95 (90 to 98)
	2005	71 (55 to 86)	75 (59 to 88)	70 (53 to 85)	76 (60 to 89)	66 (49 to 83)	75 (61 to 88)	82 (70 to 92)
	2010	52 (36 to 71)	59 (43 to 77)	49 (34 to 70)	66 (49 to 83)	44 (29 to 66)	54 (39 to 72)	66 (49 to 80)
	2015	46 (33 to 59)	49 (36 to 60)	45 (32 to 59)	65 (50 to 76)	41 (27 to 57)	44 (31 to 55)	53 (39 to 62)
	2020	42 (24 to 57)	47 (30 to 61)	40 (23 to 55)	62 (42 to 75)	38 (20 to 55)	38 (23 to 52)	43 (27 to 58)

PLHIV: people living with HIV; HTS: HIV testing services.
Numbers in parentheses correspond to 95% credible intervals.

***Table S7*. Progress in HIV testing related outcomes in Eastern Africa stratified by sex and age group, 2000-2020.**

Outcome	Year	Overall	Men	Women	15-24 years-old	25-34 years-old	35-49 years-old	50+ years-old
Proportion of individuals having ever been tested for HIV among overall population (%)	2000	3·3 (2·8 to 3·9)	2·9 (2·4 to 3·4)	3·7 (3·1 to 4·4)	3·3 (2·8 to 3·9)	4·6 (3·9 to 5·4)	2·8 (2·4 to 3·4)	2·1 (1·7 to 2·5)
	2005	13 (12 to 14)	11 (9·9 to 12)	14 (13 to 15)	11 (10 to 12)	17 (16 to 19)	12 (11 to 13)	8·6 (7·9 to 9·4)
	2010	41 (39 to 42)	37 (35 to 39)	44 (43 to 46)	37 (35 to 39)	51 (49 to 53)	41 (40 to 43)	32 (30 to 34)
	2015	53 (52 to 54)	49 (47 to 50)	58 (57 to 59)	41 (40 to 42)	66 (65 to 67)	61 (60 to 62)	49 (48 to 51)
	2020	64 (62 to 66)	59 (57 to 61)	68 (66 to 71)	48 (45 to 52)	74 (73 to 76)	74 (73 to 76)	65 (63 to 67)
Proportion of PLHIV who know their HIV status (%)	2000	4·8 (4·0 to 5·8)	4·0 (3·3 to 4·8)	5·4 (4·4 to 6·5)	4·1 (3·3 to 4·9)	6·1 (5·1 to 7·4)	4·1 (3·4 to 4·9)	3·0 (2·4 to 3·6)
	2005	19 (17 to 21)	16 (14 to 17)	21 (19 to 23)	14 (12 to 16)	23 (20 to 25)	18 (16 to 20)	14 (12 to 15)
	2010	56 (53 to 58)	49 (47 to 51)	60 (57 to 62)	44 (41 to 47)	60 (57 to 63)	57 (55 to 60)	53 (50 to 55)
	2015	74 (72 to 75)	67 (65 to 69)	78 (77 to 80)	57 (55 to 59)	74 (72 to 76)	78 (76 to 79)	77 (75 to 79)
	2020	86 (85 to 88)	81 (79 to 83)	90 (89 to 91)	71 (69 to 74)	84 (83 to 86)	89 (88 to 90)	92 (91 to 93)
Time to diagnosis or AIDS-related death, median (year)	2000	11 (10 to 11)	11 (10 to 11)	11 (10 to 11)	12 (12 to 13)	10 (9·8 to 11)	9·0 (8·8 to 9·2)	8·0 (7·8 to 8·0)
	2005	7·3 (6·3 to 8·2)	7·8 (6·9 to 8·5)	6·9 (5·8 to 7·9)	7·6 (6·4 to 8·8)	6·9 (5·9 to 7·8)	7·3 (6·7 to 7·8)	6·8 (6·4 to 7·1)
	2010	3·2 (2·8 to 3·6)	4·0 (3·6 to 4·4)	2·6 (2·3 to 3·0)	3·1 (2·7 to 3·5)	2·8 (2·4 to 3·2)	4·0 (3·6 to 4·4)	4·2 (3·9 to 4·6)
	2015	2·6 (2·4 to 2·9)	3·4 (3·1 to 3·7)	2·0 (1·8 to 2·3)	2·6 (2·3 to 2·9)	2·2 (2·0 to 2·4)	3·2 (2·9 to 3·4)	3·4 (3·2 to 3·7)
	2020	2·0 (1·5 to 2·7)	2·7 (2·0 to 3·5)	1·5 (1·1 to 2·1)	2·0 (1·4 to 2·7)	1·7 (1·3 to 2·3)	2·4 (1·8 to 3·1)	2·6 (2·0 to 3·3)
Probability of getting tested within 1 year following infection (%)	2000	1·7 (1·4 to 2·1)	1·5 (1·2 to 1·8)	1·9 (1·6 to 2·3)	1·9 (1·5 to 2·3)	2·1 (1·7 to 2·5)	1·2 (1·0 to 1·4)	0·9 (0·8 to 1·1)
	2005	5·8 (4·4 to 7·6)	4·7 (3·6 to 6·1)	6·5 (4·9 to 8·6)	6·2 (4·7 to 8·2)	6·8 (5·2 to 8·9)	3·8 (2·9 to 5·0)	3·1 (2·4 to 4·1)
	2010	25 (21 to 29)	18 (15 to 22)	30 (25 to 35)	27 (22 to 32)	29 (24 to 35)	16 (13 to 19)	12 (10 to 15)
	2015	31 (28 to 35)	22 (19 to 24)	38 (34 to 42)	33 (29 to 36)	37 (33 to 41)	21 (19 to 24)	17 (15 to 19)
	2020	40 (29 to 52)	28 (20 to 39)	48 (36 to 61)	41 (30 to 53)	47 (35 to 60)	29 (20 to 40)	24 (17 to 34)
Probability of getting tested before reaching a CD4 count lower than 350 cells per µL (%)	2000	14 (11 to 16)	11 (9·6 to 14)	15 (12 to 18)	17 (14 to 20)	14 (12 to 16)	7·6 (6·3 to 9·0)	5·1 (4·2 to 6·2)
	2005	35 (29 to 42)	30 (24 to 36)	38 (32 to 46)	43 (36 to 50)	36 (29 to 43)	21 (17 to 27)	15 (12 to 19)
	2010	66 (62 to 70)	58 (54 to 63)	71 (68 to 75)	73 (69 to 76)	71 (67 to 74)	50 (45 to 54)	38 (34 to 42)
	2015	71 (68 to 74)	63 (59 to 66)	77 (74 to 79)	76 (74 to 79)	76 (73 to 78)	58 (54 to 61)	45 (41 to 49)
	2020	77 (70 to 83)	69 (61 to 77)	82 (76 to 87)	81 (74 to 87)	81 (75 to 86)	66 (57 to 74)	55 (45 to 64)
Positivity (% of positive test among all tests)	2000	11 (9·6 to 12)	8·5 (7·6 to 9·3)	12 (11 to 14)	5·2 (4·6 to 5·8)	17 (15 to 18)	15 (13 to 16)	5·9 (5·2 to 6·5)
	2005	10 (9·1 to 12)	7·9 (7·2 to 8·9)	12 (10 to 14)	4·5 (3·9 to 5·3)	15 (13 to 18)	16 (15 to 18)	6·7 (6·1 to 7·4)
	2010	5·6 (4·2 to 7·5)	4·0 (3·2 to 5·2)	6·6 (4·8 to 9·1)	2·8 (2·1 to 3·7)	7·3 (5·2 to 11)	9·8 (7·6 to 13)	4·2 (3·4 to 5·3)
	2015	4·0 (3·5 to 4·6)	3·5 (3·1 to 4·1)	4·3 (3·7 to 5·0)	2·4 (2·1 to 2·7)	4·5 (3·8 to 5·4)	6·7 (5·8 to 7·9)	3·7 (2·9 to 4·6)
	2020	2·5 (1·9 to 3·3)	2·3 (1·8 to 2·9)	2·6 (2·0 to 3·6)	1·5 (1·2 to 1·9)	2·7 (2·1 to 3·5)	4·0 (2·9 to 5·6)	2·8 (1·9 to 4·2)
Diagnosis yield (% of new diagnoses among all tests)	2000	9·7 (8·8 to 11)	7·9 (7·1 to 8·5)	11 (10 to 12)	4·8 (4·3 to 5·2)	15 (14 to 16)	14 (12 to 15)	5·6 (5·0 to 6·1)
	2005	7·6 (7·0 to 8·1)	6·4 (5·9 to 6·9)	8·5 (7·7 to 9·1)	3·6 (3·2 to 3·8)	10 (9·5 to 11)	13 (12 to 14)	5·8 (5·3 to 6·4)
	2010	2·4 (2·1 to 2·9)	2·2 (1·9 to 2·7)	2·6 (2·2 to 3·0)	1·5 (1·2 to 1·7)	2·6 (2·1 to 3·0)	4·8 (4·0 to 5·7)	2·7 (2·3 to 3·3)
	2015	1·7 (1·6 to 1·8)	1·7 (1·5 to 1·9)	1·7 (1·5 to 1·8)	1·3 (1·2 to 1·4)	1·5 (1·4 to 1·7)	2·7 (2·4 to 3)	2·0 (1·7 to 2·3)
	2020	1·0 (0·8 to 1·3)	1·0 (0·8 to 1·2)	1·0 (0·8 to 1·3)	0·9 (0·7 to 1·1)	1·0 (0·8 to 1·2)	1·4 (1·1 to 1·8)	1·1 (0·8 to 1·5)
Proportion of new HIV diagnoses among all positive tests (%)	2000	91 (85 to 95)	93 (89 to 96)	90 (83 to 95)	92 (87 to 96)	89 (82 to 94)	93 (89 to 96)	95 (92 to 97)
	2005	74 (61 to 84)	81 (71 to 88)	70 (57 to 82)	79 (68 to 88)	67 (54 to 80)	79 (68 to 87)	87 (79 to 92)
	2010	44 (32 to 59)	55 (43 to 68)	39 (28 to 55)	53 (39 to 68)	35 (24 to 50)	49 (38 to 62)	65 (52 to 76)
	2015	42 (37 to 48)	49 (43 to 55)	39 (33 to 45)	55 (49 to 62)	34 (29 to 41)	40 (34 to 46)	53 (43 to 65)
	2020	41 (30 to 52)	44 (34 to 54)	39 (28 to 52)	59 (49 to 70)	35 (26 to 47)	34 (24 to 46)	38 (25 to 56)

PLHIV: people living with HIV; HTS: HIV testing services.
Numbers in parentheses correspond to 95% credible intervals.

***Table S8*. Progress in HIV testing related outcomes in Southern Africa stratified by sex and age group, 2000-2020.**

Outcome	Year	Overall	Men	Women	15-24 years-old	25-34 years-old	35-49 years-old	50+ years-old
Proportion of individuals having ever been tested for HIV among overall population (%)	2000	10 (8·3 to 12·9)	8·5 (6·7 to 11)	12 (9·8 to 15)	8·8 (6·9 to 11)	14 (11 to 17)	11 (8·5 to 13)	8·4 (6·8 to 11)
	2005	30 (29 to 32)	25 (23 to 27)	34 (33 to 36)	22 (21 to 24)	39 (37 to 41)	34 (32 to 36)	27 (26 to 29)
	2010	59 (58 to 61)	52 (50 to 54)	65 (64 to 67)	44 (42 to 46)	69 (67 to 70)	68 (66 to 70)	58 (56 to 61)
	2015	75 (74 to 75)	68 (67 to 69)	80 (79 to 81)	53 (52 to 54)	82 (81 to 83)	86 (85 to 87)	79 (77 to 81)
	2020	85 (83 to 88)	80 (77 to 84)	90 (87 to 92)	62 (57 to 70)	90 (88 to 93)	95 (93 to 96)	92 (90 to 93)
Proportion of PLHIV who know their HIV status (%)	2000	9·3 (7·5 to 12)	6·9 (5·4 to 8·7)	11 (8·7 to 14)	7·0 (5·5 to 8·9)	11 (9·1 to 14)	8·9 (7·2 to 11)	7·8 (6·4 to 9·5)
	2005	27 (25 to 30)	21 (19 to 24)	31 (28 to 34)	18 (16 to 20)	32 (30 to 35)	28 (26 to 31)	25 (23 to 27)
	2010	60 (58 to 63)	51 (48 to 54)	66 (63 to 68)	41 (38 to 44)	65 (63 to 68)	64 (61 to 67)	59 (56 to 62)
	2015	79 (77 to 80)	72 (70 to 74)	82 (81 to 84)	55 (52 to 57)	80 (78 to 81)	84 (82 to 85)	81 (79 to 83)
	2020	90 (88 to 92)	87 (84 to 90)	92 (90 to 94)	70 (65 to 76)	89 (87 to 92)	93 (92 to 95)	93 (91 to 95)
Time to diagnosis or AIDS-related death, median (year)	2000	7·7 (7·0 to 8·4)	8·2 (7·6 to 8·7)	7·4 (6·6 to 8·1)	8·5 (7·7 to 9·3)	7·1 (6·4 to 7·8)	6·8 (6·3 to 7·2)	6·4 (6·1 to 6·6)
	2005	5·9 (5·1 to 6·6)	6·5 (5·8 to 7·2)	5·4 (4·6 to 6·2)	6·4 (5·5 to 7·2)	5·3 (4·6 to 6·1)	5·6 (4·9 to 6·2)	5·4 (4·9 to 5·8)
	2010	2·8 (2·5 to 3·1)	3·5 (3·2 to 3·8)	2·4 (2·1 to 2·7)	3·1 (2·8 to 3·5)	2·4 (2·1 to 2·6)	2·7 (2·4 to 3·0)	3·1 (2·8 to 3·4)
	2015	2·2 (1·9 to 2·4)	2·7 (2·4 to 3·0)	1·8 (1·6 to 2·1)	2·5 (2·2 to 2·7)	1·8 (1·6 to 2·0)	2·0 (1·8 to 2·3)	2·4 (2·1 to 2·6)
	2020	1·5 (0·9 to 2·3)	1·8 (1·1 to 2·7)	1·3 (0·8 to 2·0)	1·7 (1·0 to 2·7)	1·2 (0·7 to 1·9)	1·4 (0·9 to 2·2)	1·7 (1·0 to 2·5)
Probability of getting tested within 1 year following infection (%)	2000	4·2 (3·3 to 5·3)	3·1 (2·4 to 3·9)	4·9 (3·9 to 6·3)	3·9 (3·0 to 5·0)	5·1 (4·0 to 6·5)	3·6 (2·8 to 4·6)	3·1 (2·5 to 3·9)
	2005	7·5 (5·8 to 9·7)	5·7 (4·4 to 7·4)	8·7 (6·6 to 11)	7·1 (5·5 to 9·2)	9·2 (7·1 to 12)	6·4 (4·9 to 8·3)	5·6 (4·3 to 7·3)
	2010	23 (20 to 26)	17 (15 to 19)	27 (23 to 31)	20 (18 to 23)	29 (25 to 33)	21 (18 to 25)	17 (14 to 20)
	2015	28 (25 to 32)	22 (19 to 25)	33 (29 to 37)	25 (22 to 29)	35 (31 to 39)	27 (23 to 31)	22 (19 to 25)
	2020	40 (26 to 60)	32 (20 to 50)	44 (29 to 65)	36 (23 to 56)	49 (33 to 69)	38 (24 to 57)	32 (20 to 49)
Probability of getting tested before reaching a CD4 count lower than 350 cells per µL (%)	2000	30 (25 to 35)	23 (19 to 28)	34 (29 to 40)	34 (29 to 40)	31 (25 to 37)	20 (17 to 25)	15 (12 to 18)
	2005	43 (37 to 50)	36 (30 to 42)	48 (41 to 55)	49 (43 to 55)	45 (39 to 52)	32 (26 to 39)	24 (20 to 30)
	2010	69 (66 to 72)	61 (58 to 65)	74 (71 to 77)	72 (69 to 74)	74 (71 to 77)	62 (58 to 66)	49 (45 to 53)
	2015	75 (72 to 77)	68 (65 to 71)	79 (76 to 81)	76 (74 to 79)	79 (77 to 82)	69 (65 to 72)	57 (53 to 61)
	2020	81 (72 to 89)	77 (65 to 86)	84 (75 to 91)	83 (74 to 90)	86 (78 to 92)	78 (66 to 86)	67 (53 to 78)
Positivity (% of positive test among all tests)	2000	15 (13 to 19)	12 (10 to 14)	18 (15 to 22)	14 (13 to 16)	24 (21 to 29)	14 (12 to 16)	3·3 (2·7 to 4·1)
	2005	20 (16 to 26)	15 (13 to 19)	23 (19 to 31)	14 (12 to 17)	31 (26 to 41)	21 (18 to 27)	5·3 (4·5 to 6·8)
	2010	13 (9·0 to 22)	10 (7·7 to 15)	15 (9·9 to 25)	9·5 (7·4 to 14)	19 (12 to 32)	16 (11 to 25)	4·1 (3·0 to 5·9)
	2015	9·2 (7·1 to 13)	7·6 (6·2 to 9·8)	10 (7·7 to 14)	6·5 (5·5 to 8·0)	12 (8·9 to 17)	13 (9·9 to 17)	3·5 (2·8 to 5·0)
	2020	5·5 (3·8 to 8·4)	5·2 (3·6 to 7·5)	5·8 (3·9 to 9·0)	3·5 (2·7 to 4·6)	6·3 (4·2 to 9·8)	8·4 (5·5 to 13)	3·0 (2·0 to 5·4)
Diagnosis yield (% of new diagnoses among all tests)	2000	13 (12 to 14)	11 (9·4 to 11)	15 (14 to 16)	13 (12 to 14)	20 (18 to 22)	12 (11 to 13)	2·8 (2·5 to 3·1)
	2005	14 (12 to 15)	11 (11 to 12)	15 (13 to 16)	11 (10 to 12)	20 (16 to 22)	15 (13 to 16)	4·1 (3·8 to 4·4)
	2010	6·9 (6·2 to 7·5)	6·5 (5·9 to 7·1)	7·2 (6·4 to 7·9)	6·6 (6·1 to 7·2)	8·3 (7·1 to 9·3)	8·5 (7·6 to 9·4)	2·7 (2·4 to 3·0)
	2015	4·4 (4·1 to 4·6)	4·3 (3·9 to 4·6)	4·4 (4·1 to 4·7)	4·5 (4·2 to 4·8)	4·8 (4·4 to 5·2)	5·5 (5·1 to 5·9)	2·0 (1·9 to 2·2)
	2020	2·2 (1·6 to 2·9)	2·2 (1·5 to 3·0)	2·2 (1·6 to 2·8)	2·3 (1·7 to 2·9)	2·2 (1·5 to 2·9)	2·8 (1·9 to 3·8)	1·2 (0·9 to 1·8)
Proportion of new HIV diagnoses among all positive tests (%)	2000	87 (70 to 97)	91 (77 to 98)	86 (67 to 97)	91 (78 to 98)	85 (65 to 97)	88 (72 to 98)	87 (70 to 98)
	2005	70 (48 to 87)	78 (58 to 91)	67 (44 to 86)	81 (61 to 93)	64 (40 to 84)	72 (50 to 88)	78 (58 to 90)
	2010	53 (31 to 76)	64 (41 to 82)	49 (27 to 73)	70 (46 to 88)	44 (23 to 70)	54 (33 to 75)	67 (46 to 84)
	2015	47 (34 to 60)	56 (43 to 68)	43 (31 to 57)	70 (57 to 80)	40 (28 to 54)	43 (31 to 56)	58 (41 to 73)
	2020	39 (25 to 55)	42 (28 to 56)	37 (23 to 55)	66 (50 to 79)	35 (22 to 51)	34 (20 to 49)	41 (22 to 61)

PLHIV: people living with HIV; HTS: HIV testing services.

Numbers in parentheses correspond to 95% credible intervals.

***Text S2*. Comparisons of calibrated *Shiny90* model fits with programmatic and survey data for each of the 40 included sub-Saharan countries, 2000-2020.**

For each country, *Shiny90* posterior estimates of the total number of HIV tests (A), the total number of positive tests (B), the proportion reporting having ever been tested among the HIV negative population (C), among people living with HIV (PLHIV; D), and overall (E) are compared to available HTS program and survey data. On all graphs, the lines correspond to the median of the posterior estimates, whereas the shaded areas correspond to the 95% credible intervals. Black dots on graphs A and B correspond to the HIV testing services program data for the overall number of tests, and for the number of positive tests, respectively. The points on the graphs C to E correspond to survey estimates of the proportion of men (circles) and women (squares) reporting having ever been tested among the HIV negative population (C), PLHIV (D), and overall population (E). The lines correspond to the 95% credible intervals of the surveys estimates. For countries denoted by an asterisk (*), surveys for which datasets are not yet publicly available are not plotted. However, these estimates were included in model calibration and in the knowledge of status estimates reported to UNAIDS.

Table of figures

Angola 18
Benin 19
Botswana 20
Burkina Faso 21
Burundi 22
Cameroon* 23
Chad 24
Comoros 25
Congo 26
Cote d'Ivoire* 27
Democratic Republic of the Congo 28
Equatorial Guinea 29
Eritrea 30
eSwatini 31
Ethiopia 32
Gabon 33
Gambia 34
Ghana 35
Guinea 36
Kenya* 37
Lesotho* 38
Liberia 39
Madagascar 40
Malawi 41
Mali 42
Mauritania 43
Mozambique 44

Namibia* 45
Niger 46
Nigeria 47
Rwanda* 48
Senegal 49
Sierra Leone 50
South Africa 51
South Sudan 52
Tanzania 53
Togo 54
Uganda* 55
Zambia 56
Zimbabwe* 57

Angola

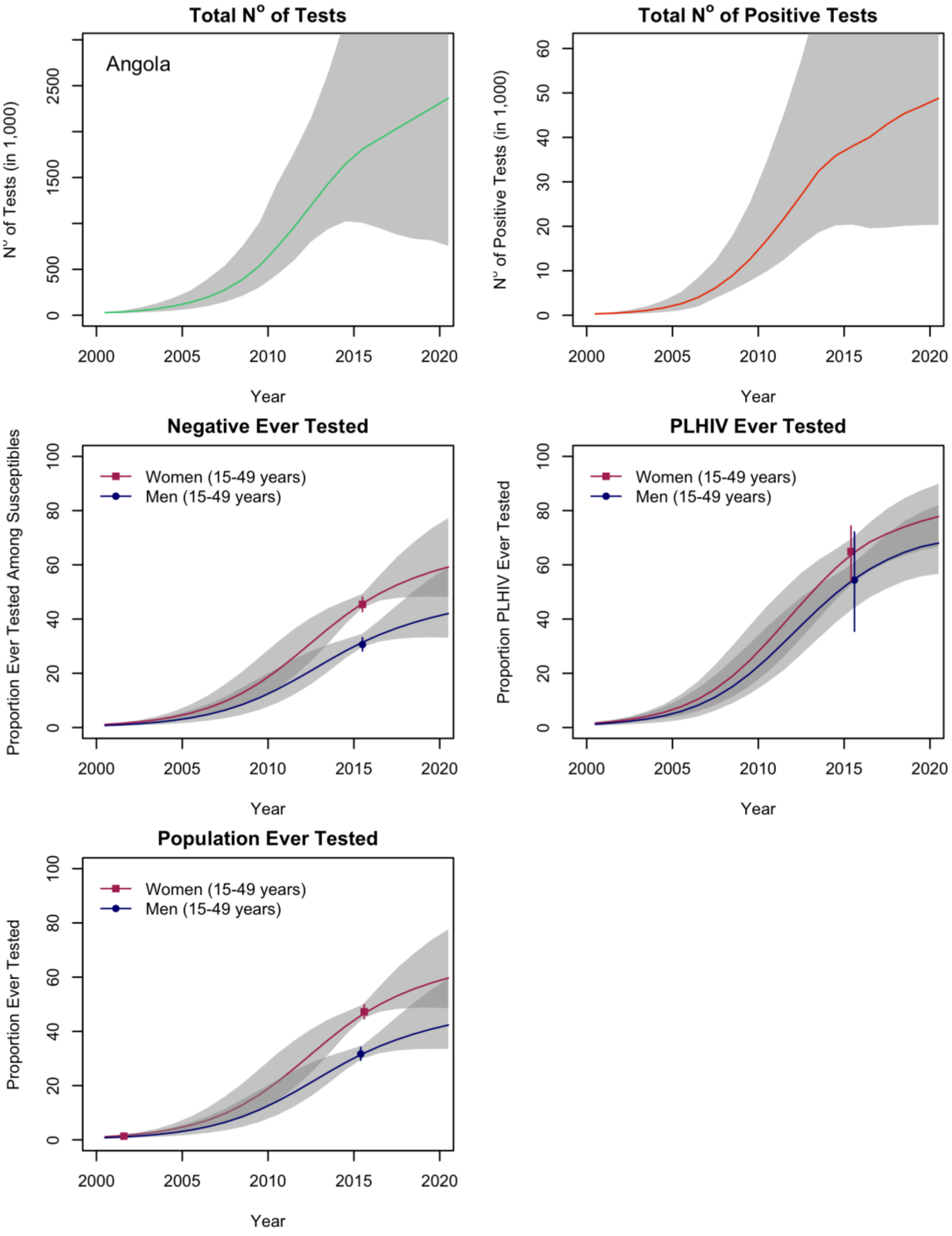
Total N° of Tests
Angola
N° of Tests (in 1,000)
0
500
1500
2500
2000
2005
2010
2015
2020
Year
Total N° of Positive Tests
N° of Positive Tests (in 1,000)
0
10
20
30
40
50
60
Negative Ever Tested
Proportion Ever Tested Among Susceptibles
Women (15-49 years)
Men (15-49 years)
PLHIV Ever Tested
Proportion PLHIV Ever Tested
Population Ever Tested
Proportion Ever Tested
0
20
40
60
80
100

Benin

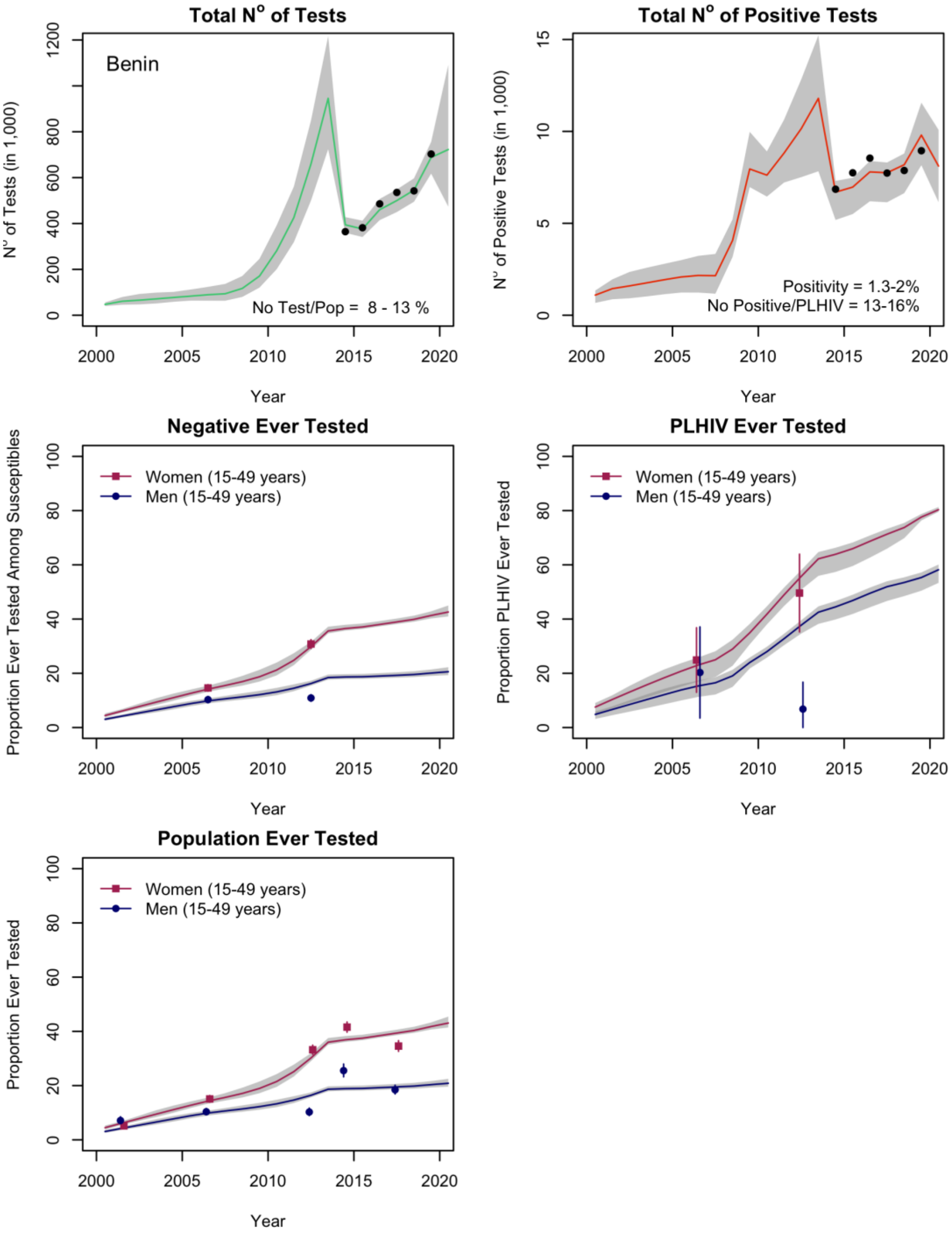
Total N° of Tests
Benin
N° of Tests (in 1,000)
No Test/Pop = 8 - 13 %
Year
Total N° of Positive Tests
N° of Positive Tests (in 1,000)
Positivity = 1.3-2%
No Positive/PLHIV = 13-16%
Year
Negative Ever Tested
Proportion Ever Tested Among Susceptibles
Women (15-49 years)
Men (15-49 years)
Year
PLHIV Ever Tested
Proportion PLHIV Ever Tested
Women (15-49 years)
Men (15-49 years)
Year
Population Ever Tested
Proportion Ever Tested
Women (15-49 years)
Men (15-49 years)
Year

Botswana

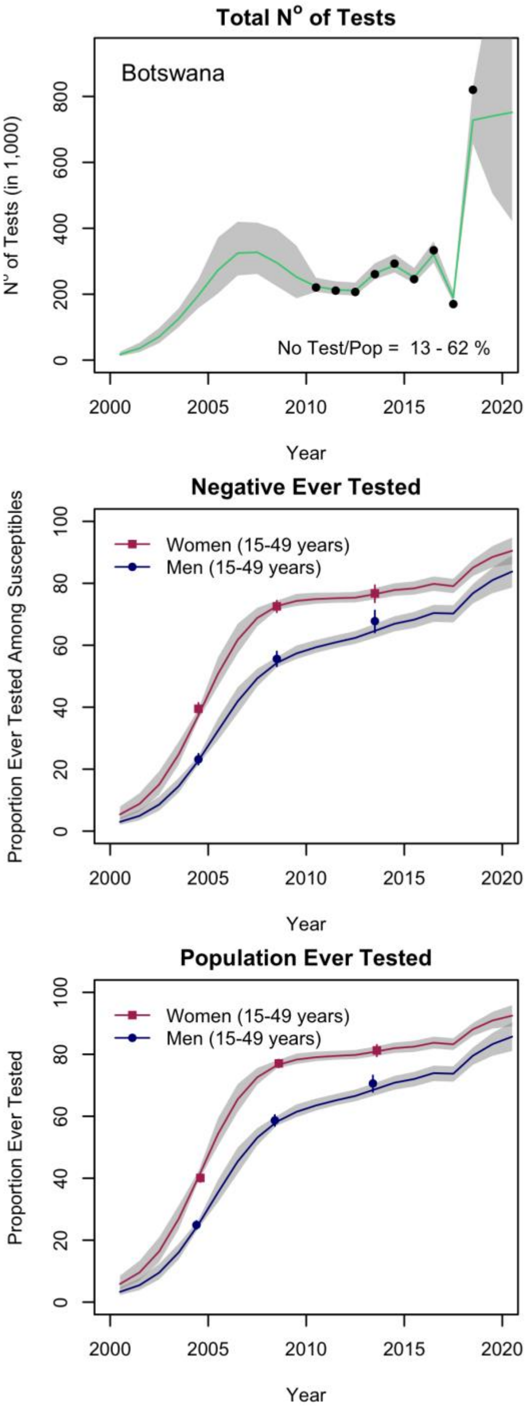

Total N° of Tests
Botswana
N° of Tests (in 1,000)
No Test/Pop = 13 - 62 %
Year
Total N° of Positive Tests
N° of Positive Tests (in 1,000)
Positivity = 5-18.9%
No Positive/PLHIV = 7-16%
Year
Negative Ever Tested
Proportion Ever Tested Among Susceptibles
Women (15-49 years)
Men (15-49 years)
Year
PLHIV Ever Tested
Proportion PLHIV Ever Tested
Women (15-49 years)
Men (15-49 years)
Year
Population Ever Tested
Proportion Ever Tested
Women (15-49 years)
Men (15-49 years)
Year

Burkina Faso

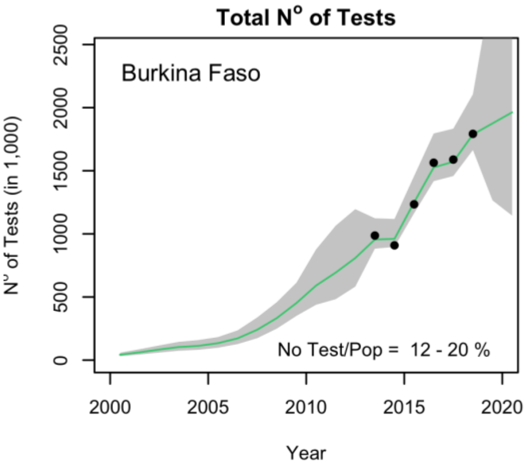

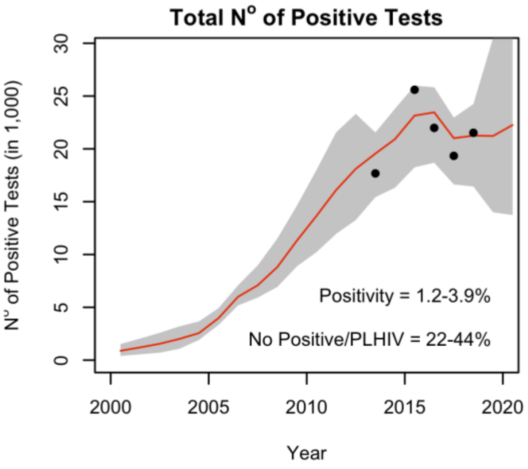

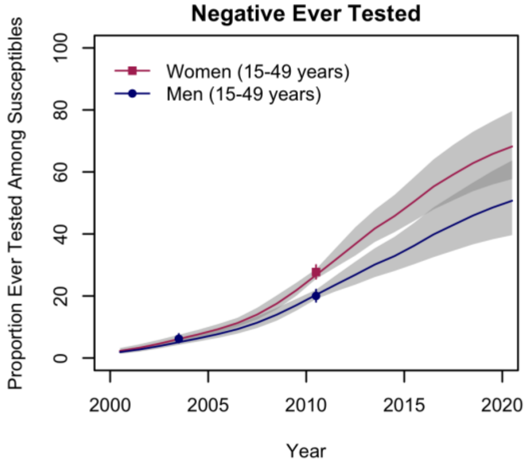

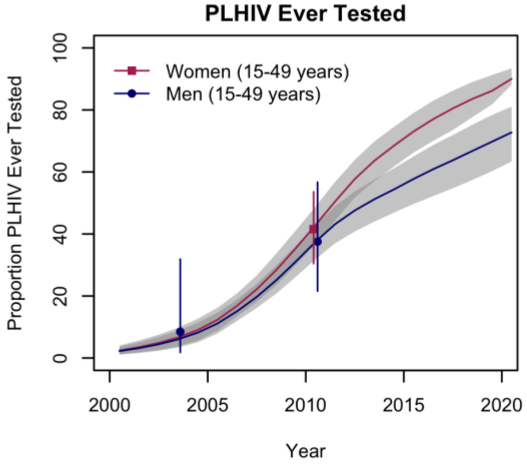

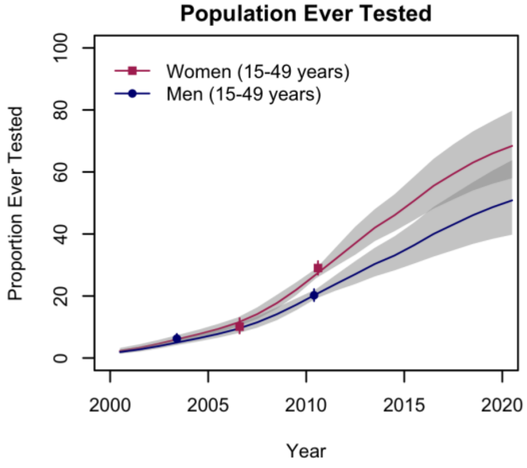

Burundi

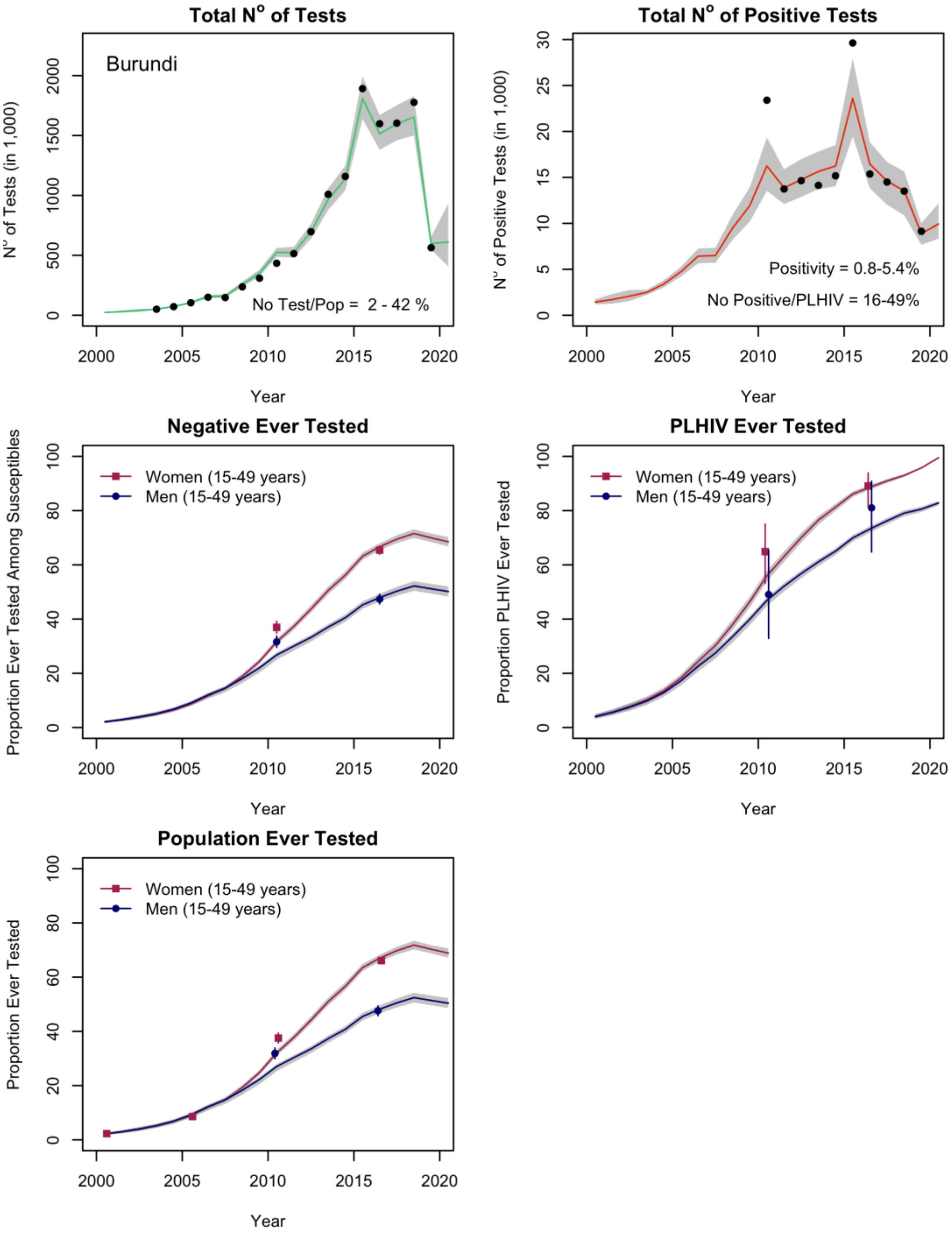
Total N° of Tests
Burundi
N° of Tests (in 1,000)
No Test/Pop = 2 - 42 %
Year
Total N° of Positive Tests
N° of Positive Tests (in 1,000)
Positivity = 0.8-5.4%
No Positive/PLHIV = 16-49%
Year
Negative Ever Tested
Proportion Ever Tested Among Susceptibles
Women (15-49 years)
Men (15-49 years)
Year
PLHIV Ever Tested
Proportion PLHIV Ever Tested
Women (15-49 years)
Men (15-49 years)
Year
Population Ever Tested
Proportion Ever Tested
Women (15-49 years)
Men (15-49 years)
Year

Cameroon*

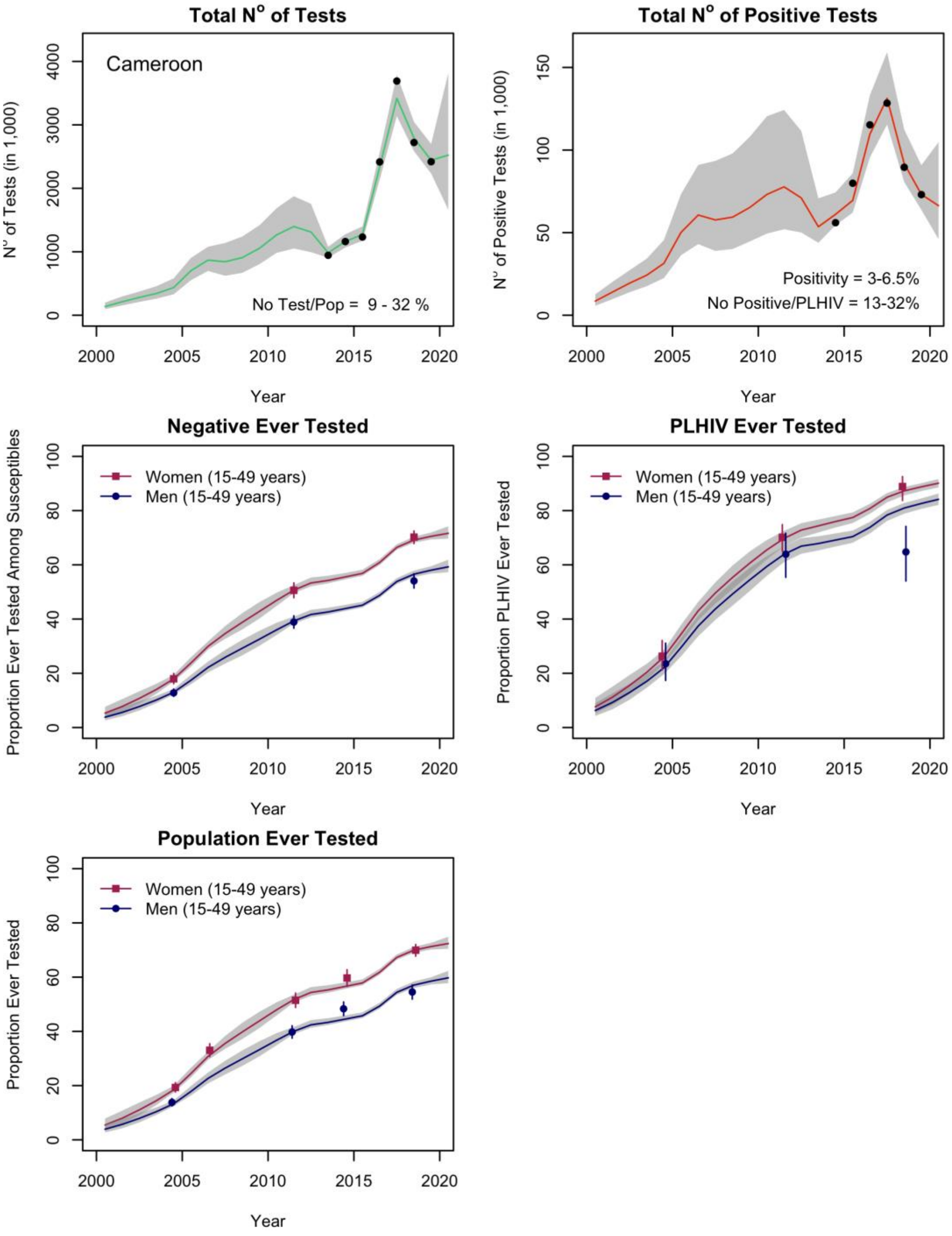

Total N° of Tests
Cameroon
N° of Tests (in 1,000)
No Test/Pop = 9 - 32 %
Year
Total N° of Positive Tests
N° of Positive Tests (in 1,000)
Positivity = 3-6.5%
No Positive/PLHIV = 13-32%
Year
Negative Ever Tested
Proportion Ever Tested Among Susceptibles
Women (15-49 years)
Men (15-49 years)
Year
PLHIV Ever Tested
Proportion PLHIV Ever Tested
Women (15-49 years)
Men (15-49 years)
Year
Population Ever Tested
Proportion Ever Tested
Women (15-49 years)
Men (15-49 years)
Year

Chad

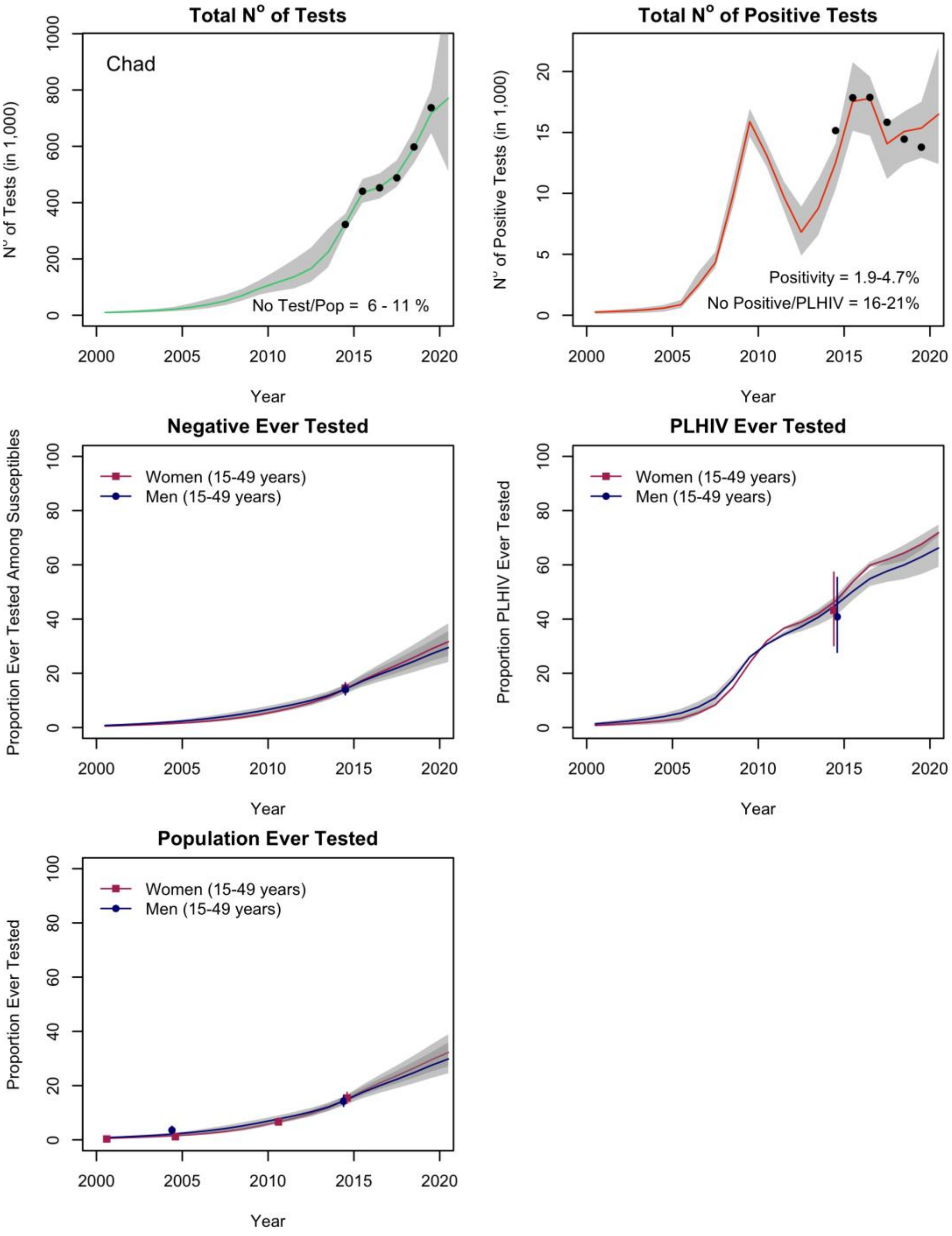

Total N° of Tests
Chad
N° of Tests (in 1,000)
No Test/Pop = 6 - 11 %
Year
Total N° of Positive Tests
N° of Positive Tests (in 1,000)
Positivity = 1.9-4.7%
No Positive/PLHIV = 16-21%
Year
Negative Ever Tested
Proportion Ever Tested Among Susceptibles
Women (15-49 years)
Men (15-49 years)
Year
PLHIV Ever Tested
Proportion PLHIV Ever Tested
Women (15-49 years)
Men (15-49 years)
Year
Population Ever Tested
Proportion Ever Tested
Women (15-49 years)
Men (15-49 years)
Year

Comoros

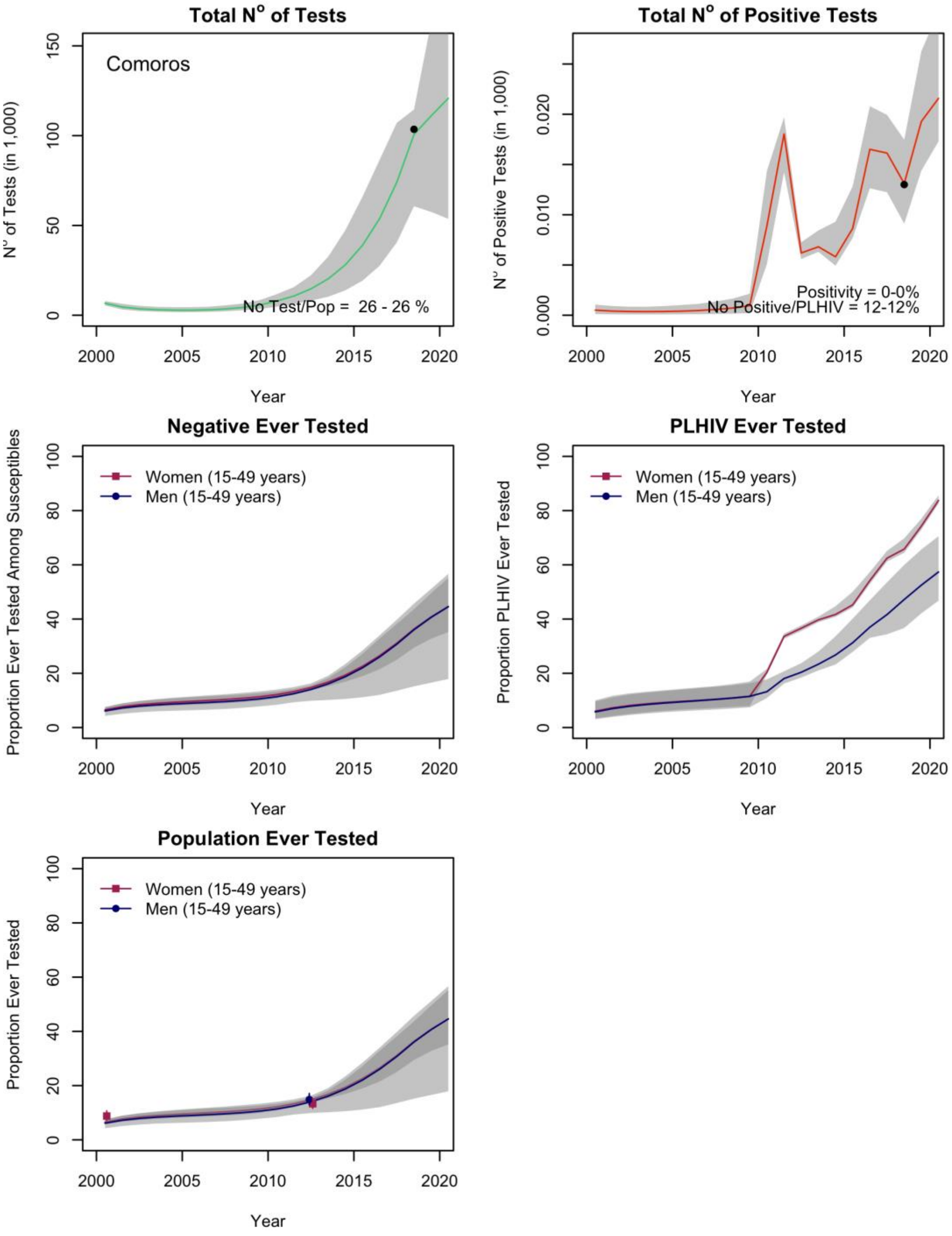

Total N° of Tests
Comoros
N° of Tests (in 1,000)
No Test/Pop = 26 - 26 %
Year
Total N° of Positive Tests
N° of Positive Tests (in 1,000)
Positivity = 0-0%
No Positive/PLHIV = 12-12%
Year
Negative Ever Tested
Proportion Ever Tested Among Susceptibles
Women (15-49 years)
Men (15-49 years)
Year
PLHIV Ever Tested
Proportion PLHIV Ever Tested
Women (15-49 years)
Men (15-49 years)
Year
Population Ever Tested
Proportion Ever Tested
Women (15-49 years)
Men (15-49 years)
Year

Congo

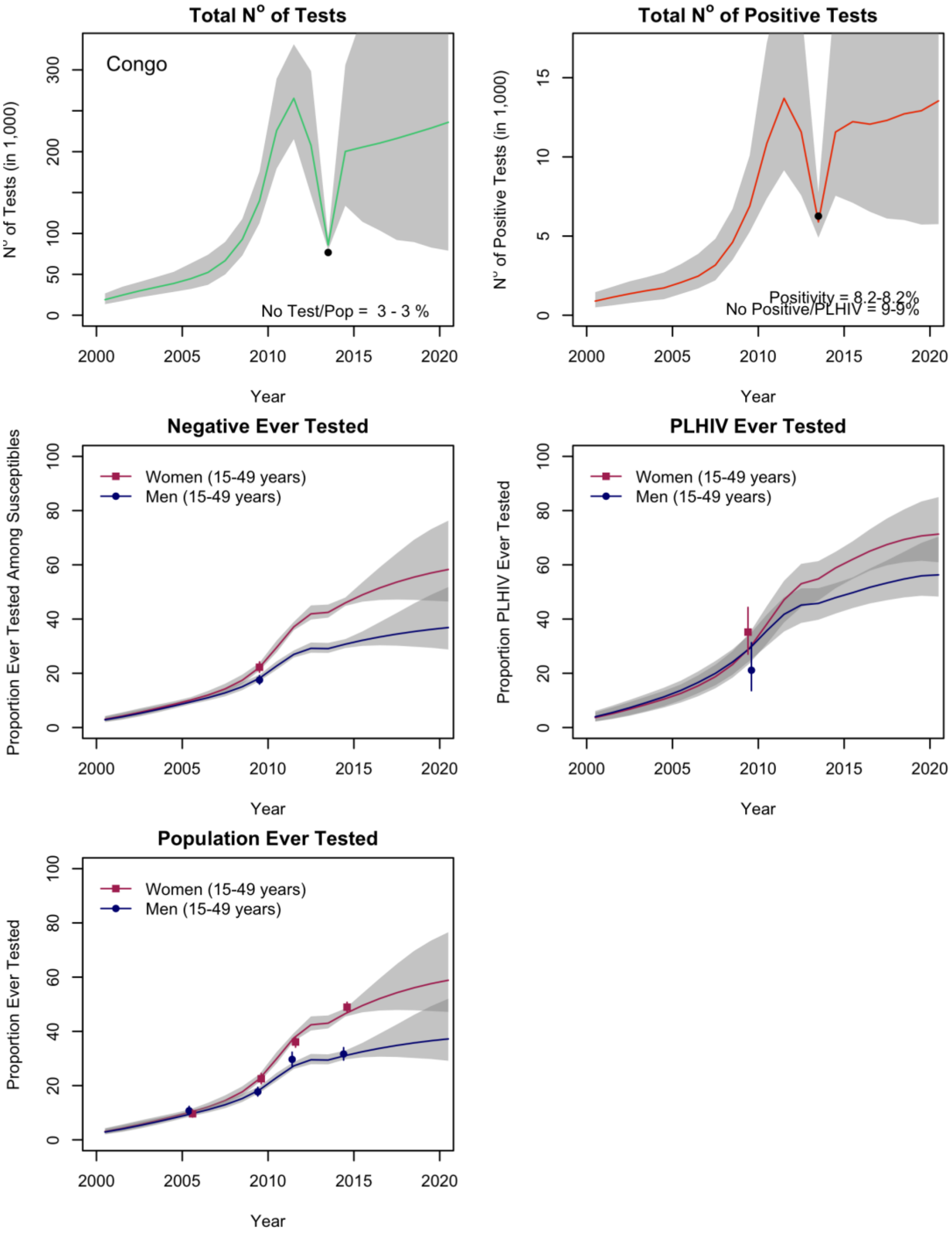

Total N° of Tests
Congo
N° of Tests (in 1,000)
No Test/Pop = 3 - 3 %
Year
Total N° of Positive Tests
N° of Positive Tests (in 1,000)
Positivity = 8.2-8.2%
No Positive/PLHIV = 9-9%
Year
Negative Ever Tested
Proportion Ever Tested Among Susceptibles
Women (15-49 years)
Men (15-49 years)
Year
PLHIV Ever Tested
Proportion PLHIV Ever Tested
Women (15-49 years)
Men (15-49 years)
Year
Population Ever Tested
Proportion Ever Tested
Women (15-49 years)
Men (15-49 years)
Year

Cote d'Ivoire*

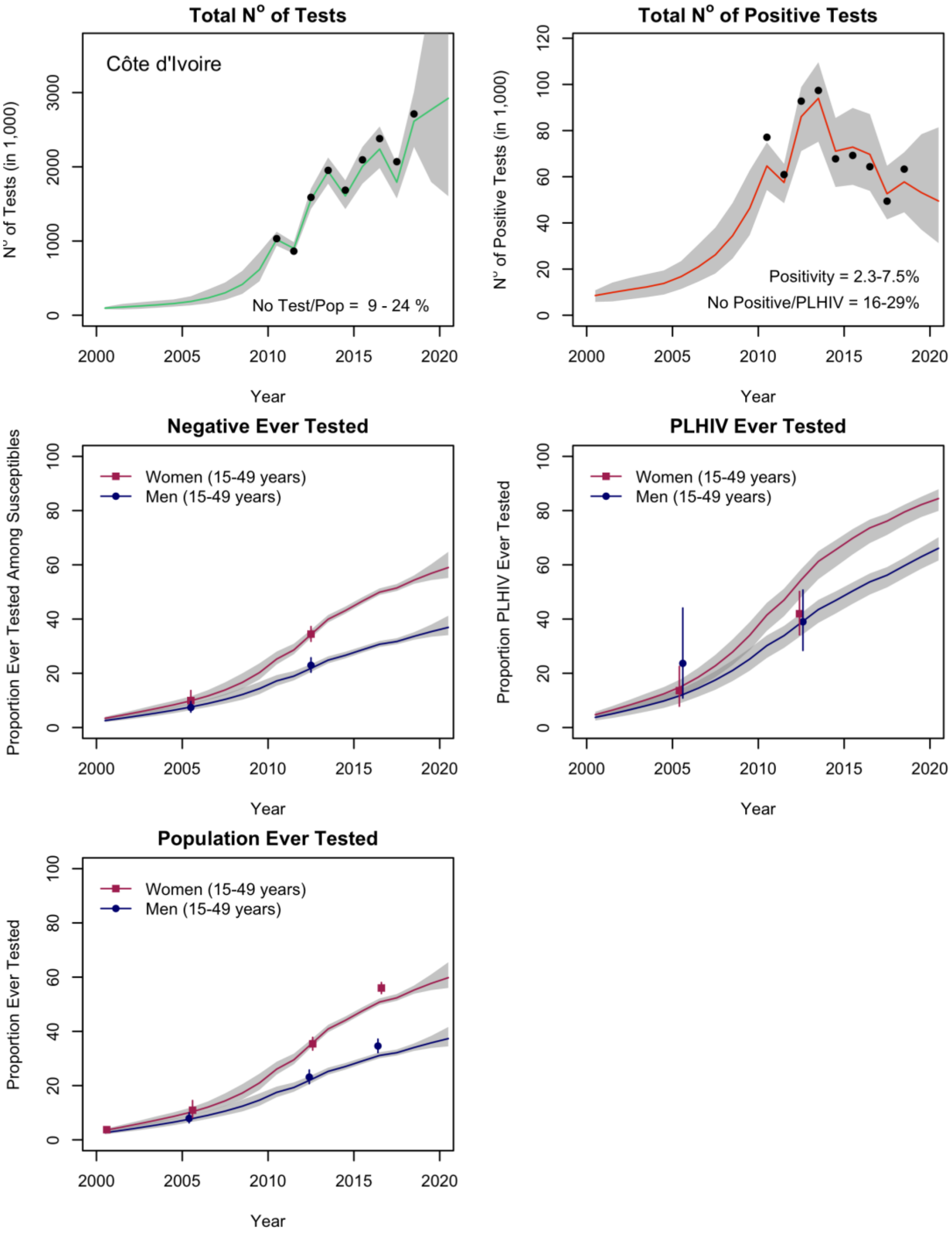

Democratic Republic of the Congo

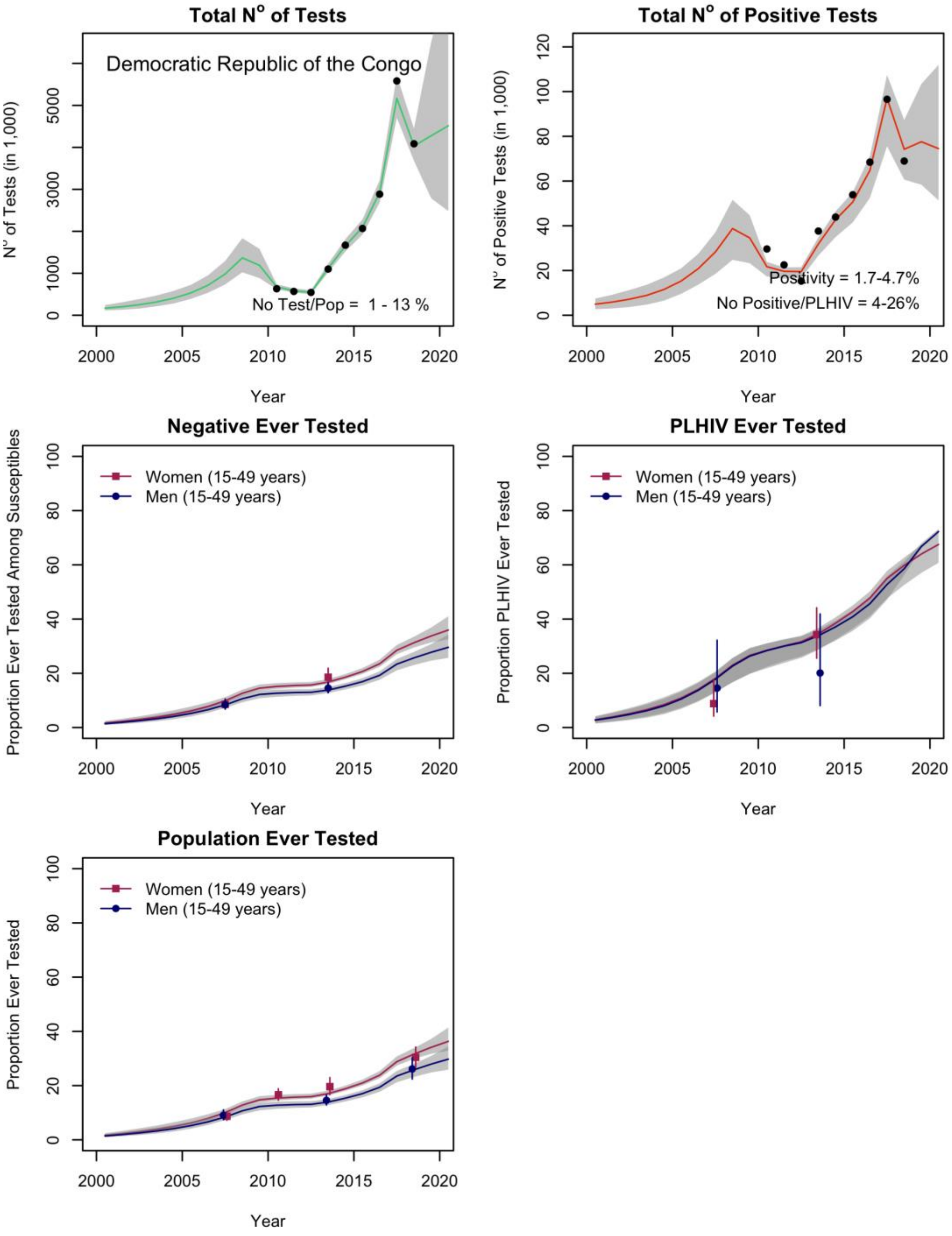

Equatorial Guinea

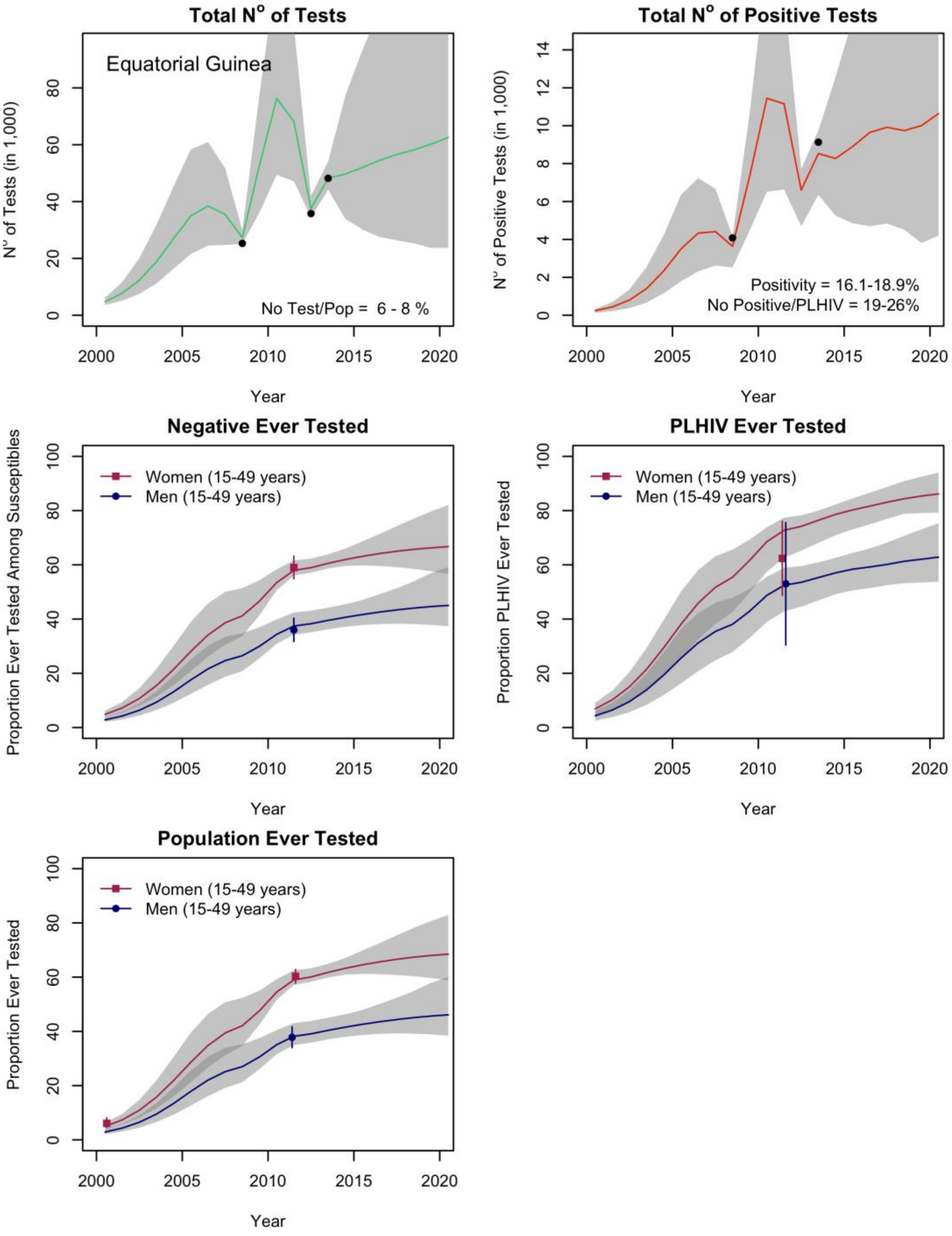

Eritrea

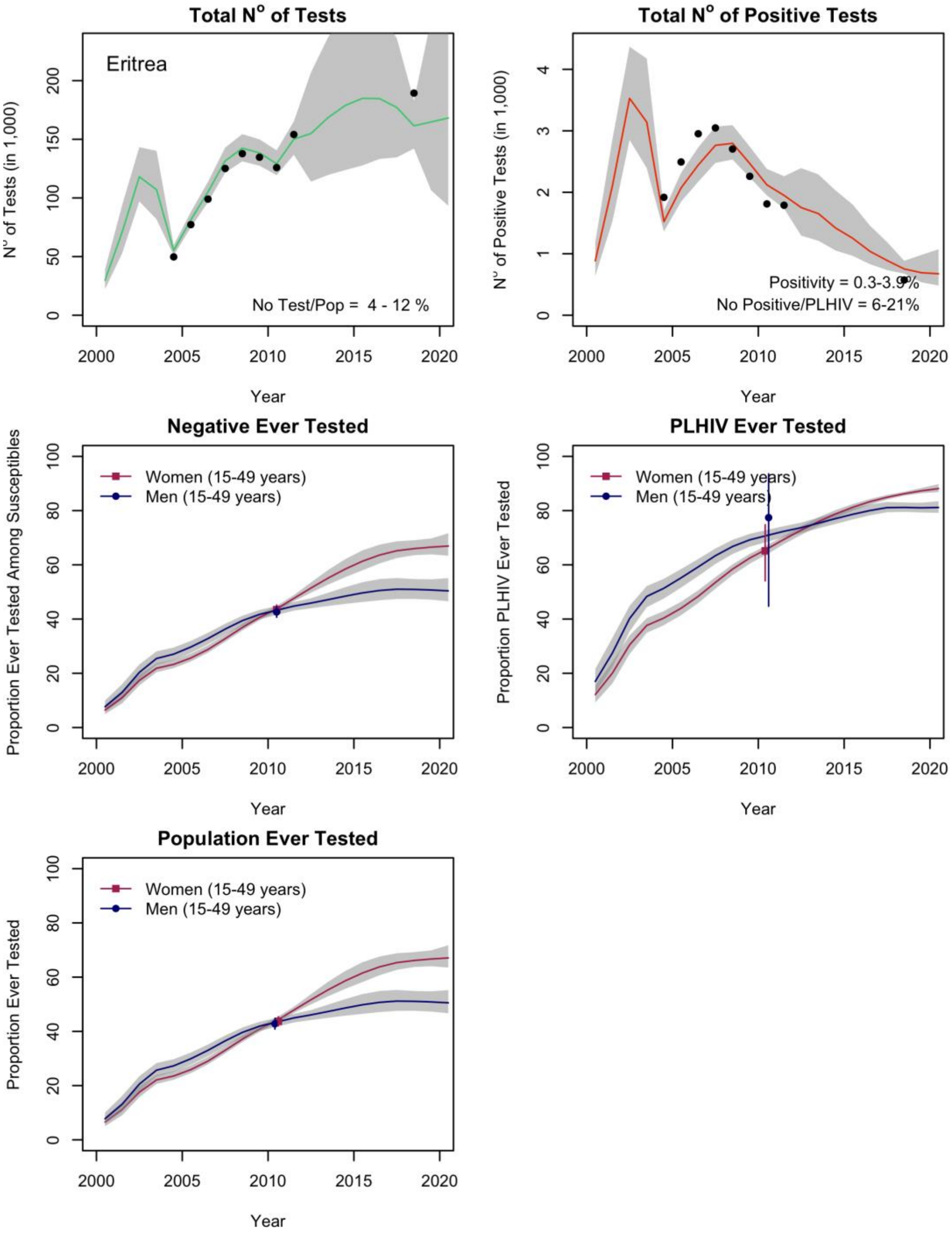
Total N° of Tests
Eritrea
N° of Tests (in 1,000)
No Test/Pop = 4 - 12 %
Year
Total N° of Positive Tests
N° of Positive Tests (in 1,000)
Positivity = 0.3-3.9%
No Positive/PLHIV = 6-21%
Year
Negative Ever Tested
Proportion Ever Tested Among Susceptibles
Women (15-49 years)
Men (15-49 years)
Year
PLHIV Ever Tested
Proportion PLHIV Ever Tested
Women (15-49 years)
Men (15-49 years)
Year
Population Ever Tested
Proportion Ever Tested
Women (15-49 years)
Men (15-49 years)
Year

eSwatini

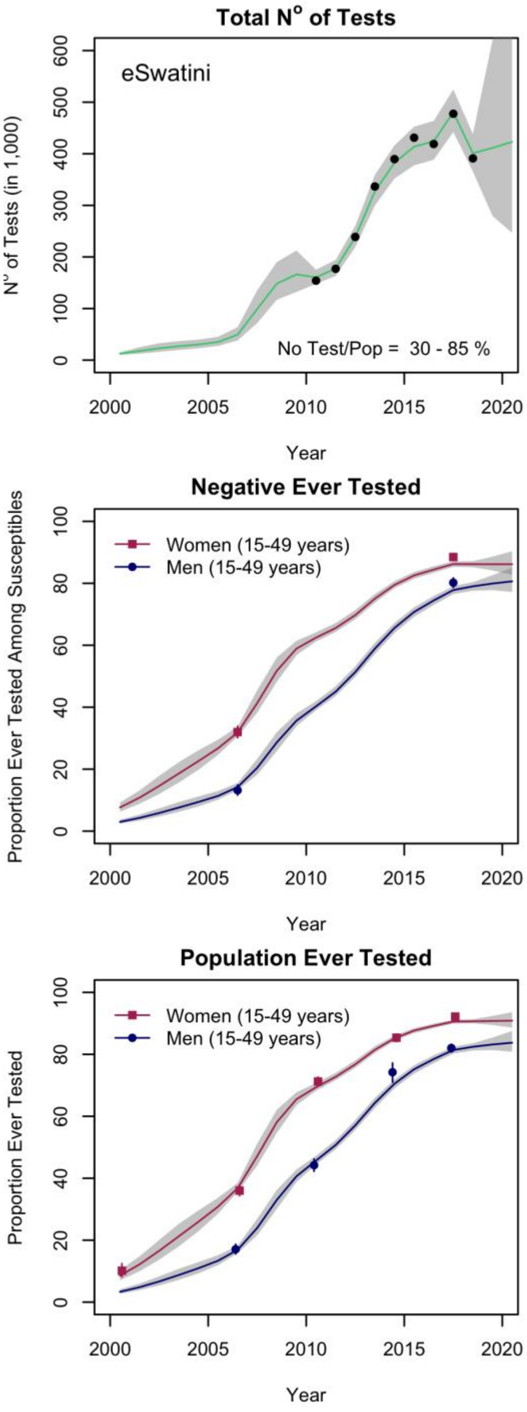
Total N° of Tests
eSwatini
No Test/Pop = 30 - 85 %
N° of Tests (in 1,000)
Year
Total N° of Positive Tests
Positivity = 5.5-23%
No Positive/PLHIV = 16-25%
N° of Positive Tests (in 1,000)
Year
Negative Ever Tested
Women (15-49 years)
Men (15-49 years)
Proportion Ever Tested Among Susceptibles
Year
PLHIV Ever Tested
Women (15-49 years)
Men (15-49 years)
Proportion PLHIV Ever Tested
Year
Population Ever Tested
Women (15-49 years)
Men (15-49 years)
Proportion Ever Tested
Year

Ethiopia

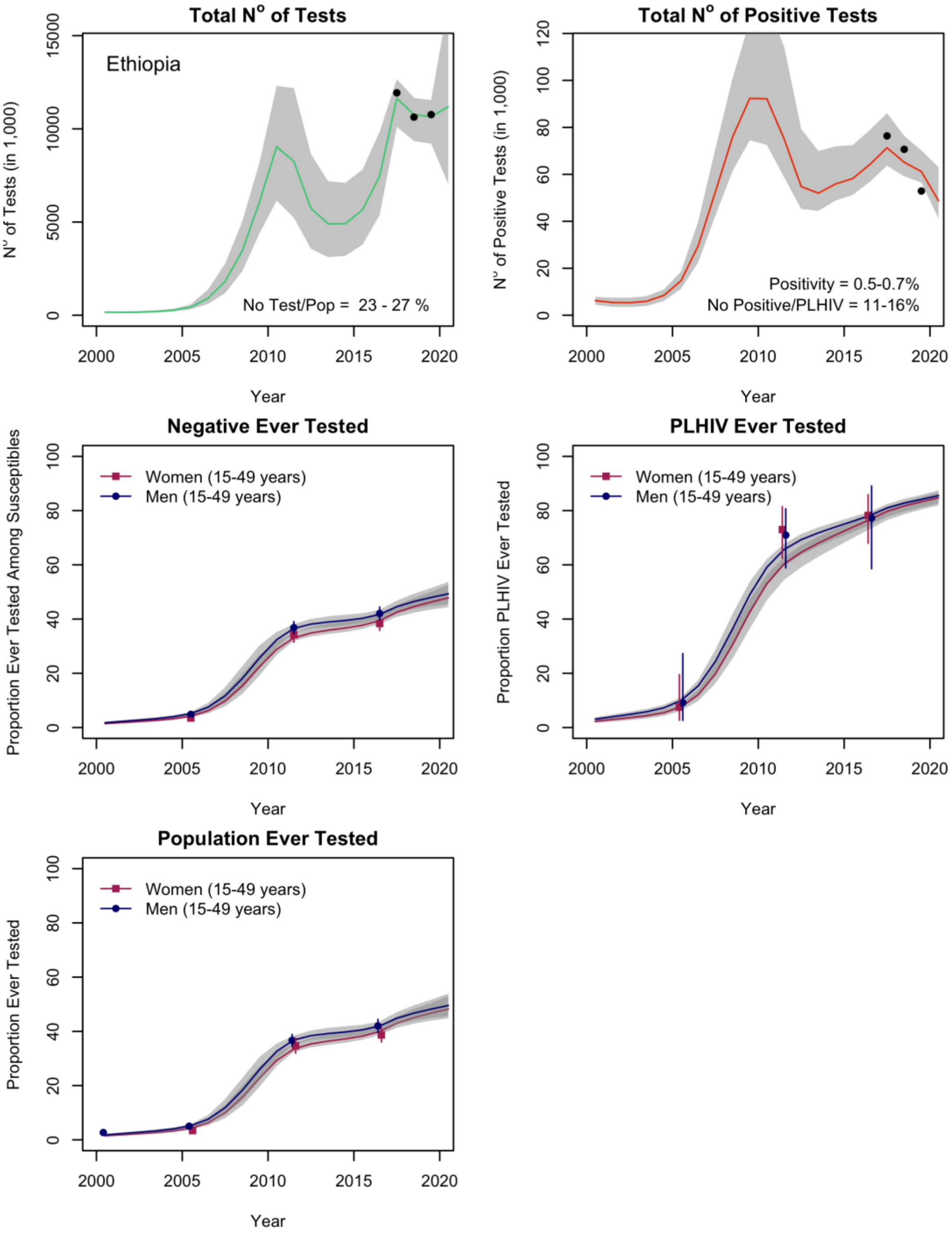

Total N° of Tests
Ethiopia
N° of Tests (in 1,000)
No Test/Pop = 23 - 27 %
Total N° of Positive Tests
N° of Positive Tests (in 1,000)
Positivity = 0.5-0.7%
No Positive/PLHIV = 11-16%
Negative Ever Tested
Proportion Ever Tested Among Susceptibles
PLHIV Ever Tested
Proportion PLHIV Ever Tested
Population Ever Tested
Proportion Ever Tested
Women (15-49 years)
Men (15-49 years)
Year

Gabon

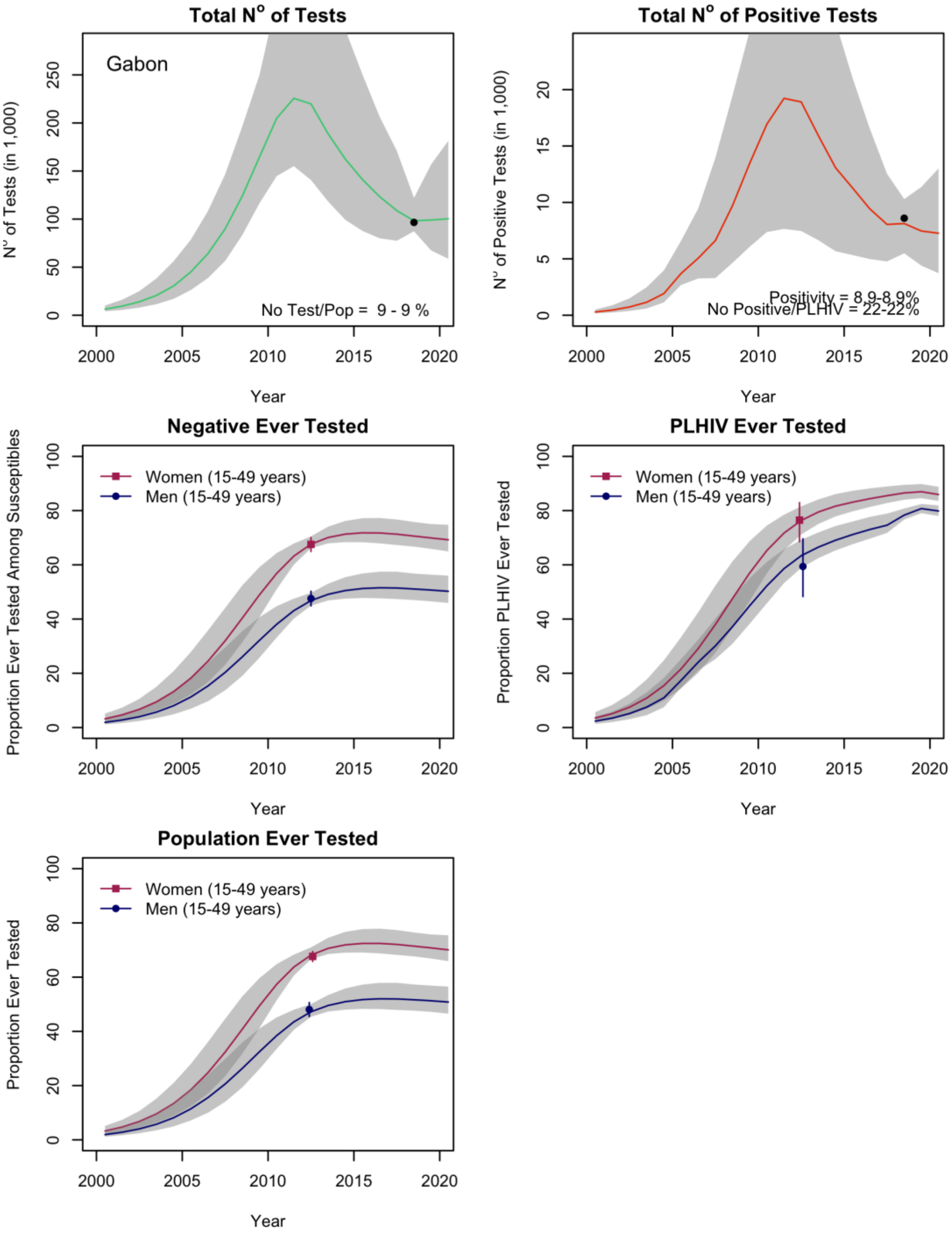
Total N° of Tests
Gabon
N° of Tests (in 1,000)
No Test/Pop = 9 - 9 %
Year
Total N° of Positive Tests
N° of Positive Tests (in 1,000)
Positivity = 8.9-8.9%
No Positive/PLHIV = 22-22%
Year
Negative Ever Tested
Proportion Ever Tested Among Susceptibles
Women (15-49 years)
Men (15-49 years)
Year
PLHIV Ever Tested
Proportion PLHIV Ever Tested
Women (15-49 years)
Men (15-49 years)
Year
Population Ever Tested
Proportion Ever Tested
Women (15-49 years)
Men (15-49 years)
Year

Gambia

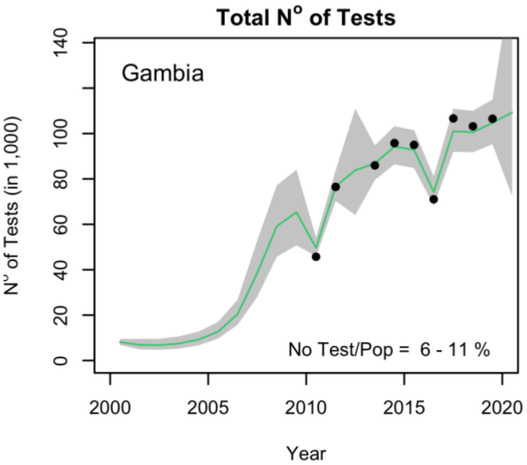
Total N° of Tests
Gambia
N° of Tests (in 1,000)
No Test/Pop = 6 - 11 %
Year
0
20
40
60
80
100
140
2000
2005
2010
2015
2020

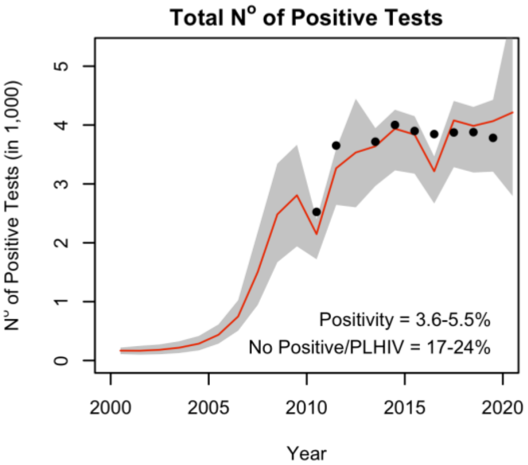
Total N° of Positive Tests
N° of Positive Tests (in 1,000)
Positivity = 3.6-5.5%
No Positive/PLHIV = 17-24%
Year
0
1
2
3
4
5
2000
2005
2010
2015
2020

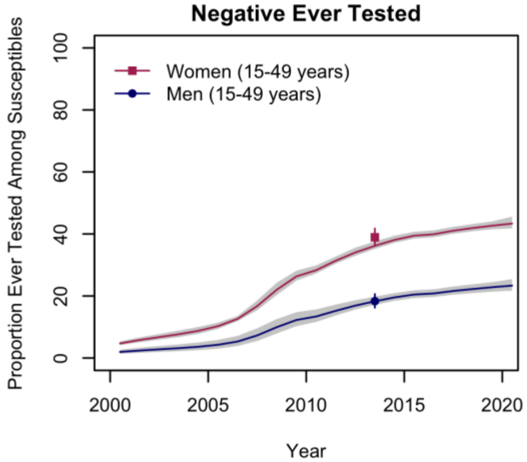
Negative Ever Tested
Proportion Ever Tested Among Susceptibles
Women (15-49 years)
Men (15-49 years)
Year
0
20
40
60
80
100
2000
2005
2010
2015
2020

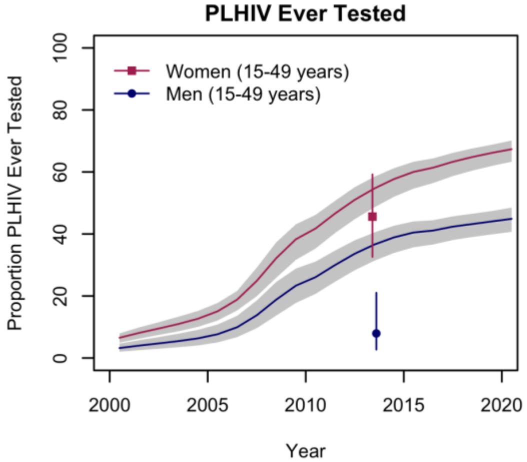
PLHIV Ever Tested
Proportion PLHIV Ever Tested
Women (15-49 years)
Men (15-49 years)
Year
0
20
40
60
80
100
2000
2005
2010
2015
2020

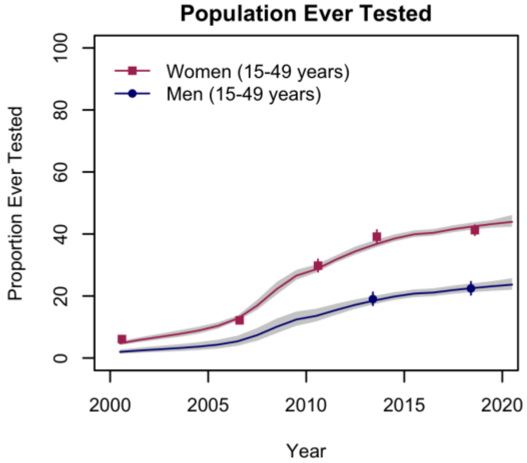
Population Ever Tested
Proportion Ever Tested
Women (15-49 years)
Men (15-49 years)
Year
0
20
40
60
80
100
2000
2005
2010
2015
2020

Ghana

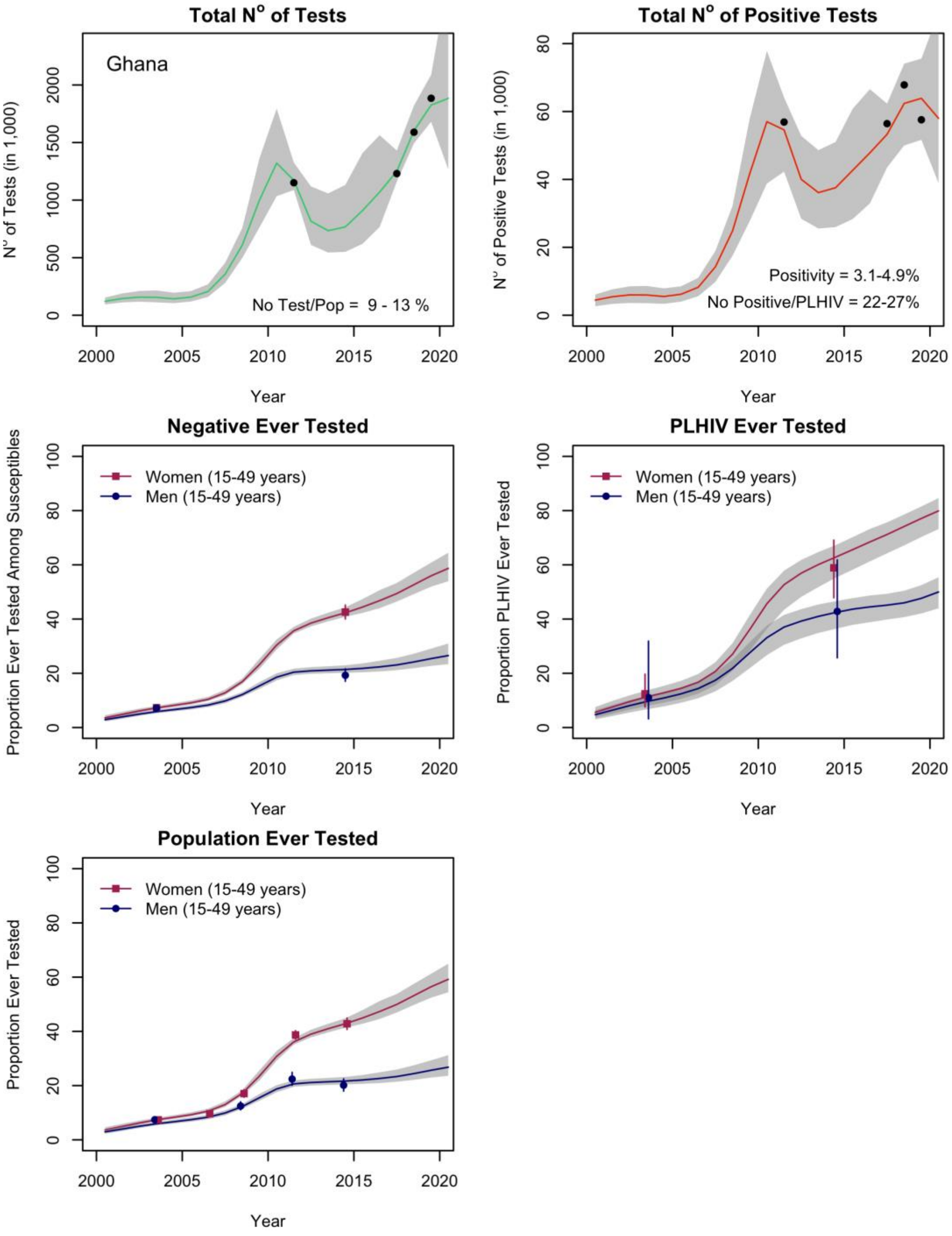
Total N° of Tests
Ghana
N° of Tests (in 1,000)
No Test/Pop = 9 - 13 %
Year
Total N° of Positive Tests
N° of Positive Tests (in 1,000)
Positivity = 3.1-4.9%
No Positive/PLHIV = 22-27%
Year
Negative Ever Tested
Proportion Ever Tested Among Susceptibles
Women (15-49 years)
Men (15-49 years)
Year
PLHIV Ever Tested
Proportion PLHIV Ever Tested
Women (15-49 years)
Men (15-49 years)
Year
Population Ever Tested
Proportion Ever Tested
Women (15-49 years)
Men (15-49 years)
Year

Guinea

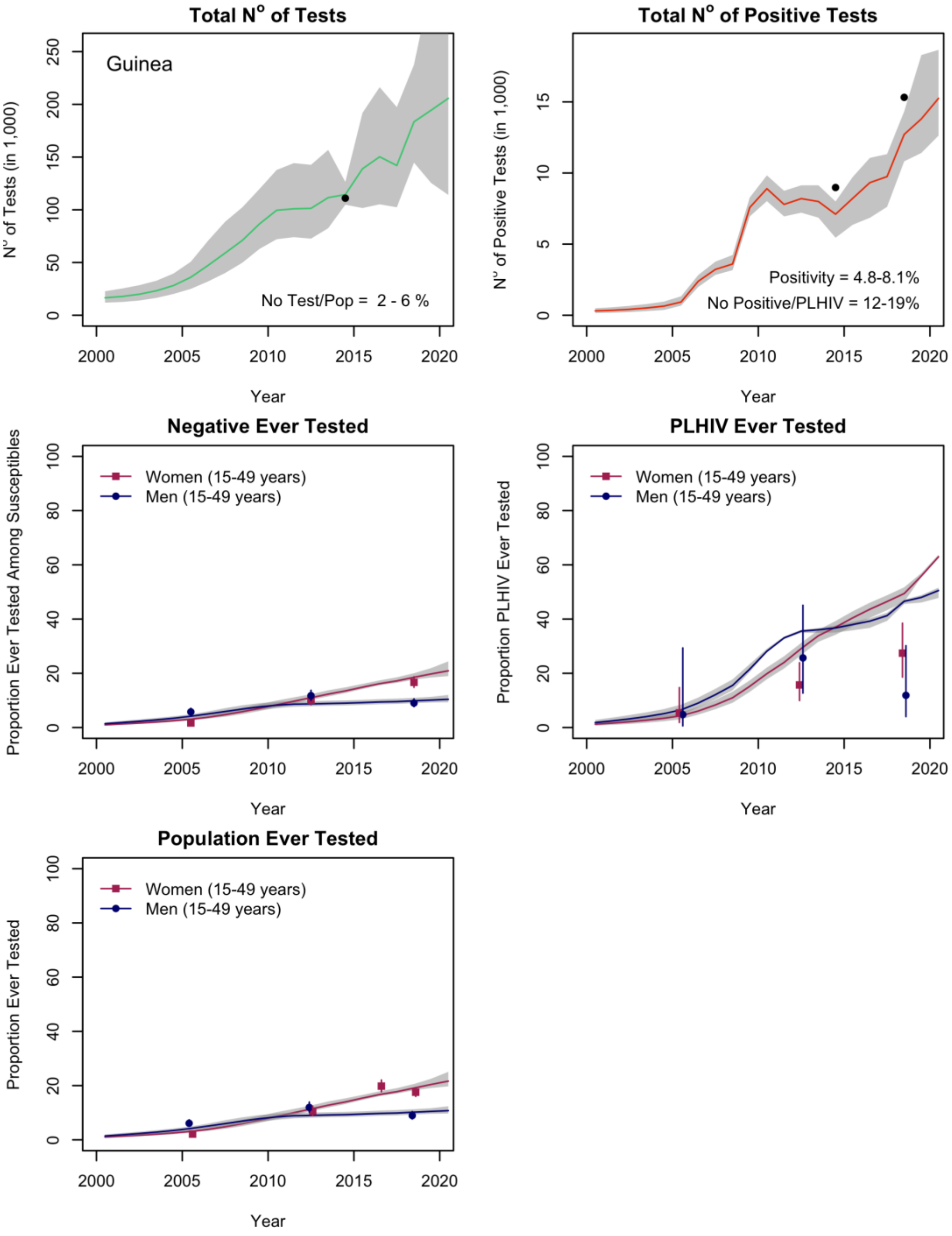
Total N° of Tests
Guinea
N° of Tests (in 1,000)
No Test/Pop = 2 - 6 %
Year
Total N° of Positive Tests
N° of Positive Tests (in 1,000)
Positivity = 4.8-8.1%
No Positive/PLHIV = 12-19%
Year
Negative Ever Tested
Proportion Ever Tested Among Susceptibles
Women (15-49 years)
Men (15-49 years)
Year
PLHIV Ever Tested
Proportion PLHIV Ever Tested
Women (15-49 years)
Men (15-49 years)
Year
Population Ever Tested
Proportion Ever Tested
Women (15-49 years)
Men (15-49 years)
Year

Kenya*

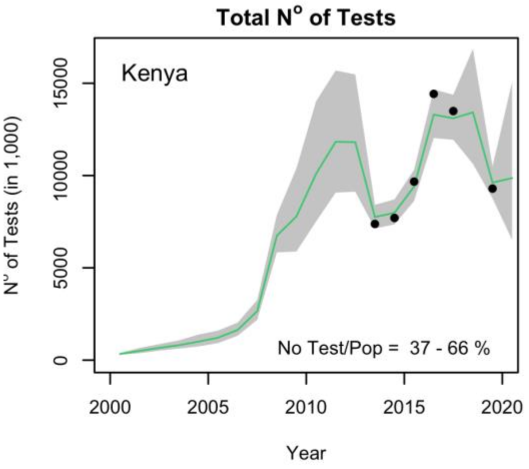
Total N° of Tests
Kenya
N° of Tests (in 1,000)
0
5000
10000
15000
No Test/Pop = 37 - 66 %
2000
2005
2010
2015
2020
Year

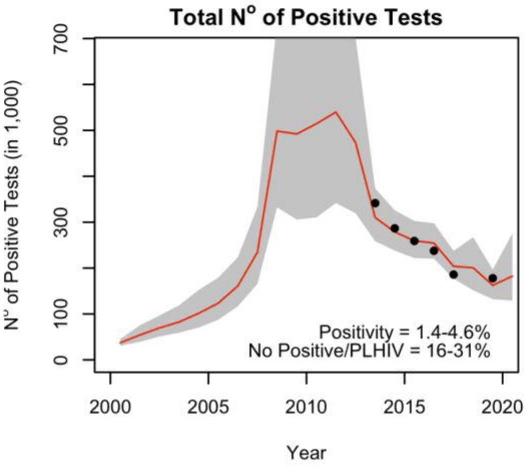
Total N° of Positive Tests
N° of Positive Tests (in 1,000)
0
100
300
500
700
Positivity = 1.4-4.6%
No Positive/PLHIV = 16-31%
2000
2005
2010
2015
2020
Year

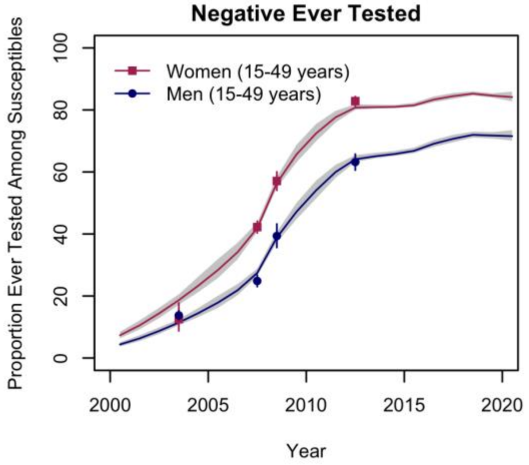
Negative Ever Tested
Proportion Ever Tested Among Susceptibles
0
20
40
60
80
100
Women (15-49 years)
Men (15-49 years)
2000
2005
2010
2015
2020
Year

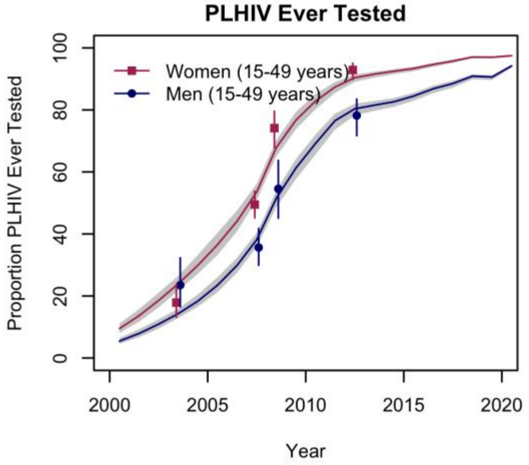
PLHIV Ever Tested
Proportion PLHIV Ever Tested
0
20
40
60
80
100
Women (15-49 years)
Men (15-49 years)
2000
2005
2010
2015
2020
Year

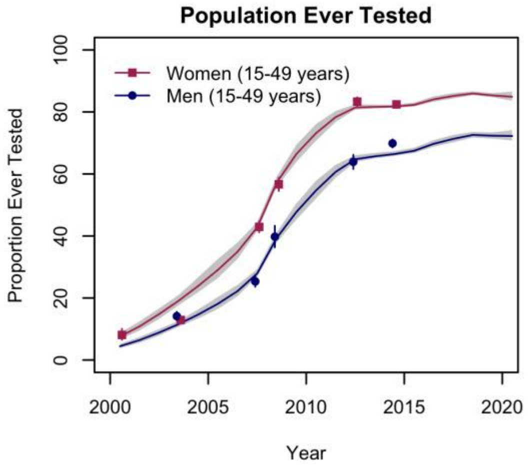
Population Ever Tested
Proportion Ever Tested
0
20
40
60
80
100
Women (15-49 years)
Men (15-49 years)
2000
2005
2010
2015
2020
Year

Lesotho*

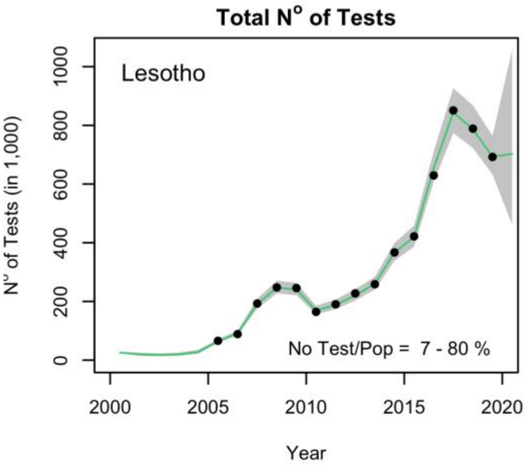
Total N° of Tests
Lesotho
N° of Tests (in 1,000)
No Test/Pop = 7 - 80 %
Year

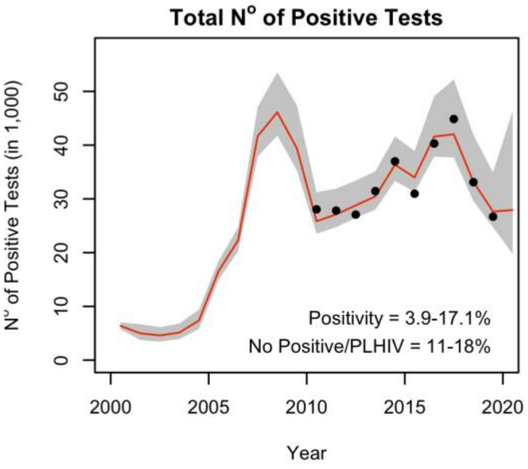
Total N° of Positive Tests
N° of Positive Tests (in 1,000)
Positivity = 3.9-17.1%
No Positive/PLHIV = 11-18%
Year

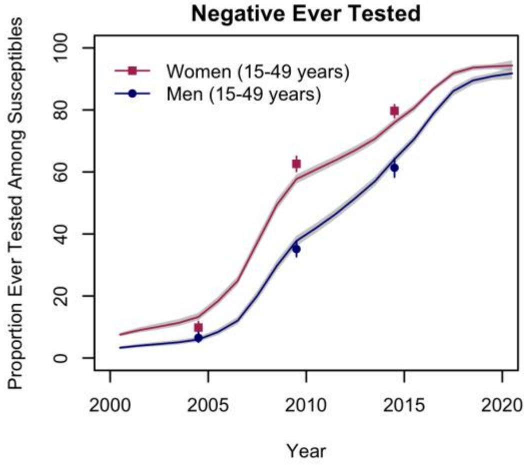
Negative Ever Tested
Proportion Ever Tested Among Susceptibles
Women (15-49 years)
Men (15-49 years)
Year

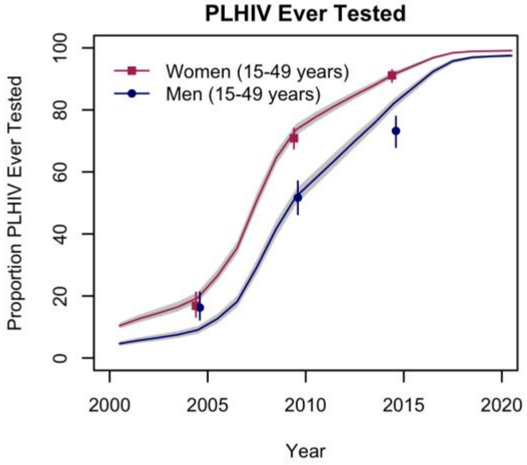
PLHIV Ever Tested
Proportion PLHIV Ever Tested
Women (15-49 years)
Men (15-49 years)
Year

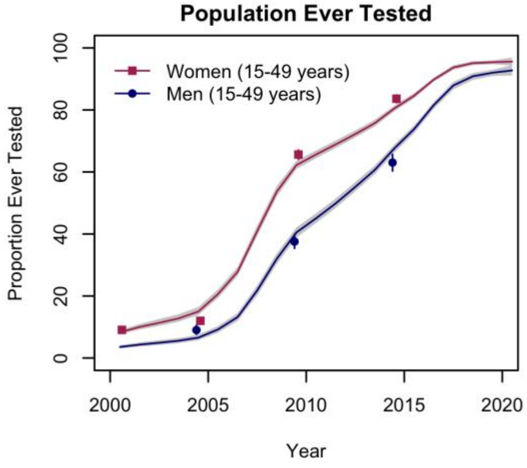
Population Ever Tested
Proportion Ever Tested
Women (15-49 years)
Men (15-49 years)
Year

Liberia

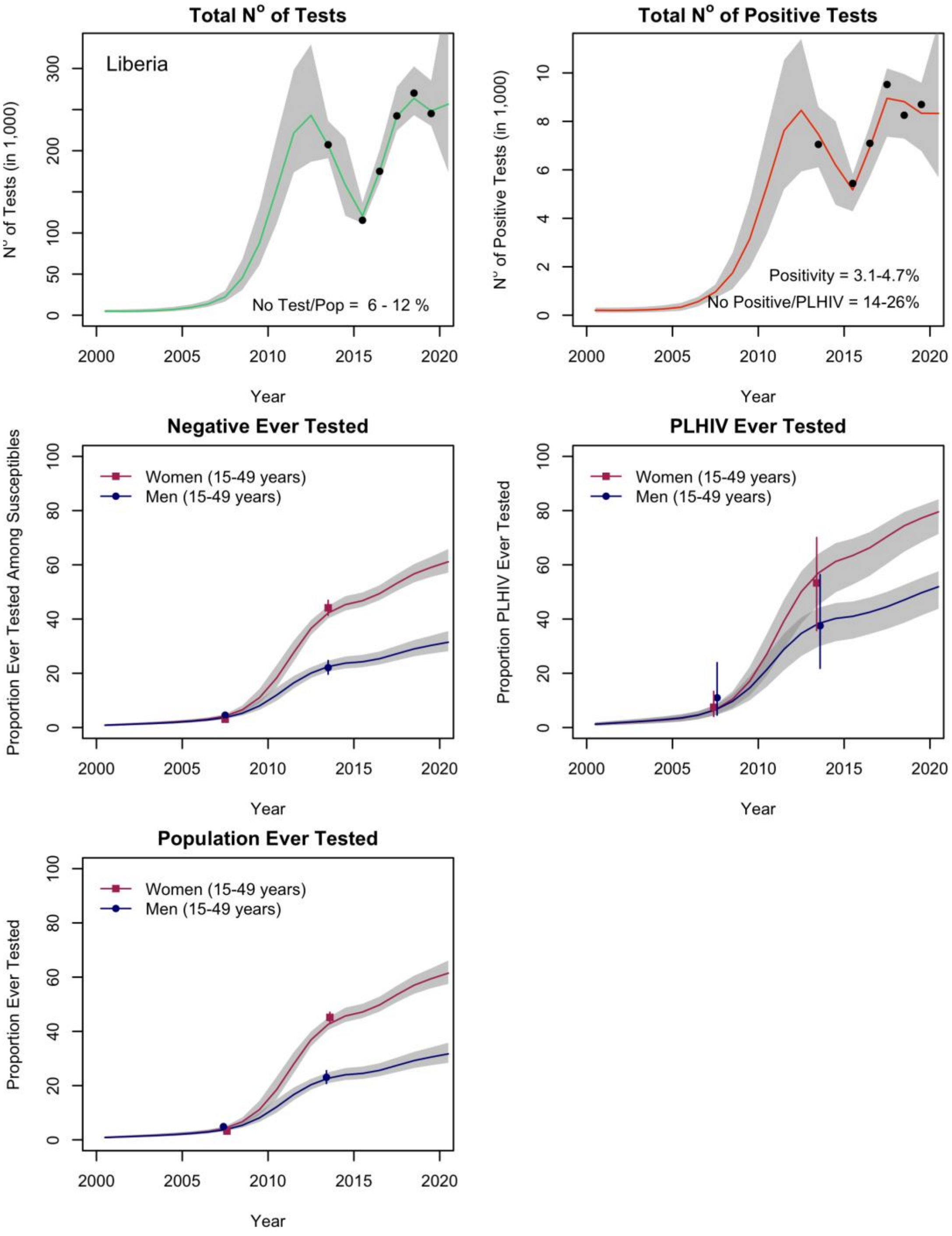
Total N° of Tests
Liberia
No Test/Pop = 6 - 12 %
N° of Tests (in 1,000)
Year
Total N° of Positive Tests
Positivity = 3.1-4.7%
No Positive/PLHIV = 14-26%
N° of Positive Tests (in 1,000)
Year
Negative Ever Tested
Women (15-49 years)
Men (15-49 years)
Proportion Ever Tested Among Susceptibles
Year
PLHIV Ever Tested
Women (15-49 years)
Men (15-49 years)
Proportion PLHIV Ever Tested
Year
Population Ever Tested
Women (15-49 years)
Men (15-49 years)
Proportion Ever Tested
Year

Madagascar

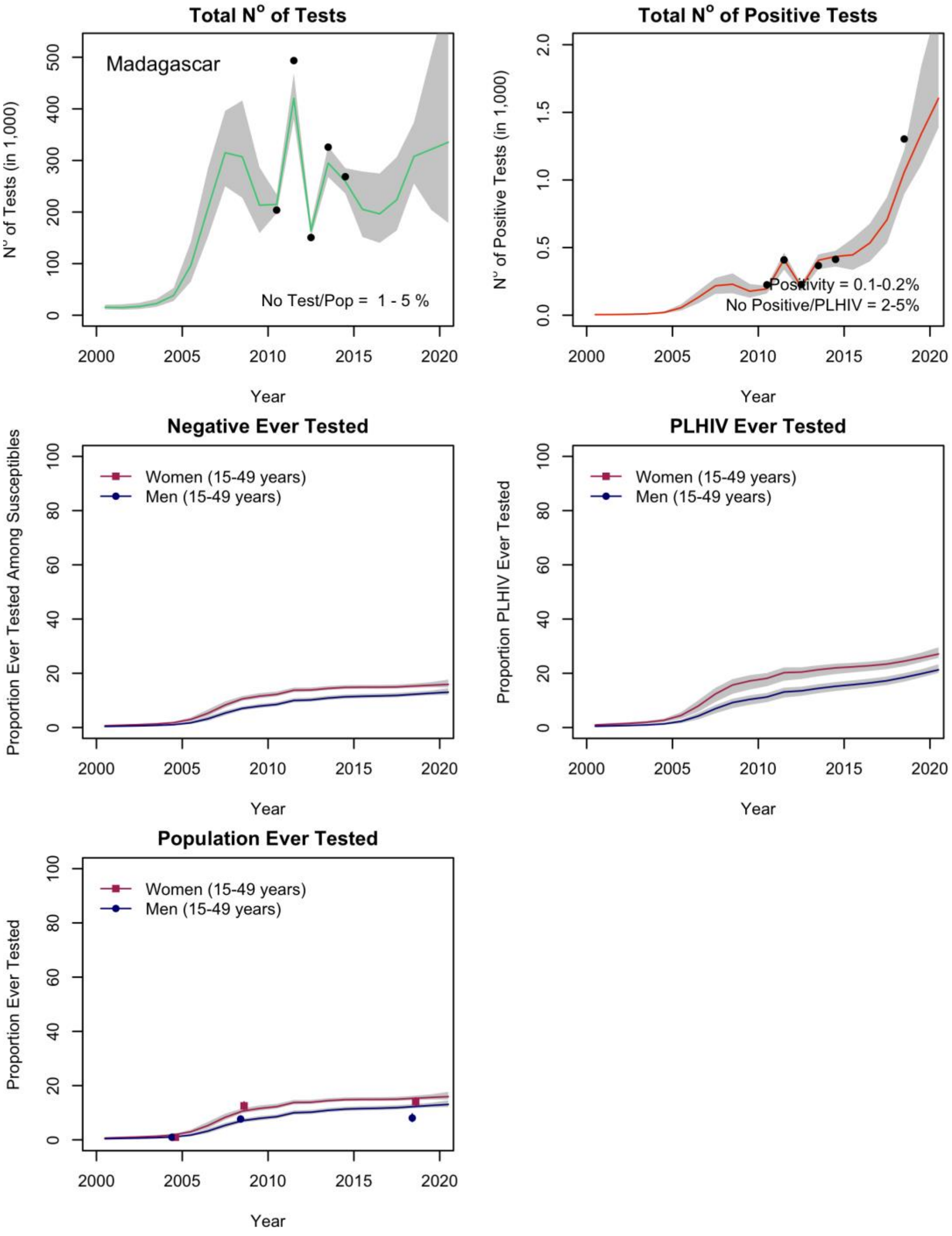

Total N° of Tests
Madagascar
N° of Tests (in 1,000)
No Test/Pop = 1 - 5 %
Year
Total N° of Positive Tests
N° of Positive Tests (in 1,000)
Positivity = 0.1-0.2%
No Positive/PLHIV = 2-5%
Year
Negative Ever Tested
Proportion Ever Tested Among Susceptibles
Women (15-49 years)
Men (15-49 years)
Year
PLHIV Ever Tested
Proportion PLHIV Ever Tested
Women (15-49 years)
Men (15-49 years)
Year
Population Ever Tested
Proportion Ever Tested
Women (15-49 years)
Men (15-49 years)
Year

Malawi

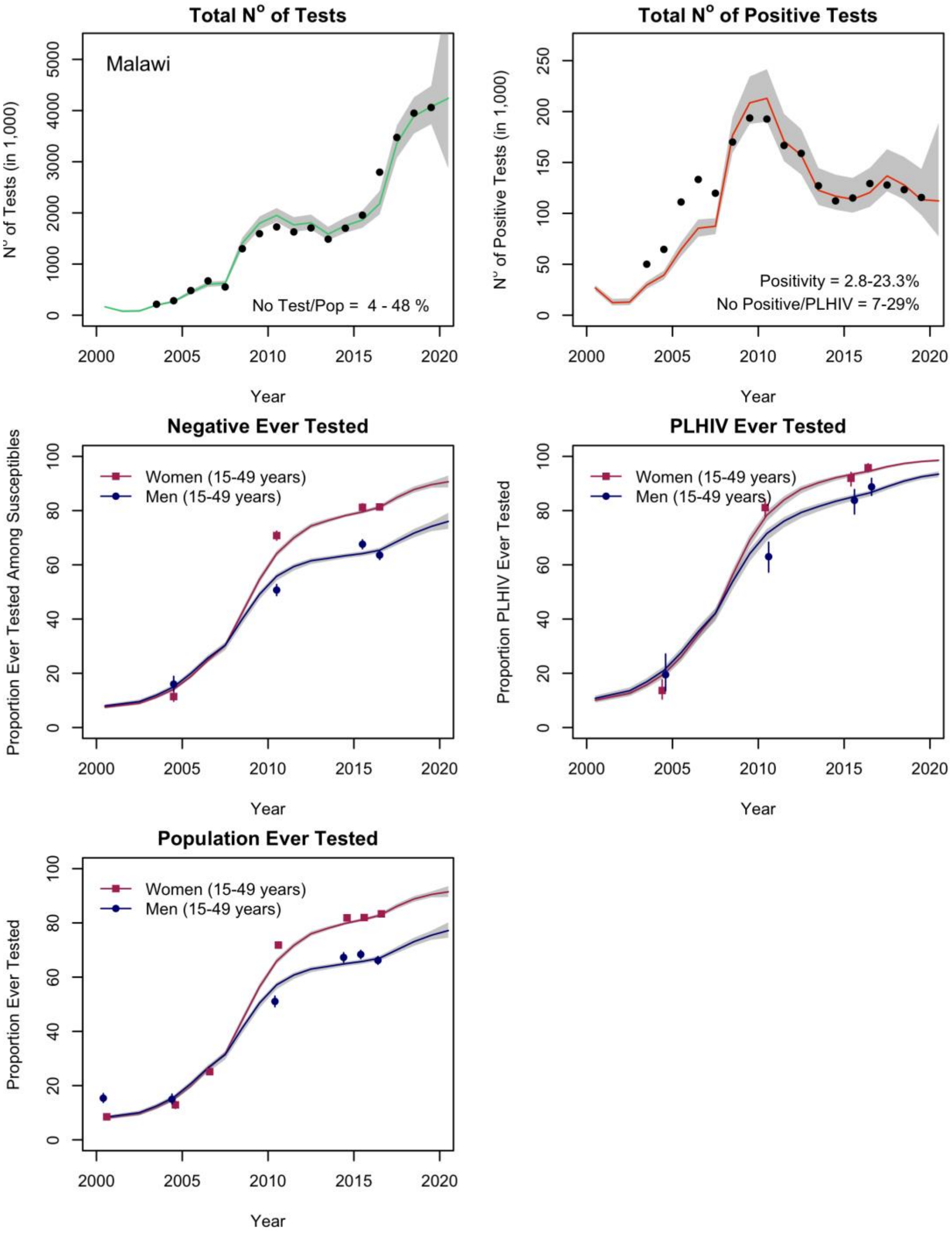
Total N° of Tests
Malawi
N° of Tests (in 1,000)
No Test/Pop = 4 - 48 %
Year
Total N° of Positive Tests
N° of Positive Tests (in 1,000)
Positivity = 2.8-23.3%
No Positive/PLHIV = 7-29%
Year
Negative Ever Tested
Proportion Ever Tested Among Susceptibles
Women (15-49 years)
Men (15-49 years)
Year
PLHIV Ever Tested
Proportion PLHIV Ever Tested
Women (15-49 years)
Men (15-49 years)
Year
Population Ever Tested
Proportion Ever Tested
Women (15-49 years)
Men (15-49 years)
Year

Mali

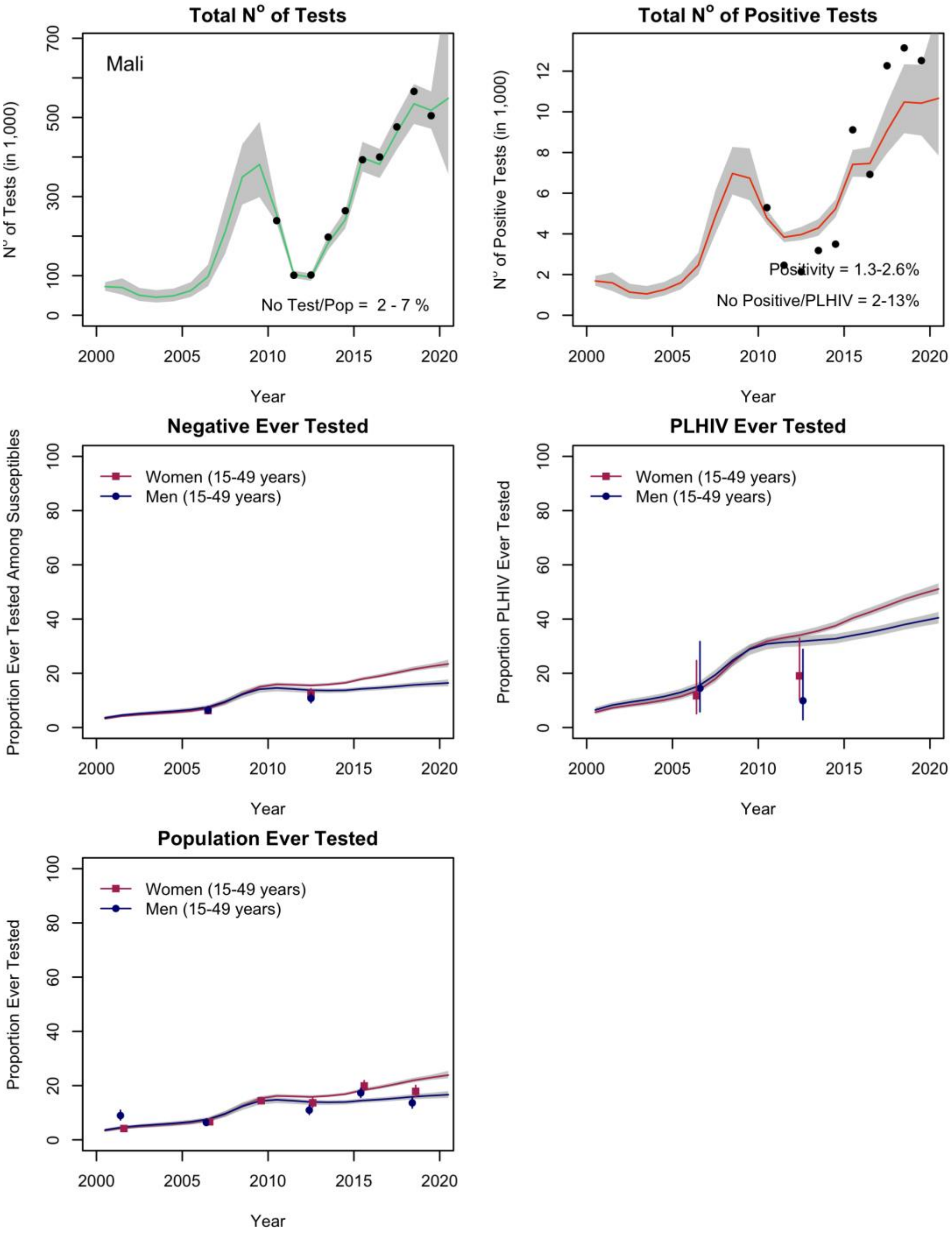

Total N° of Tests
Mali
N° of Tests (in 1,000)
No Test/Pop = 2 - 7 %
Year
Total N° of Positive Tests
N° of Positive Tests (in 1,000)
Positivity = 1.3-2.6%
No Positive/PLHIV = 2-13%
Year
Negative Ever Tested
Proportion Ever Tested Among Susceptibles
Women (15-49 years)
Men (15-49 years)
Year
PLHIV Ever Tested
Proportion PLHIV Ever Tested
Women (15-49 years)
Men (15-49 years)
Year
Population Ever Tested
Proportion Ever Tested
Women (15-49 years)
Men (15-49 years)
Year

Mauritania

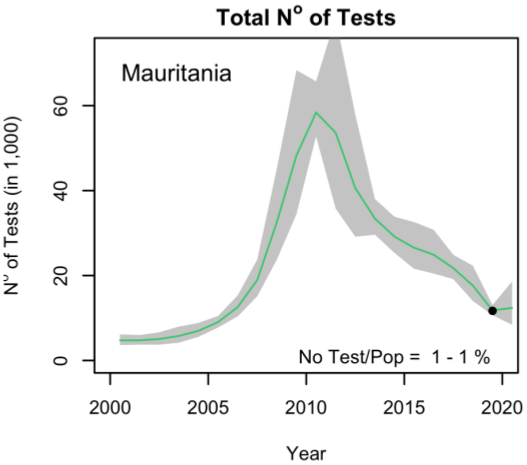

Total N° of Tests
Mauritania
N° of Tests (in 1,000)
No Test/Pop = 1 - 1 %
Year

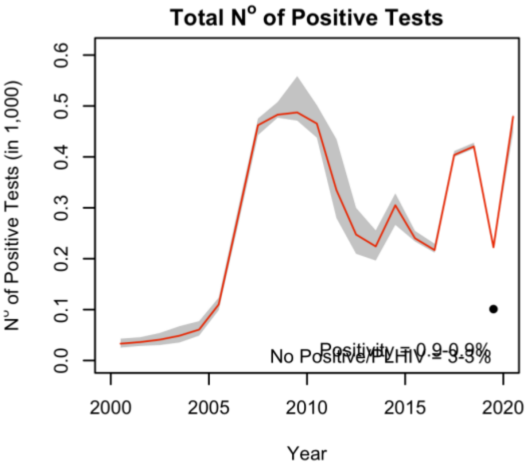

Total N° of Positive Tests
N° of Positive Tests (in 1,000)
Year

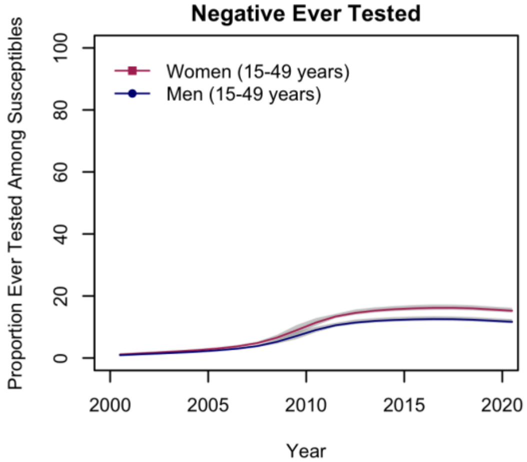

Negative Ever Tested
Proportion Ever Tested Among Susceptibles
Women (15-49 years)
Men (15-49 years)
Year

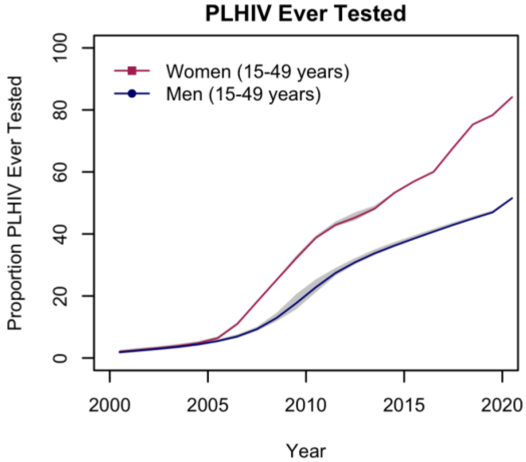

PLHIV Ever Tested
Proportion PLHIV Ever Tested
Women (15-49 years)
Men (15-49 years)
Year

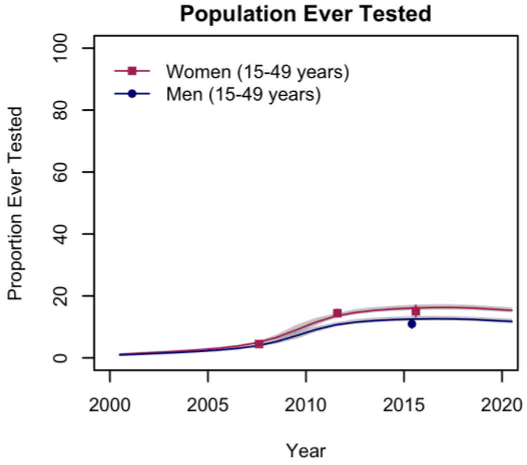

Population Ever Tested
Proportion Ever Tested
Women (15-49 years)
Men (15-49 years)
Year

Mozambique

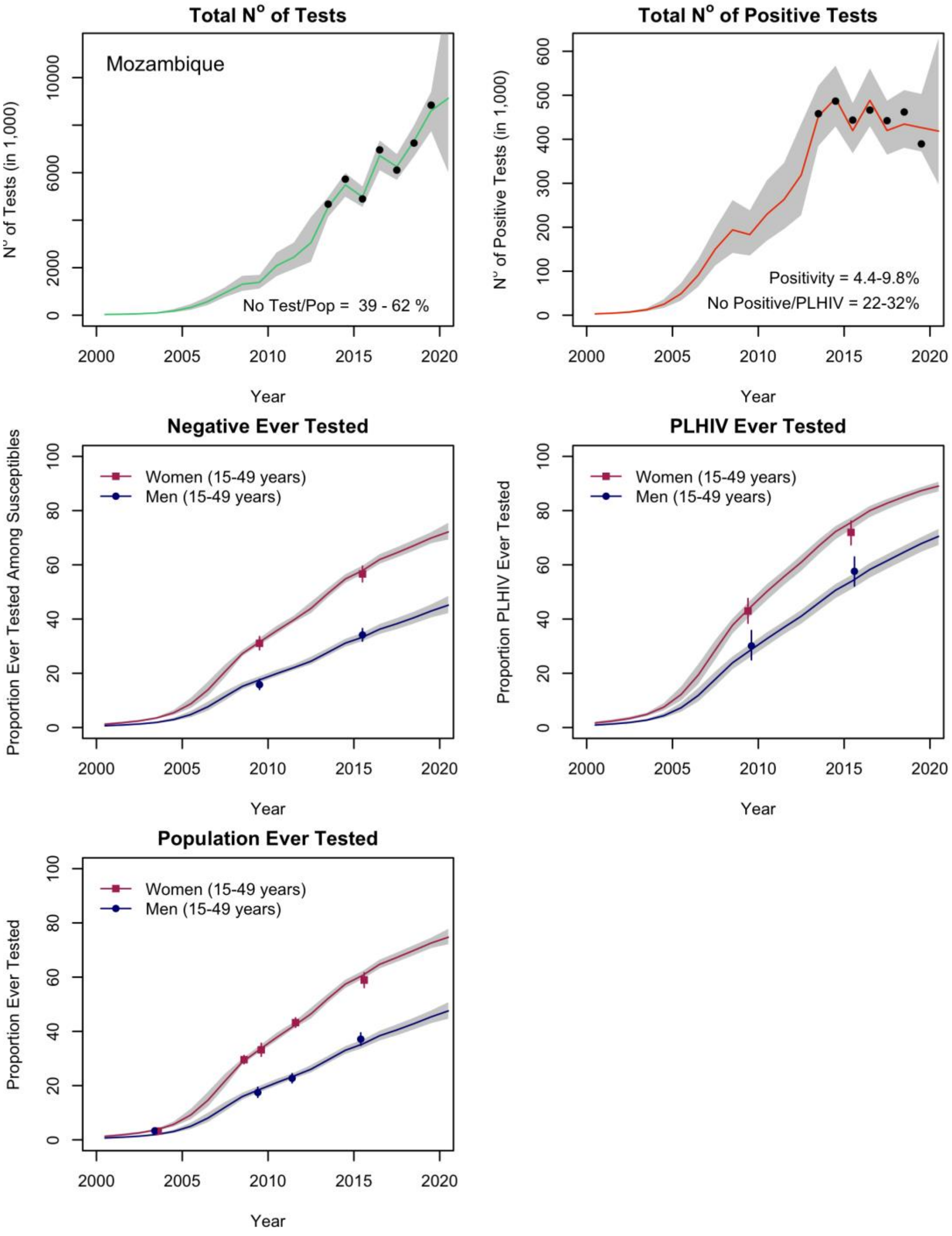
Total N° of Tests
Mozambique
No Test/Pop = 39 - 62 %
N° of Tests (in 1,000)
Year
Total N° of Positive Tests
Positivity = 4.4-9.8%
No Positive/PLHIV = 22-32%
N° of Positive Tests (in 1,000)
Year
Negative Ever Tested
Women (15-49 years)
Men (15-49 years)
Proportion Ever Tested Among Susceptibles
Year
PLHIV Ever Tested
Women (15-49 years)
Men (15-49 years)
Proportion PLHIV Ever Tested
Year
Population Ever Tested
Women (15-49 years)
Men (15-49 years)
Proportion Ever Tested
Year

Namibia*

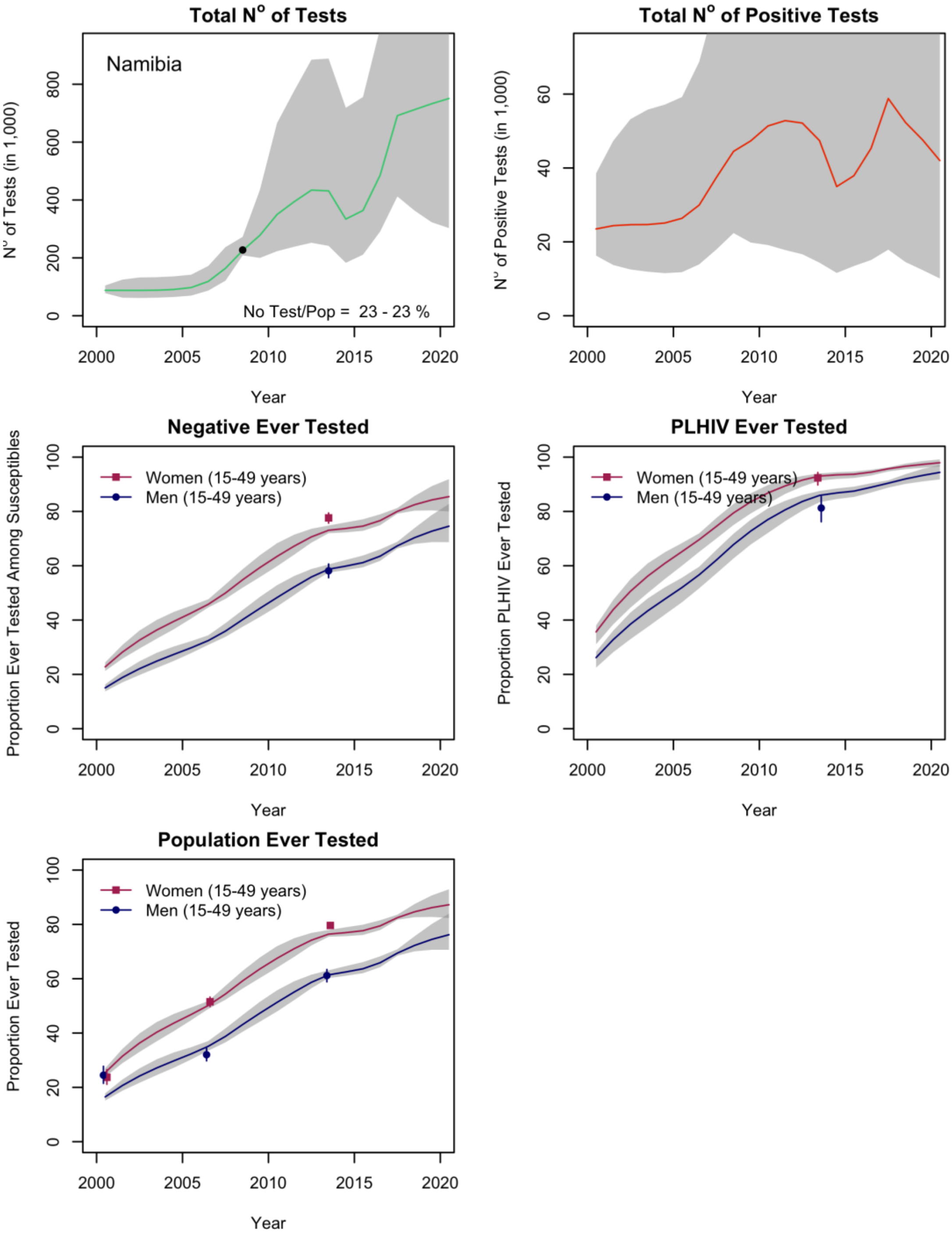

Total N° of Tests
Namibia
No Test/Pop = 23 - 23 %
N° of Tests (in 1,000)
Year
Total N° of Positive Tests
N° of Positive Tests (in 1,000)
Year
Negative Ever Tested
Women (15-49 years)
Men (15-49 years)
Proportion Ever Tested Among Susceptibles
Year
PLHIV Ever Tested
Women (15-49 years)
Men (15-49 years)
Proportion PLHIV Ever Tested
Year
Population Ever Tested
Women (15-49 years)
Men (15-49 years)
Proportion Ever Tested
Year

Niger

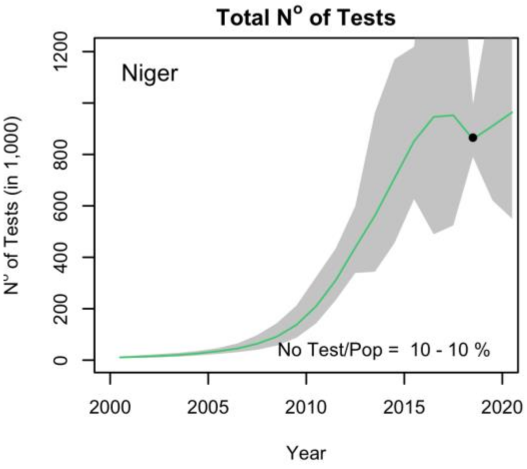
Total N° of Tests
Niger
N° of Tests (in 1,000)
0
200
400
600
800
1000
1200
No Test/Pop = 10 - 10 %
2000
2005
2010
2015
2020
Year

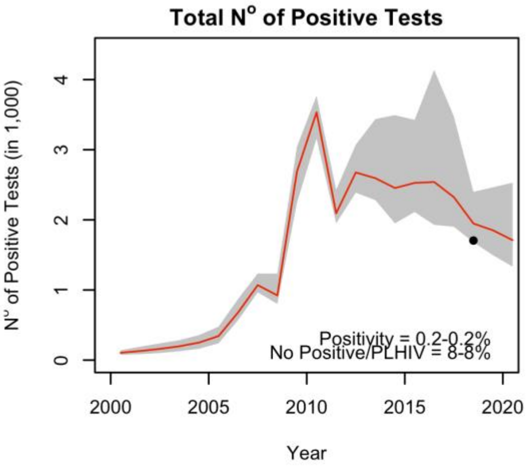
Total N° of Positive Tests
N° of Positive Tests (in 1,000)
0
1
2
3
4
Positivity = 0.2-0.2%
No Positive/PLHIV = 8-8%
2000
2005
2010
2015
2020
Year

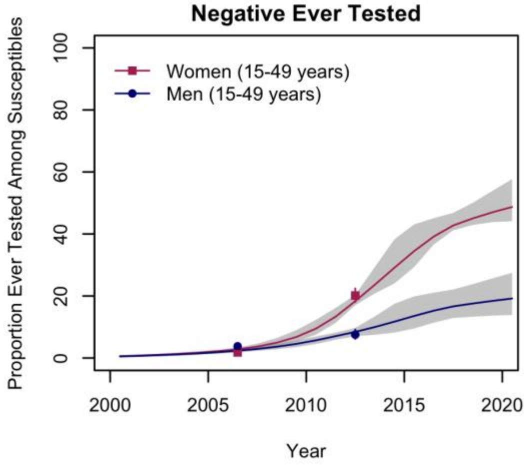
Negative Ever Tested
Proportion Ever Tested Among Susceptibles
0
20
40
60
80
100
Women (15-49 years)
Men (15-49 years)
2000
2005
2010
2015
2020
Year

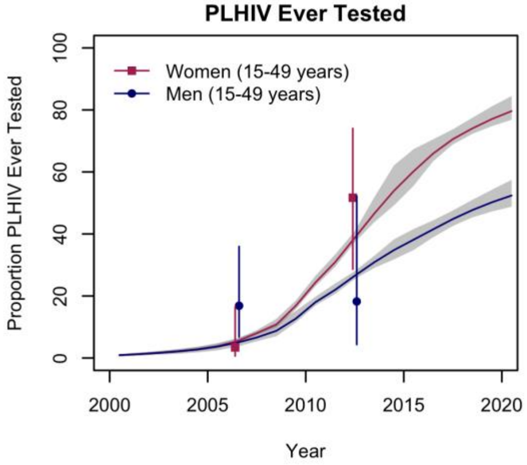
PLHIV Ever Tested
Proportion PLHIV Ever Tested
0
20
40
60
80
100
Women (15-49 years)
Men (15-49 years)
2000
2005
2010
2015
2020
Year

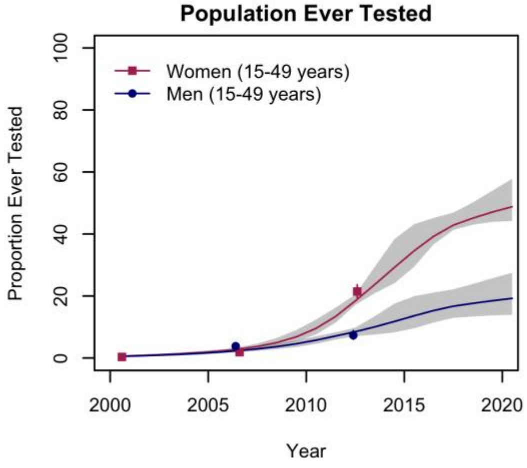
Population Ever Tested
Proportion Ever Tested
0
20
40
60
80
100
Women (15-49 years)
Men (15-49 years)
2000
2005
2010
2015
2020
Year

Nigeria

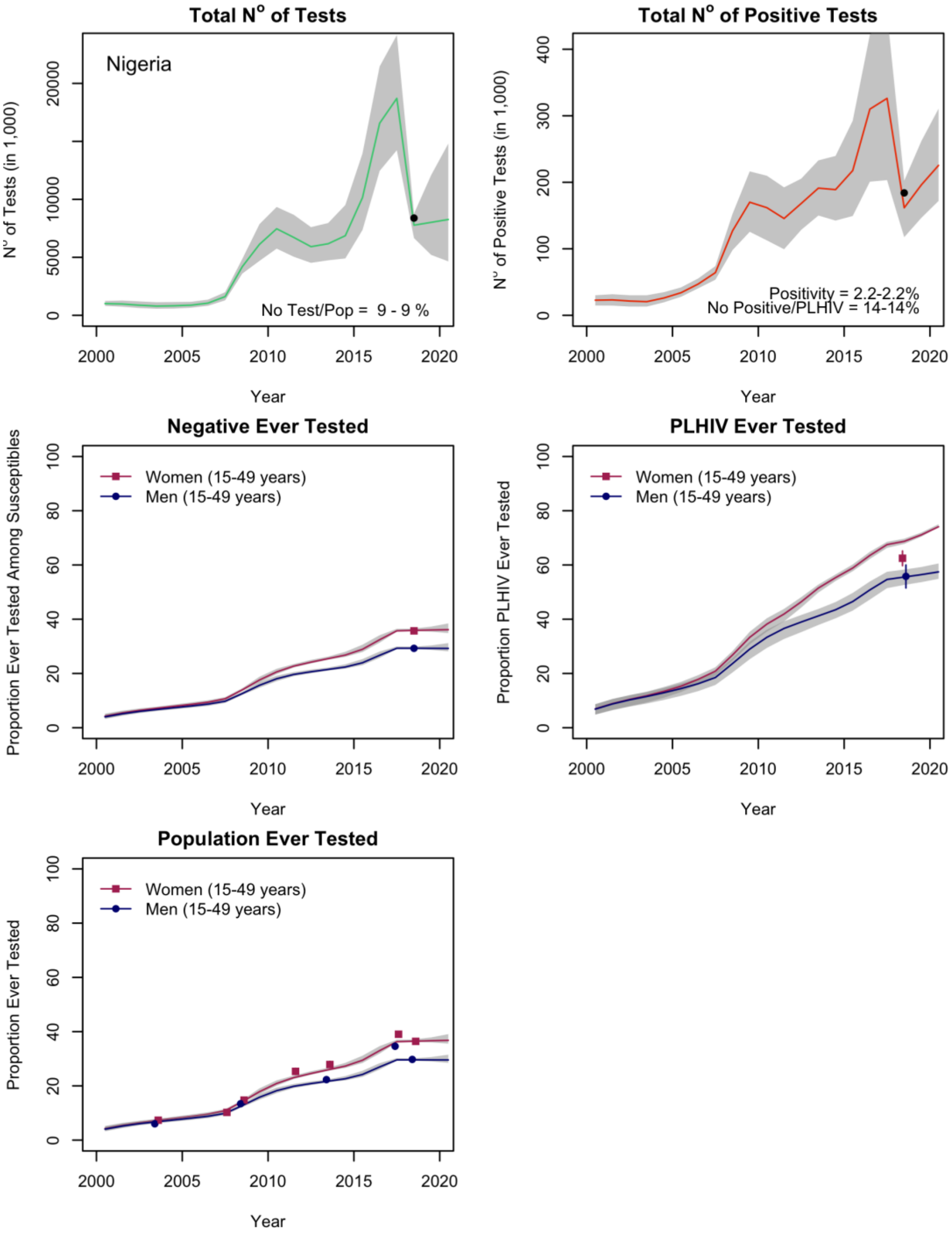
Total N° of Tests
Nigeria
N° of Tests (in 1,000)
No Test/Pop = 9 - 9 %
Year
Total N° of Positive Tests
N° of Positive Tests (in 1,000)
Positivity = 2.2-2.2%
No Positive/PLHIV = 14-14%
Year
Negative Ever Tested
Proportion Ever Tested Among Susceptibles
Women (15-49 years)
Men (15-49 years)
Year
PLHIV Ever Tested
Proportion PLHIV Ever Tested
Women (15-49 years)
Men (15-49 years)
Year
Population Ever Tested
Proportion Ever Tested
Women (15-49 years)
Men (15-49 years)
Year

Rwanda*

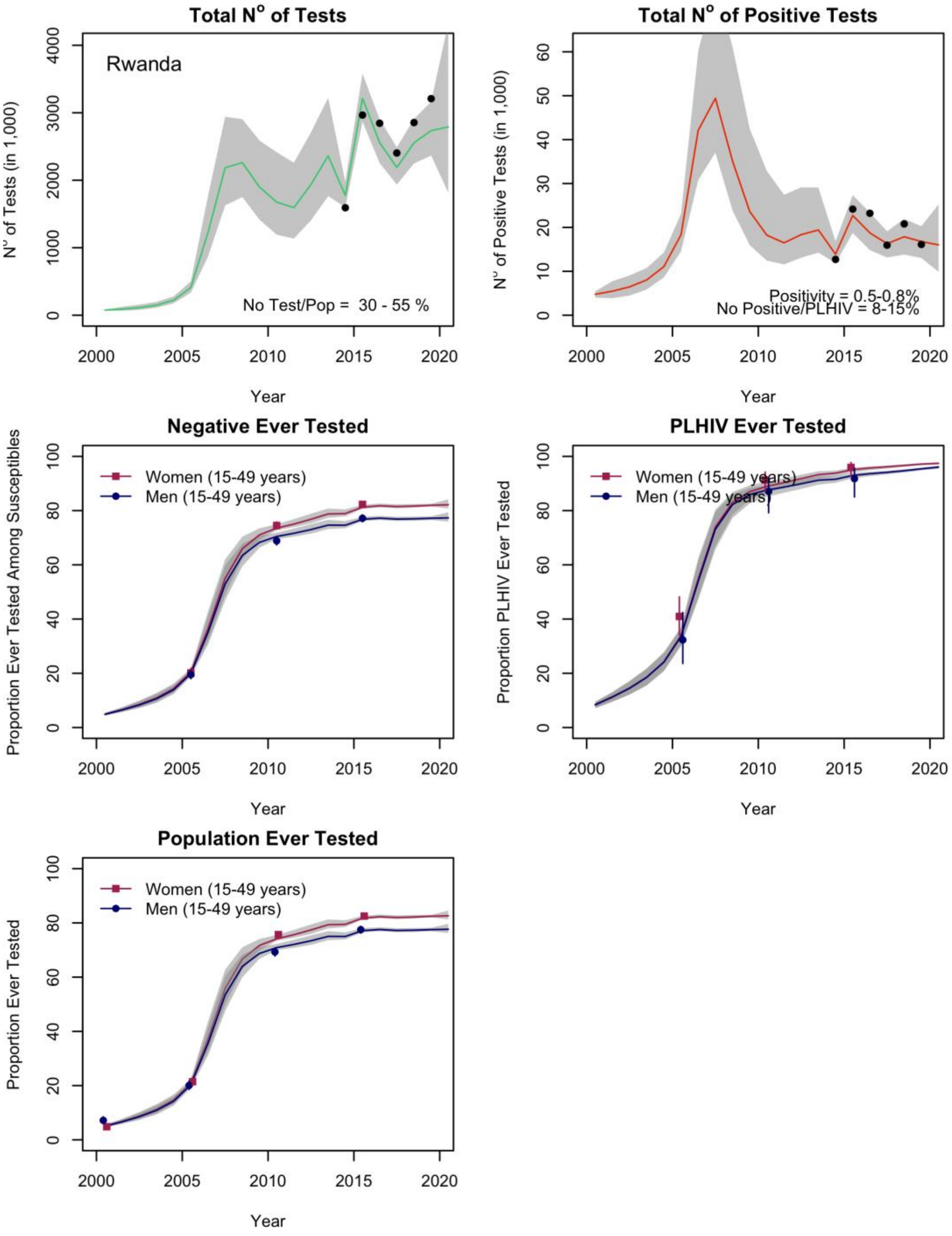
Total N° of Tests
Rwanda
N° of Tests (in 1,000)
No Test/Pop = 30 - 55 %
Year
Total N° of Positive Tests
N° of Positive Tests (in 1,000)
Positivity = 0.5-0.8%
No Positive/PLHIV = 8-15%
Year
Negative Ever Tested
Proportion Ever Tested Among Susceptibles
Women (15-49 years)
Men (15-49 years)
Year
PLHIV Ever Tested
Proportion PLHIV Ever Tested
Women (15-49 years)
Men (15-49 years)
Year
Population Ever Tested
Proportion Ever Tested
Women (15-49 years)
Men (15-49 years)
Year

Senegal

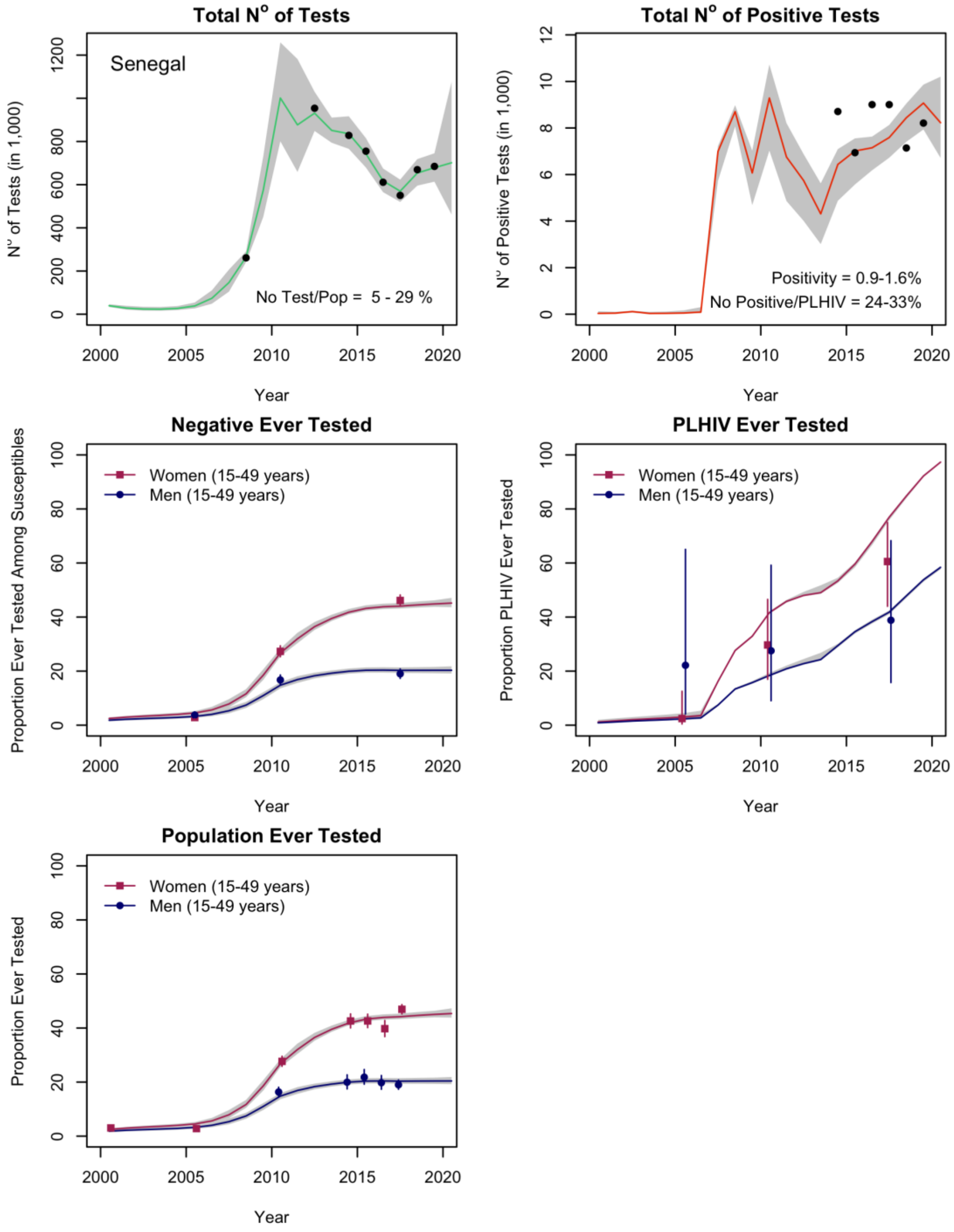
Total N° of Tests
Senegal
N° of Tests (in 1,000)
No Test/Pop = 5 - 29 %
Year
Total N° of Positive Tests
N° of Positive Tests (in 1,000)
Positivity = 0.9-1.6%
No Positive/PLHIV = 24-33%
Year
Negative Ever Tested
Proportion Ever Tested Among Susceptibles
Women (15-49 years)
Men (15-49 years)
Year
PLHIV Ever Tested
Proportion PLHIV Ever Tested
Women (15-49 years)
Men (15-49 years)
Year
Population Ever Tested
Proportion Ever Tested
Women (15-49 years)
Men (15-49 years)
Year

Sierra Leone

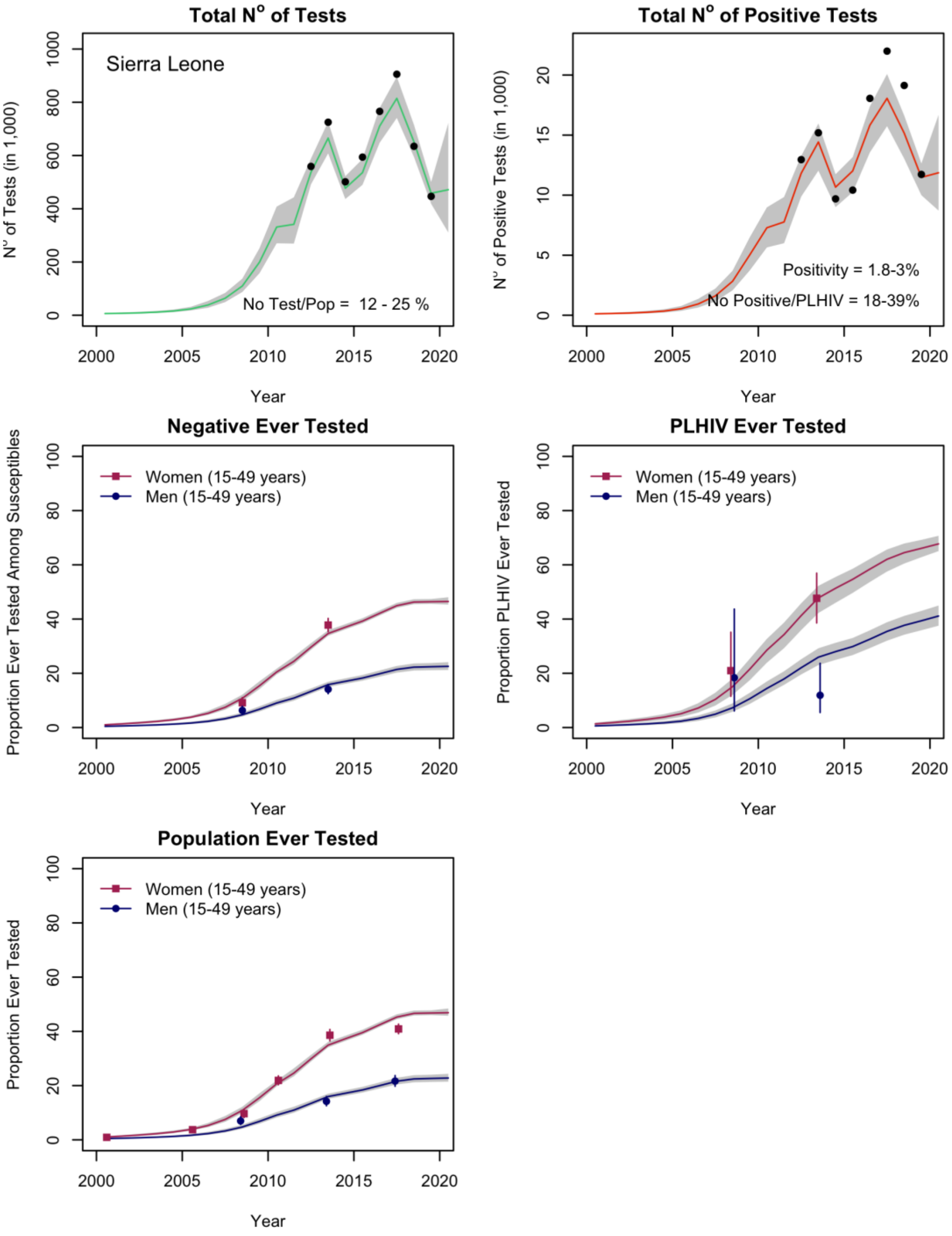

South Africa

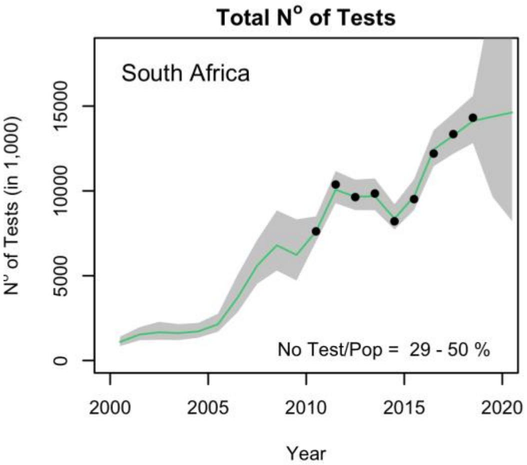

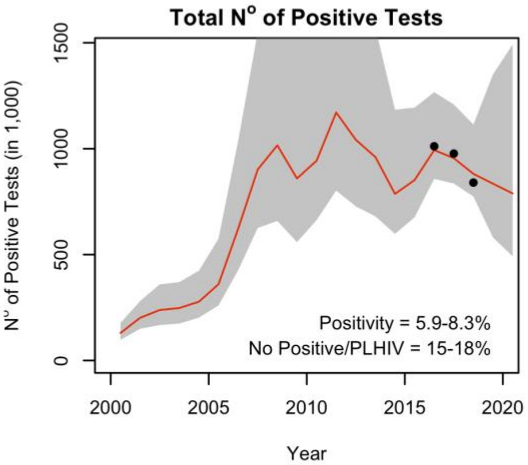

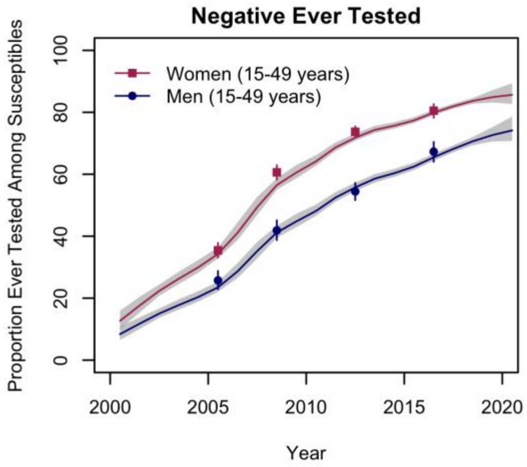

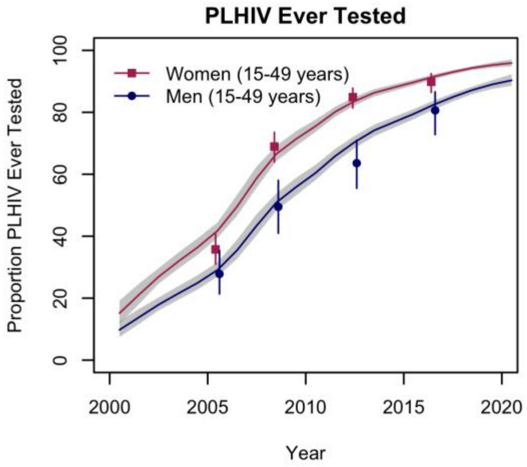

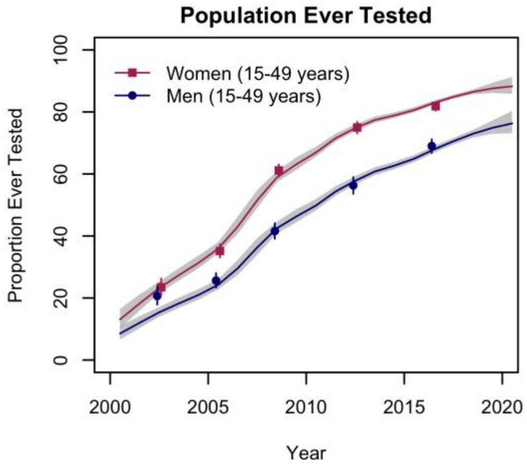

South Sudan

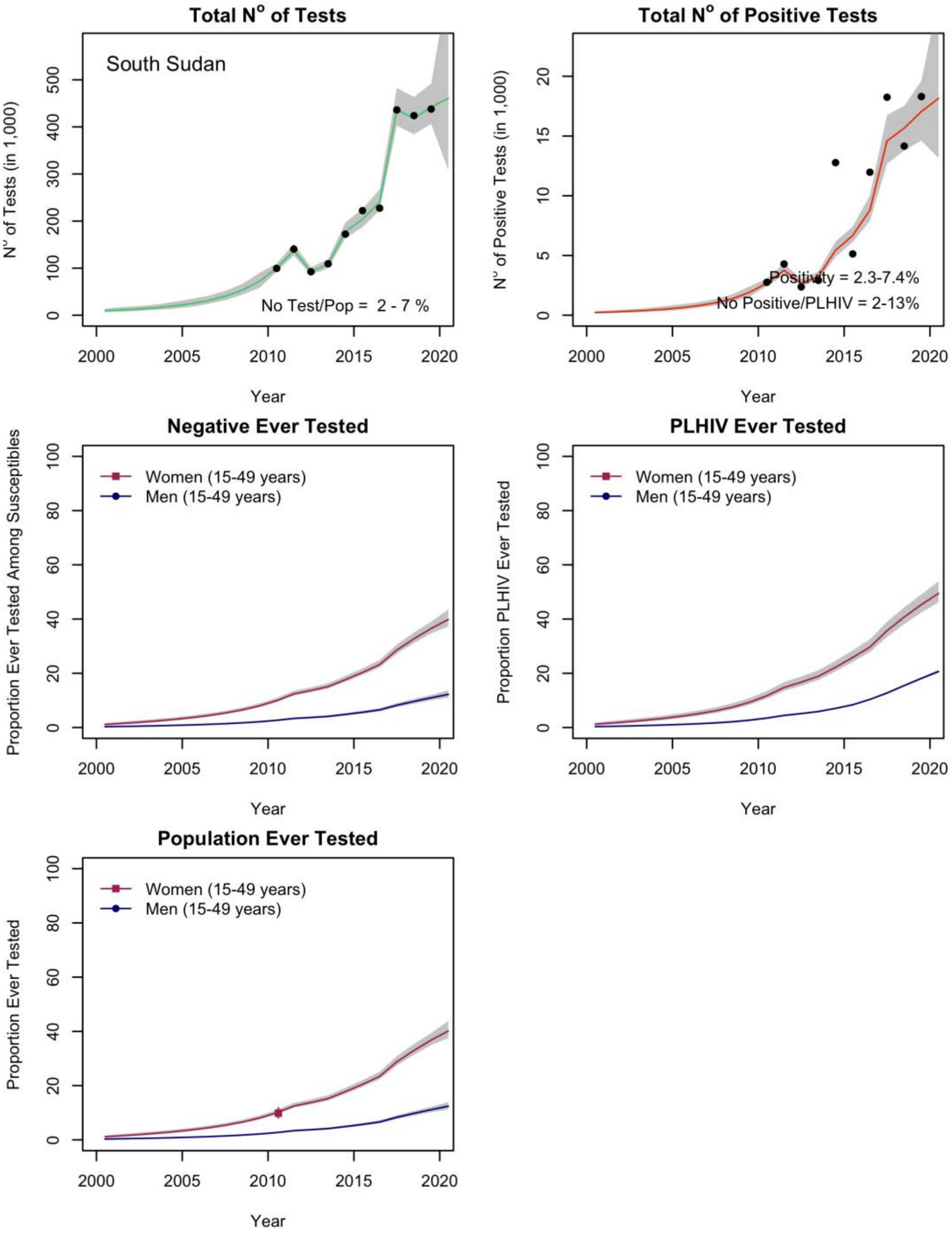
Total N° of Tests
South Sudan
N° of Tests (in 1,000)
No Test/Pop = 2 - 7 %
Year
Total N° of Positive Tests
N° of Positive Tests (in 1,000)
Positivity = 2.3-7.4%
No Positive/PLHIV = 2-13%
Year
Negative Ever Tested
Proportion Ever Tested Among Susceptibles
Women (15-49 years)
Men (15-49 years)
Year
PLHIV Ever Tested
Proportion PLHIV Ever Tested
Women (15-49 years)
Men (15-49 years)
Year
Population Ever Tested
Proportion Ever Tested
Women (15-49 years)
Men (15-49 years)
Year

Tanzania

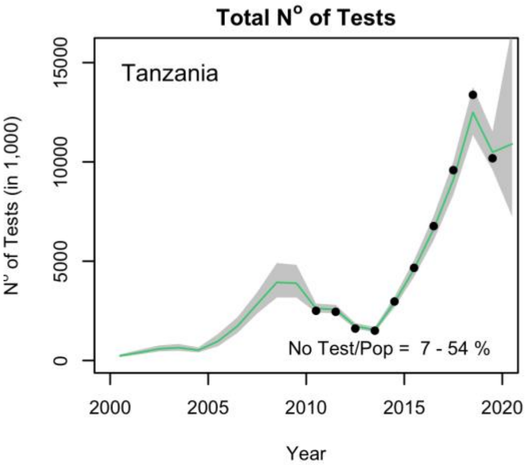
Total N° of Tests
Tanzania
N° of Tests (in 1,000)
No Test/Pop = 7 - 54 %
Year

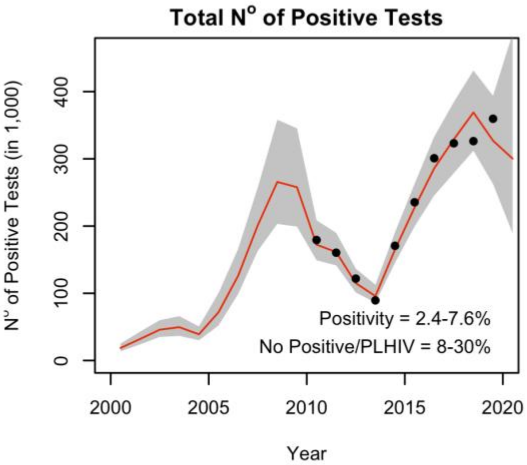
Total N° of Positive Tests
N° of Positive Tests (in 1,000)
Positivity = 2.4-7.6%
No Positive/PLHIV = 8-30%
Year

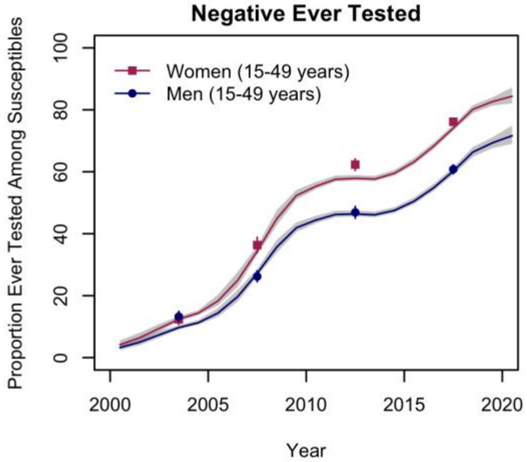
Negative Ever Tested
Proportion Ever Tested Among Susceptibles
Women (15-49 years)
Men (15-49 years)
Year

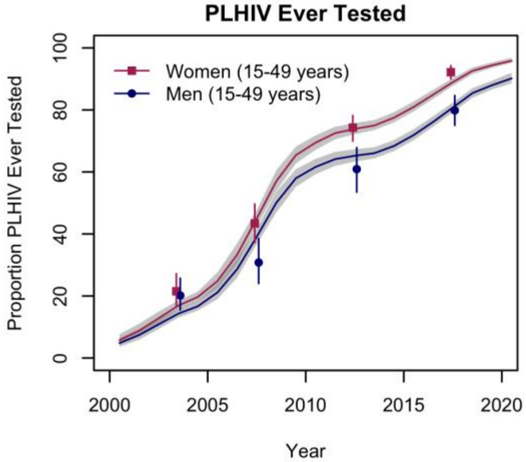
PLHIV Ever Tested
Proportion PLHIV Ever Tested
Women (15-49 years)
Men (15-49 years)
Year

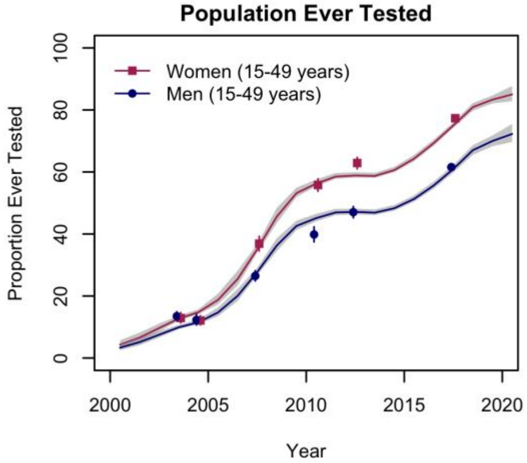
Population Ever Tested
Proportion Ever Tested
Women (15-49 years)
Men (15-49 years)
Year

Togo

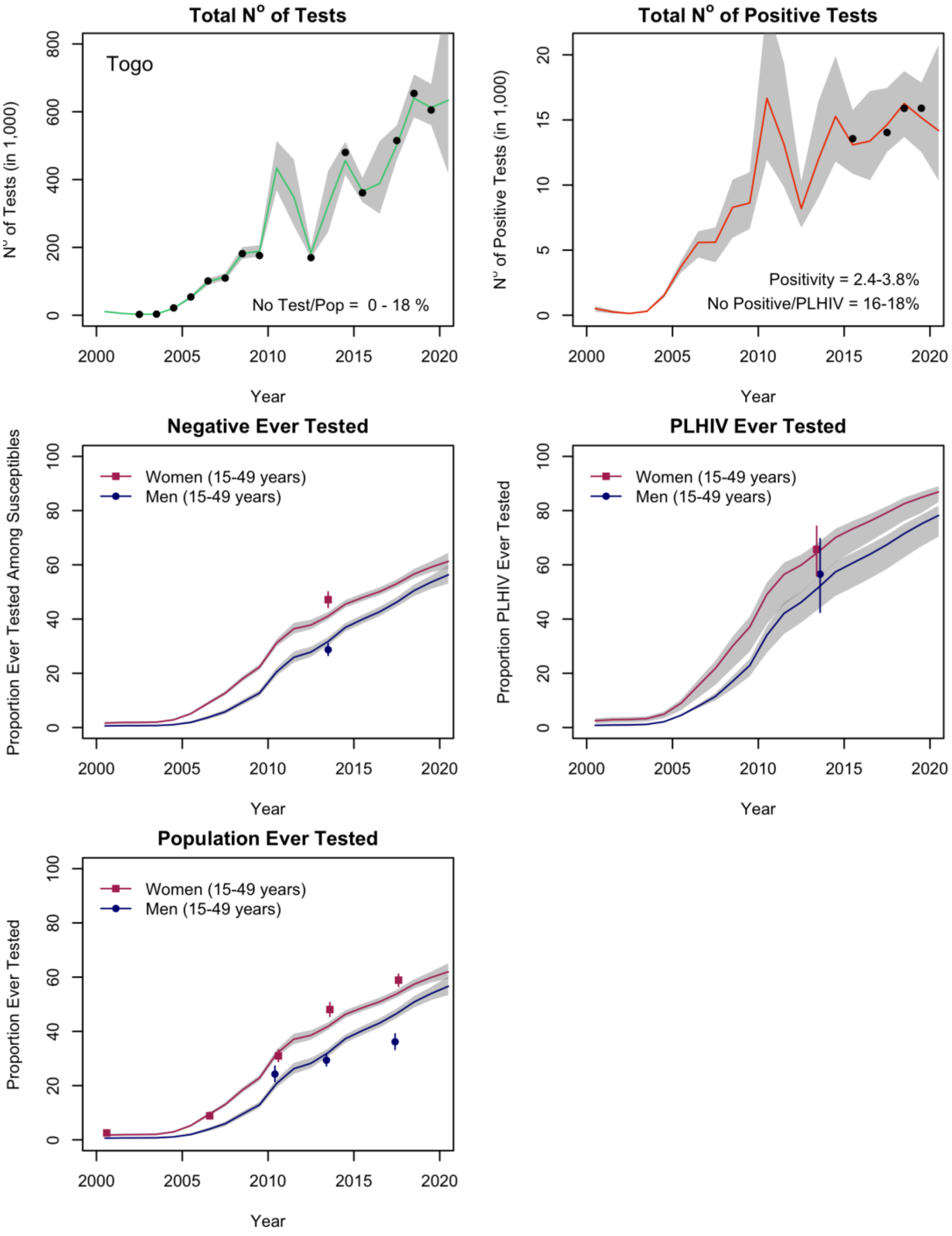
Total N° of Tests
Togo
N° of Tests (in 1,000)
No Test/Pop = 0 - 18 %
Year
Total N° of Positive Tests
N° of Positive Tests (in 1,000)
Positivity = 2.4-3.8%
No Positive/PLHIV = 16-18%
Year
Negative Ever Tested
Proportion Ever Tested Among Susceptibles
Women (15-49 years)
Men (15-49 years)
Year
PLHIV Ever Tested
Proportion PLHIV Ever Tested
Women (15-49 years)
Men (15-49 years)
Year
Population Ever Tested
Proportion Ever Tested
Women (15-49 years)
Men (15-49 years)
Year

Uganda*

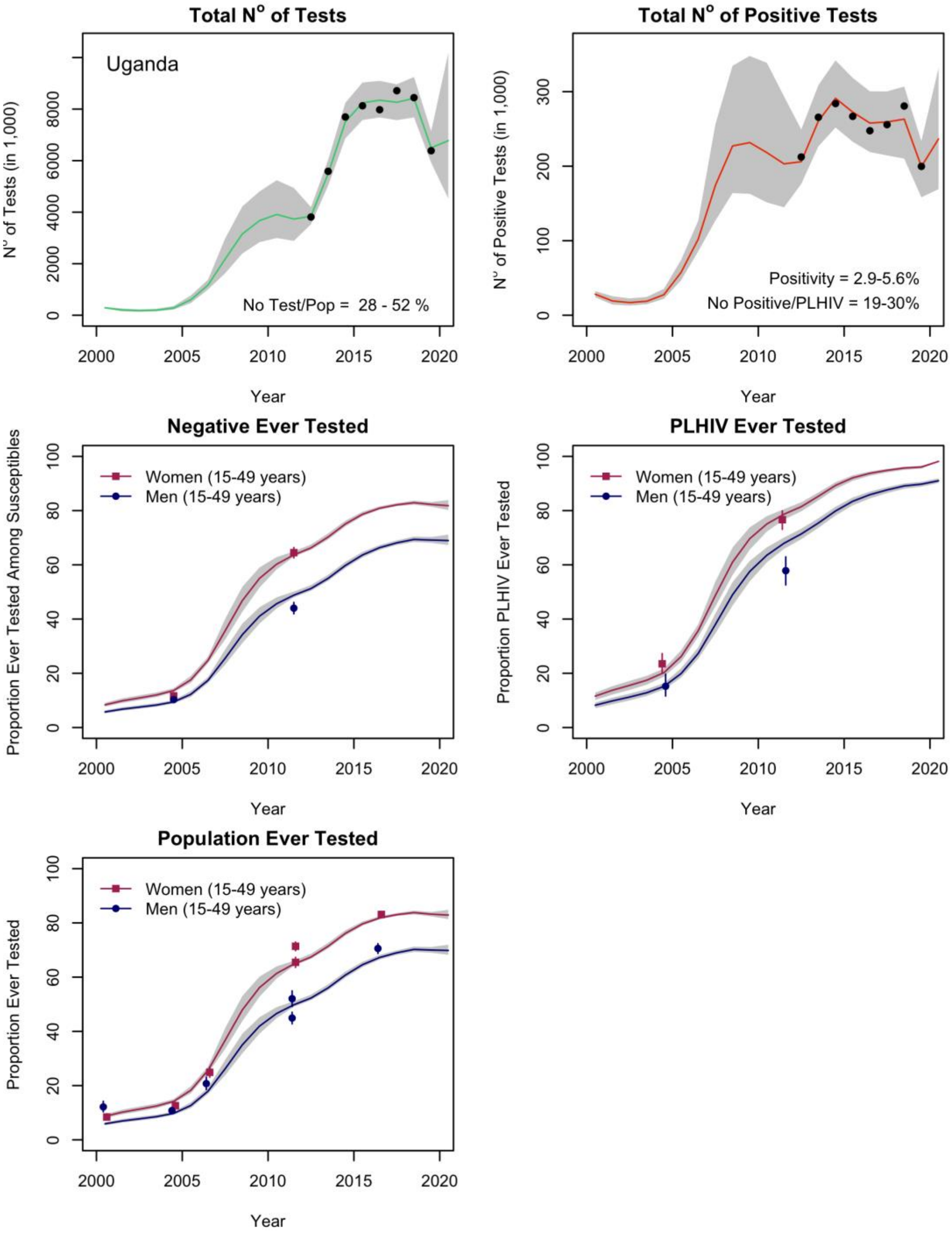
Total N° of Tests
Uganda
N° of Tests (in 1,000)
No Test/Pop = 28 - 52 %
Year
Total N° of Positive Tests
N° of Positive Tests (in 1,000)
Positivity = 2.9-5.6%
No Positive/PLHIV = 19-30%
Year
Negative Ever Tested
Proportion Ever Tested Among Susceptibles
Women (15-49 years)
Men (15-49 years)
Year
PLHIV Ever Tested
Proportion PLHIV Ever Tested
Women (15-49 years)
Men (15-49 years)
Year
Population Ever Tested
Proportion Ever Tested
Women (15-49 years)
Men (15-49 years)
Year

Zambia

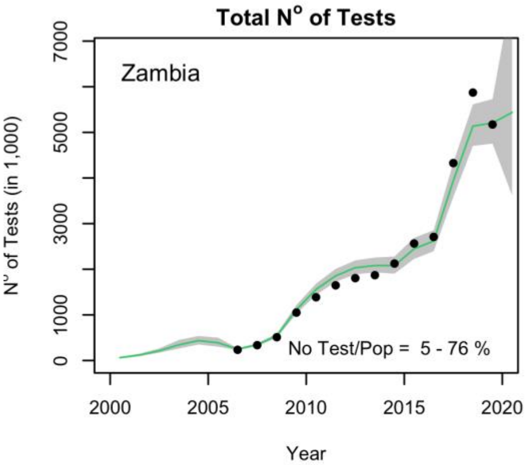
Total N° of Tests
Zambia
N° of Tests (in 1,000)
No Test/Pop = 5 - 76 %
Year

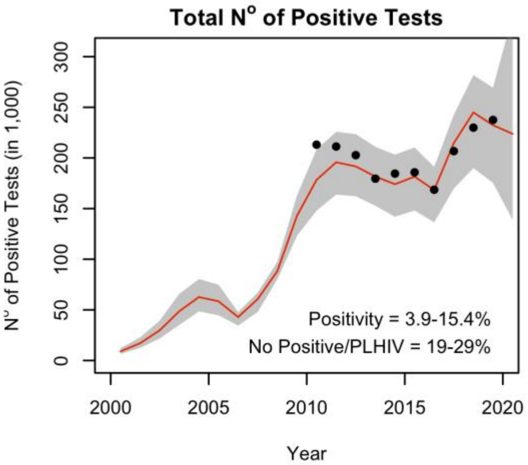
Total N° of Positive Tests
N° of Positive Tests (in 1,000)
Positivity = 3.9-15.4%
No Positive/PLHIV = 19-29%
Year

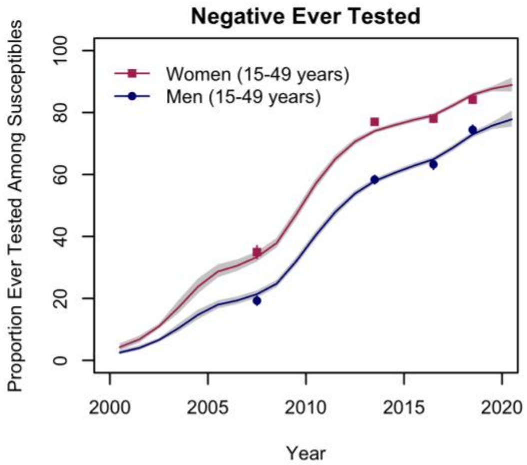
Negative Ever Tested
Proportion Ever Tested Among Susceptibles
Women (15-49 years)
Men (15-49 years)
Year

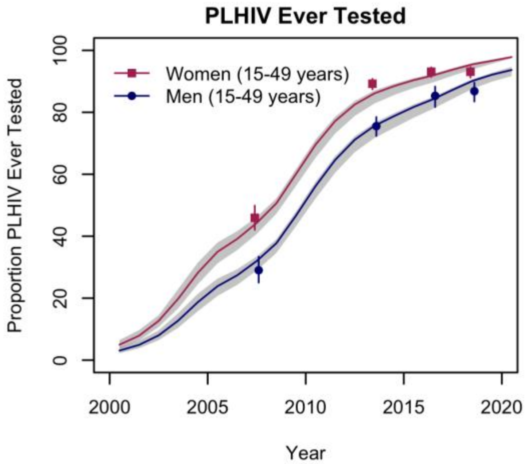
PLHIV Ever Tested
Proportion PLHIV Ever Tested
Women (15-49 years)
Men (15-49 years)
Year

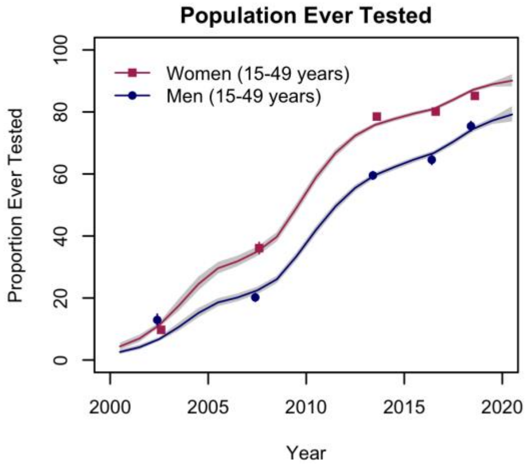
Population Ever Tested
Proportion Ever Tested
Women (15-49 years)
Men (15-49 years)
Year

Zimbabwe*

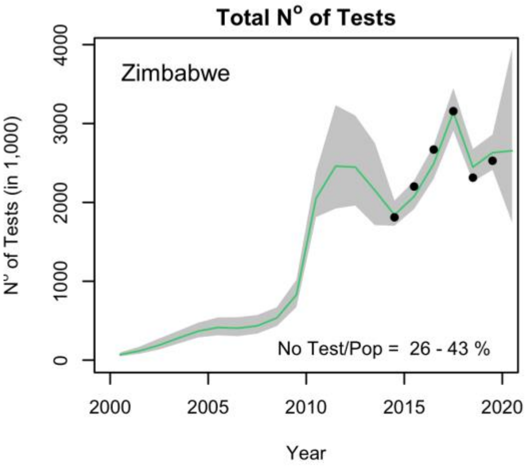

Total N° of Tests
Zimbabwe
N° of Tests (in 1,000)
No Test/Pop = 26 - 43 %
Year

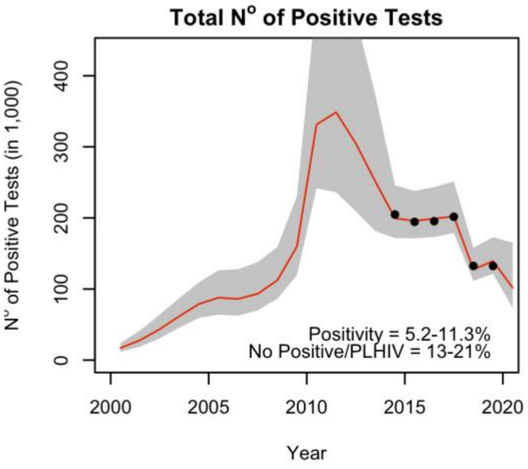

Total N° of Positive Tests
N° of Positive Tests (in 1,000)
Positivity = 5.2-11.3%
No Positive/PLHIV = 13-21%
Year

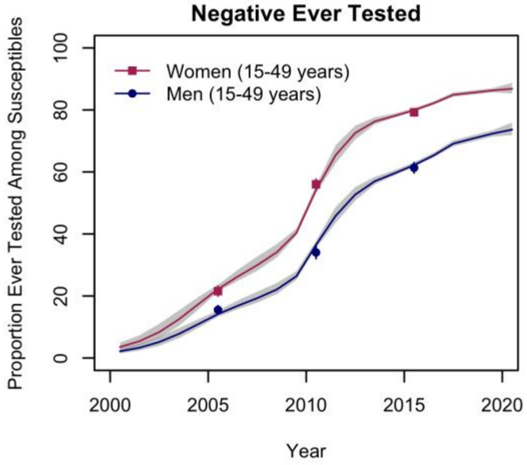

Negative Ever Tested
Proportion Ever Tested Among Susceptibles
Women (15-49 years)
Men (15-49 years)
Year

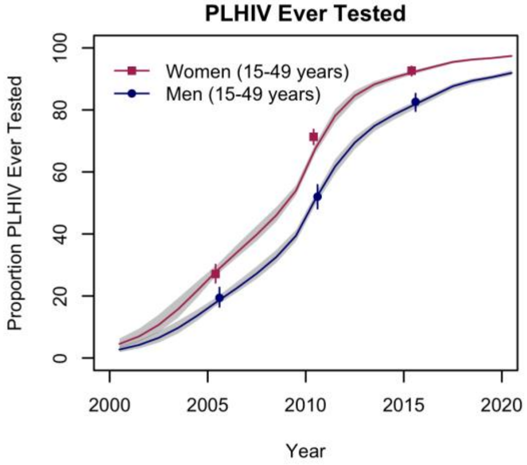

PLHIV Ever Tested
Proportion PLHIV Ever Tested
Women (15-49 years)
Men (15-49 years)
Year

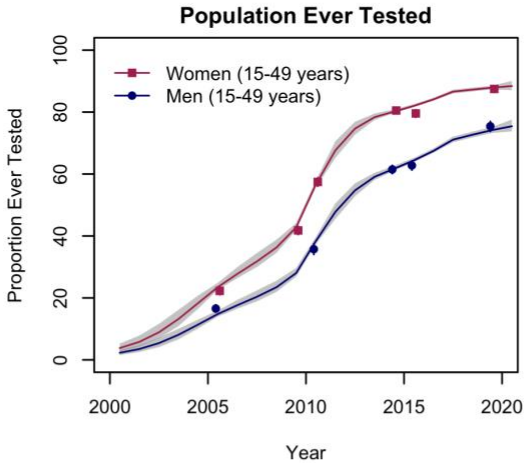

Population Ever Tested
Proportion Ever Tested
Women (15-49 years)
Men (15-49 years)
Year
